# Retroviral vectors encoding ADA regulatory locus control region provide enhanced T-cell-specific transgene expression

**DOI:** 10.1186/1479-0556-7-13

**Published:** 2009-12-30

**Authors:** Alice T Trinh, Bret G Ball, Erin Weber, Timothy K Gallaher, Zoya Gluzman-Poltorak, French Anderson, Lena A Basile

**Affiliations:** 1 Neumedicines Inc, Pasadena, California 91107, USA; 2 Department of Neurosurgery, Mayo Clinic, Rochester, Minnesota 55905, USA; 3 Department of Surgery, Harbor-UCLA Medical Center, Torrance, California 90502, USA

## Abstract

**Background:**

Murine retroviral vectors have been used in several hundred gene therapy clinical trials, but have fallen out of favor for a number of reasons. One issue is that gene expression from viral or internal promoters is highly variable and essentially unregulated. Moreover, with retroviral vectors, gene expression is usually silenced over time. Mammalian genes, in contrast, are characterized by highly regulated, precise levels of expression in both a temporal and a cell-specific manner. To ascertain if recapitulation of endogenous adenosine deaminase (ADA) expression can be achieved in a vector construct we created a new series of Moloney murine leukemia virus (MuLV) based retroviral vector that carry human regulatory elements including combinations of the ADA promoter, the ADA locus control region (LCR), ADA introns and human polyadenylation sequences in a self-inactivating vector backbone.

**Methods:**

A MuLV-based retroviral vector with a self-inactivating (SIN) backbone, the phosphoglycerate kinase promoter (PGK) and the enhanced green fluorescent protein (eGFP), as a reporter gene, was generated. Subsequent vectors were constructed from this basic vector by deletion or addition of certain elements. The added elements that were assessed are the human ADA promoter, human ADA locus control region (LCR), introns 7, 8, and 11 from the human ADA gene, and human growth hormone polyadenylation signal. Retroviral vector particles were produced by transient three-plasmid transfection of 293T cells. Retroviral vectors encoding eGFP were titered by transducing 293A cells, and then the proportion of GFP-positive cells was determined using fluorescence-activated cell sorting (FACS). Non T-cell and T-cell lines were transduced at a multiplicity of infection (MOI) of 0.1 and the yield of eGFP transgene expression was evaluated by FACS analysis using mean fluorescent intensity (MFI) detection.

**Results:**

Vectors that contained the ADA LCR were preferentially expressed in T-cell lines. Further improvements in T-cell specific gene expression were observed with the incorporation of additional *cis*-regulatory elements, such as a human polyadenylation signal and intron 7 from the human ADA gene.

**Conclusion:**

These studies suggest that the combination of an authentically regulated ADA gene in a murine retroviral vector, together with additional locus-specific regulatory refinements, will yield a vector with a safer profile and greater efficacy in terms of high-level, therapeutic, regulated gene expression for the treatment of ADA-deficient severe combined immunodeficiency.

## Background

Severe combined immunodeficiency (SCID) is a pediatric hereditary disorder which affects an individual's T-cells, leaving the individual with practically no immune system. Traditional methods of treatment for SCID include bone marrow transplant or enzyme replacement therapy [1]. These methods are painful, expensive and have a high risk of morbidity and mortality [2-4]. However, gene therapy can offer another potential treatment for SCID. Gene therapy is a very promising treatment for adenosine deaminase severe combined immunodeficiency (ADA-SCID) [5-7], not only because the nature of the disorder makes the patient less likely to reject the vector, but also because it is a well characterized monogenic disorder. Despite advances in the field during the past 30 years, there are still several obstacles to overcome.

Traditional murine retroviral vector designs utilizing viral promoter elements exhibit highly variable and often reduced expression over time, due to silencing, which usually leads to an insufficient therapeutic effect [8-10]. Furthermore, viral promoter elements are constitutively active and may result in unregulated, unsafe, and variable levels of transgene expression. Such unregulated expression may be one reason for the development of a leukemic-like syndrome in five patients recently treated for X-linked SCID (X-SCID) [11-13].

Mammalian genes, in contrast, exhibit highly regulated, stable and precise levels of expression in both a temporal and a cell-type specific manner. Recent studies have begun to elucidate the mechanisms by which large-scale chromatin architecture regulates the expression of individual genes [14,15]. Many mammalian genes use extensive *cis*-regulatory information, such as locus control regions (LCR) and boundary elements, to achieve regulated and cell-specific control of gene expression [16]. Therefore, successful gene therapy of many genetic diseases may require stringent and appropriate control of vector-introduced gene expression. The use of LCRs or related mammalian gene regulatory elements will likely be advantageous, or even essential, for the achievement of safe, consistent, high-level expression of a therapeutic transgene from the context of a gene therapy vector in clinical trials [17].

There has been considerable progress in the development of integrating vectors containing elements of the human β-globin LCR for the treatment of sickle cell anemia and β-thalassemia [18-23]. However, it has previously not been possible to produce high titer murine retroviral vectors containing a human LCR. We report here for the first time the incorporation of key *cis*-regulatory sequences from the human ADA gene [24] into a retroviral vector for the treatment of ADA-SCID (Additional file 1). These vectors contain the human ADA LCR and the human ADA promoter within a self-inactivating (SIN) Moloney murine leukemia virus (MuLV) vector backbone. High titers and T-cell specificity of reporter gene expression were observed. A subset of these vectors contains the human growth hormone polyadenylation signal while further subsets contain introns from the ADA gene. These alterations in vector design have yielded construct-specific increases in T-cell specific gene expression.

These novel vectors demonstrate transcriptional targeting of T-cells and expression of the enhanced green fluorescent protein (eGFP) reporter gene in a manner similar to endogenous ADA gene expression. We believe that vectors capable of authentic gene-specific regulation will prove to be a safer and more effective alternative to previous vectors for the treatment of ADA-SCID and other genetic diseases. The vector constructs presented herein provide a model for a new generation of retroviral vectors capable of cell-type specific gene expression.

## Materials and methods

### Retroviral vector constructions

The MuLV based WTPG (WT = wild type U3; P = phosphoglycerate kinase promoter; G = eGFP reporter gene) vector was constructed by combining the following fragments into the Bluescript KS vector (Stratagene, La Jolla, CA). The 5' LTR and packaging sequences were cloned from pHIT 112 [25] and the 3' LTR and polypurine tract was cloned from pG1 [26]. The PGK promoter was isolated from PGK-Pic20H (from Dr. French Anderson's laboratory, University of Southern California, Dr. Bret Ball's PhD Thesis) by digestion with *BglII *(blunt) and *EcoRI *and ligated into *BamHI *(blunt) and *EcoRI *digested peGFP-N1 (Clontech, Palo Alto, CA). Subsequently the PGK-eGFP gene cassette was isolated by *HindIII *and *NotI *(blunt) digestion and cloned into *HindIII *and *EcoRI *(blunt) sites of WTPG.

SIN 80 is a SIN backbone, cloned from WTPG, which has a 334 bps deletion in U3, leaving 35 bps on the 5' end and 80 bps on the 3' end (deleting all the enhancer and the majority of the CAT box). After amplification by polymerase chain reaction (PCR) with primers 5'GTACCGCTAGCGATATCAGTTCGCTTCTCGC3' and 5'GTAATACGACTCACTATAGGG3', the fragment was digested with *NheI *and *NotI *and inserted into the corresponding sites in the shortened WTPG vector backbone. The *BamHI-XbaI *eGFP fragment (peGFP-N1) was inserted into the *BamHI *and *XbaI *sites to form the intermediate construct SG (S = SIN; G = eGFP reporter gene). The *XbaI*/*SalI *fragment of ADA LCR was digested from the pADA CAT 4/12 vector (the kind gift of Bruce Aronow, University of Cincinnati) [27] and cloned into the *XbaI*/*SalI *site of the SG plasmid to form the intermediate construct SGL (L = human ADA LCR). The ADA promoter was PCR amplified with primers 5'GAACGCTAGCGAGGCTTGCGATGCTCC3' and 5'GAACGCTAGCGCGCGCTCACTTTGGGCT3' and inserted into the *HindIII *and *AgeI *restriction sites of SG and SGL plasmids to construct SAG (A = human ADA promoter) and SAGL. The PGK promoter was digested from PGK-Pic20H using the *HindIII *and *AgeI *restriction enzymes and inserted into the corresponding sites of SG and SGL to generate SPG and SPGL vectors. SLAG and SiLAG (iL = inverted LCR) were constructed by ligation of the *XbaI *(blunt) fragment of the ADA LCR into the *HindIII *(blunt) site of SAG. SAGiL was constructed from SAG and SAGL. The ADA LCR was isolated from SAGL by digestion with *XbaI *(blunt) and *HincII *and inserted into the *HincII *restriction site of SAG.

The next series of vectors constructed contained the ADA promoter (A), eGFP (G), and human growth hormone polyadenylation signal (H) as a cassette in the inverted orientation to generate SHGAL construct. For this purpose, the human growth hormone polyadenylation signal was amplified by PCR from human genomic DNA with primers 5'ATGACCTAGG**GGATCCACCGGTTCTAGAGTTAAC**GTGGCATCCCTGTGAC3' (5' to 3', BamHI, AgeI, XbaI, HincII, bold) and 5'GAAC**AAGCTT**GCCAAGCAAGCAACTCAA3' (HindIII, bold) and subsequently cloned into the *HindIII*/*BamHI *sites of SLAG. The ADA promoter was then introduced into the *BamHI *site and *AgeI *site from the corresponding sites on SAG. The eGFP reporter gene was cloned into the *AgeI *and *XbaI *restriction sites from the corresponding sites on peGFP-N1.

Human ADA introns (I) 7, 8, and 11 were PCR amplified from human genomic DNA. Primers were designed to incorporate a 5' and 3' EcoRV site (bold) for each set of introns 5'GA**GATATC**AGTAAAAGAGGTGAGGGCCTG3' and 5'GA**GATATC**TCCACAGCCTGTAGAGAAGCA3' for intron 7; 5'GA**GATATC**AGGAAAACATGCACTTCGAGG3' and 5'GA**GATATC**GGCAGATCTGAAGAGCAGGT3' for intron 8; 5'GA**GATATC**AGTAAAAGAGGTGAGGGCCTG3' and 5'GA**GATATC**GGCAGATCTGGAAGAGCAGGT3' for introns 7-8; and 5'GA**GATATC**TTCAGCCTCTGCAGGTAGGTT3' and 5'GA**GATATC**TTCTGCCCTGCTCGTTGGTT3' for intron 11. PCR products were digested with *EcoRV *(blunt) and inserted into the *XbaI *site (blunt) of SHGAL to form SHI7GAL, SHI8GAL, SHI78GAL, SHI11GAL and into the *AgeI *site (blunt) to form SHGI7AL, SHGI8AL, SHGI78AL and SHGI11AL.

Two additional plasmids with multiple cloning sites (MCS), MCS-SHGA and MCS-SHGAL, were produced in order to facilitate with the construction of SHGAiL, SLHGA, and SiLHGA. In order to introduce multiple cloning sites upstream to the human growth hormone polyadenylation signal and downstream to the ADA promoter, the forward PCR primer contained sites (5' to 3') SalI, MluI, AsiSI, and NruI (bold) 5'GCGATG**GTCGACACGCG**

**TGCGATCGCTCGCGA**TAGCTTGCCAAGCAAACAACTCAAATGTCC-3' and reverse PCR primer contained sites (5' to 3') SalI, PmeI, PmlI, PacI, and AscI (bold) 5'GCATCC**GTCGACGTTTAAACCACGTGTTAATTAAGGCGCGCC**CTCGAGGCTTGCGATGCTCCCGGGGTC3' were used for HGA fragment amplification from SHGAL. The HGA PCR product was digested with *SalI *and introduced into the *SalI *digested SIN backbone fragment of SiLAG to create MCS-SHGA. Primers 5'GAGTGC**GGCGCGCCCACGTG**T**ATCGAT**A**CTTAAGG**CATGCACCACCATGCCCGGC3' (5' to 3', AscI, PmlI, ClaI, and AflII, bold) and 5'GCTCG**GTTTAAACTTAATTAACACCGGCGAAGCTTGGATCC**ATGCCACATAGCAAGGTGCTGGGTCAC3' (5' to 3', PmeI, PacI, SrgAI, HindIII, and BamHI, bold) were used to incorporate multi-cloning sites into the 5' and 3' of ADA LCR. The PCR product of the ADA LCR amplification was digested with *AscI *and *PmeI *and ligated with the *AscI/PmeI *fragment of MCS-SHGA to create MCS-SHGAL. The ADA LCR was isolated from MCS-SHGAL by digestion with *PacI *and *PmlI *and cloned into MCS-SHGA at the *PacI */*PmlI *or *AsiSI */*NruI *sites to create SHGAiL and SiLHGA respectively. SLHGA was created via isolation of the ADA LCR with *AscI *and *PmeI *then introduced into MCS-SHGA digested with *MluI *and *NruI*. SHGI7AiL, SLHGI7A, and SiLHGI7A were constructed by the same ways as described above with a MCS-SHGI7A and the PCR product of the ADA LCR amplification.

### Cell lines and culture conditions

293T, 293A, NIH3T3, and HeLa (adherent cells, kindly provided by Dr. Paula Cannon, University of Southern California, Keck School of Medicine) [25,28,29] were cultured in Dulbecco's Modified Eagle Medium (Mediatech, Herndon, VA) supplemented with 10% fetal calf serum (Hyclone, Logan, UT, or Gemini Bio-products, Woodland, CA). The adherent rat non T-cell line, XC was also kindly provided by Dr. Paula Cannon [30] and cultured in Minimal Essential Medium (Gibco BRL, Carlsbad CA). Cells were passaged 1:5 every other day or as needed. The non-adherent Molt-4 human T-cell line was kindly provided by Dr. Bruce Aronow (University of Cincinnati) while the non-adherent EL-4 murine T-cell line was obtained from the American Type Culture Collection http://www.atcc.org. CEM, CEM-SS, Jurkat E6-1, and VB non-adherent human T-cell lines were obtained from the NIH AIDS Research and Reference Reagent Program (Rockville MD). All T-cell lines were grown in RPMI 1640 (Mediatech, Herndon, VA) supplemented with 10% fetal calf serum.

### Production of virus containing cell culture supernatant and determination of viral titer

Retroviral vector particles were produced by transient three-plasmid transfection of 293T cells by overnight calcium phosphate treatment on 10 cm dishes seeded the previous day to give a maximum of 70% confluence/plate on the day of transfection [23,25,31]. For each transfection, 10-50 μg of vector plasmid, 10 μg of pol-gag (pCGP) packaging plasmid [25], and 2 μg of vesicular stomatitis virus G protein (pVSV-G) envelope plasmid [25] (both from Dr. Paula Cannon, University of Southern California) were used. Twelve to sixteen hours after transfection, cells were gently washed with PBS, warmed to 37°C, and 6 ml of fresh medium with 10 mM sodium butyrate was added. After 8 hours of incubation, the medium was replaced with 6 ml of fresh medium. Thirty-six hours after transfection, supernatants containing viral particles were harvested and passed through a 0.45-micron filter (Millipore, Bedford, MA) to remove cells and cellular debris. Polybrene was added to filtered vector supernatants to a final concentration of 8 μg/ml, and then the vectors were stored in aliquots at -80°C.

All retroviral vectors encoding eGFP were titered by transducing 293A cells, as described below, with serial dilutions of the vectors preparations. 293A cells were plated 16 hours prior to transduction at a concentration of 3-5 × 10^5 ^cells per well in 6-well plates. On the day of transduction, five serial 1:3 dilutions of thawed virus containing supernatant were prepared; 1 ml of each dilution were directly added to the cells and incubated at 37°C for 4 to 8 hours. After incubation, vector supernatants were removed and fresh medium was added. Cells were cultured for 2 to 3 days, then trypsinized, PBS washed, and fixed with 4% paraformaldehyde (pH 7.2) before they were analyzed for transgene expression by fluorescent-activated cell sorting (FACS) [32] on the Beckman Coulter Epic XL-MCL to enumerate the proportion of GFP-positive cells. In order to compensate for autofluorescence of untransduced cells, two-dimensional FACS gating was used. Titers were routinely determined from a volume of vector preparation that yielded linear, dose dependent transduction of target cells with a level not in excess of 20% [33]. The ranges of titers obtained from 2 to 24 independent titrations for each vector are presented in Table 1.

**Table 1:** Comparison of virus titers of MuLV SIN vectors in 293T cells.

**A**.		**B**.	
Vector	Titer (cfu/ml)	Vector	Titer (cfu/ml)
WTPG	1.8-2.0 × 10^7^	SHGAL	0.12-2.9 × 10^5^
SPG	8.0 × 10^6^	SHGAiL	0.4-1.5 × 10^6^
SPGL	1.0 × 10^6^	SLHGA	0.3-1.1 × 10^5^
SAG	0.8-1.5 × 10^7^	SiLHGA	3.5-7.3 × 10^4^
SAGL	2.0-3.1 × 10^6^	SHGI7AL	0.07-2.6 × 10^5^
SAGiL	4.8 × 10^5^	SHGI7AiL	0.4-2.1 × 10^6^
SLAG	0.5-1.0 × 106	SLHGI7A	0.3-1.4 × 10^5^
SiLAG	1.1-2.9 × 10^6^	SiLHGI7A	0.4-1.5 × 10^5^

The ranges of titers obtained from 2 to 24 independent titrations for each vector are presented.

### Cell transduction and FACS analysis of eGFP expression

Adherent cells were plated 16 hours prior to transduction at a concentration of 3-5 × 10^5 ^cells per well in 6-well plates and non-adherent cells were plated the day of transduction at a concentration of 1 × 10^6 ^cells per well in 6-well plates. All cells were transduced at a multiplicity of infection (MOI) of 0.1 infectious viral particles per cell and incubated for two to three weeks to allow stabilization of gene expression and to eliminate non-integrating proviral remnants. After two to three weeks of culture, adherent cells were treated as described above and non-adherent cells were PBS washed and fixed with 4% paraformaldehyde. For each sample, 200,000 events were analyzed by FACS to obtain the mean florescent intensity (MFI) and the percentage of eGFP positive cells. Two to eight independent experiments were performed in duplicates; mean values with corresponding standard errors of the mean were calculated unless indicates otherwise.

## Results

### Construction of Moloney MuLV SIN vectors containing the human ADA promoter LCR

**Figure 1:**
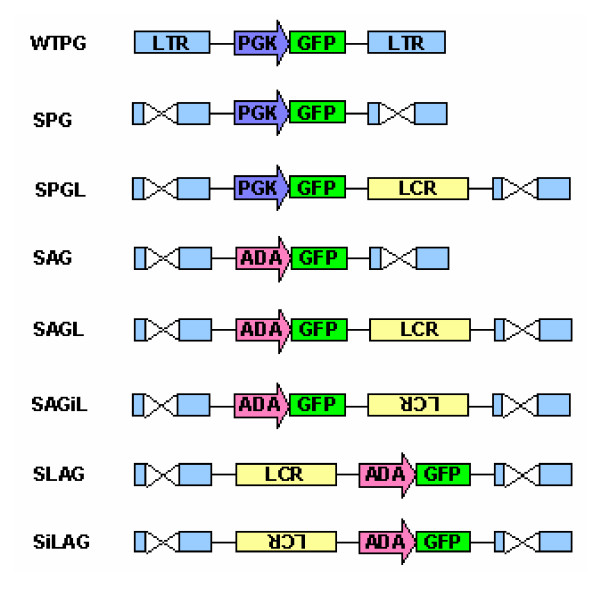
**Proviral configuration of MuLV SIN vector constructs incorporating the human ADA LCR**. A series of eight vectors were constructed in order to test the functionality of the human ADA LCR in the context of a retroviral vector. WTPG, SPG, and SAG are control vectors. SAGL contains the LCR 3' to the ADA promoter and eGFP gene while SAGiL has an inverted LCR in the same position. SPGL examines the ability of the LCR to function with the heterologous PGK promoter. SLAG contains the LCR 5' to the ADA promoter in the forward direction while SiLAG contains the LCR inserted in the reverse orientation. WT = U3 region is wild type; S = SIN vector with deletion in 3' U3; P = PGK promoter; G = eGFP reporter gene; A = human ADA promoter; L = human ADA LCR.

Initially, we undertook to incorporate the human ADA LCR, together with its natural ADA promoter, into the MuLV retroviral vector backbone. The first series of vector constructs were made with the LCR in various configurations and orientations (Figure 1). WTPG is a standard non-SIN MuLV retroviral vector in which the PGK promoter is used to drive expression of the eGFP reporter gene. SPG and SAG contain either the PGK promoter or the ADA promoter within a SIN MuLV vector backbone [34]. These vectors were used to establish the baseline gene expression level of eGFP. SAGL is the experimental vector incorporating the ADA LCR 3' to the ADA promoter while SPGL assesses the ability of the LCR to affect expression from a heterologous promoter, PGK. These constructs demonstrated that the ADA LCR can be successfully incorporated into retroviral vectors at reasonably high titers of 10^5^-10^7 ^infectious virus particles/ml (Table 1A).

**Figure 2:**
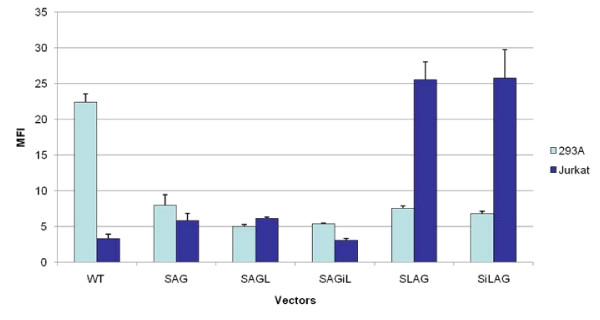
**ADA LCRs function in an orientation-independent but position-dependent manner within a retroviral vector**. 293A or Jurkat cells were transducted by different vectors (WTPG, SAG, SAGL, SAGiL, SLAG and SiLAG) expressing eGFP reporter gene at MOI of 0.1. The mean florescent intensity (MFI) of eGFP expression was evaluated using FACS 3 weeks post-transduction.

The ADA LCR was also inserted into SAG 5' to the ADA promoter in both the forward and reverse orientations, forming SLAG and SiLAG (Figure 1). Classically, enhancers function in an orientation- and position-independent manner, suggesting that the enhancer function of the LCR should not be affected by an alteration in location or orientation with respect to the target promoter [35]. However, some evidence has shown that LCRs may not always be position- or orientation-independent [36]; SiLAG and SAGiL test this property. Results, shown in Figure 2, suggest that the ADA LCR is orientation-independent, in that the LCR in the inverted direction within both SAGiL and SiLAG does not substantially affect reporter gene expression in 293T or Jurkat cells, when compared to SAGL and SLAG, respectively. Thus, the ADA LCR displays a property of classical enhancers, as it functions in an orientation-independent manner in the context of these retroviral vectors.

However, there is a strong preference for the ADA LCR to be positioned 5' to the ADA promoter, as seen in Figure 2, where vectors containing the ADA LCR 5' to the ADA promoter show highly enhanced, preferential gene expression in the Jurkat human T-cell line as compared to 293A cells. Thus, the ADA LCR does appear to possess position-dependence, which is orientation independent with respect to the ADA promoter.

### Vectors containing the ADA LCR demonstrate high-level gene expression in T-cell lines

**Figure 3:**
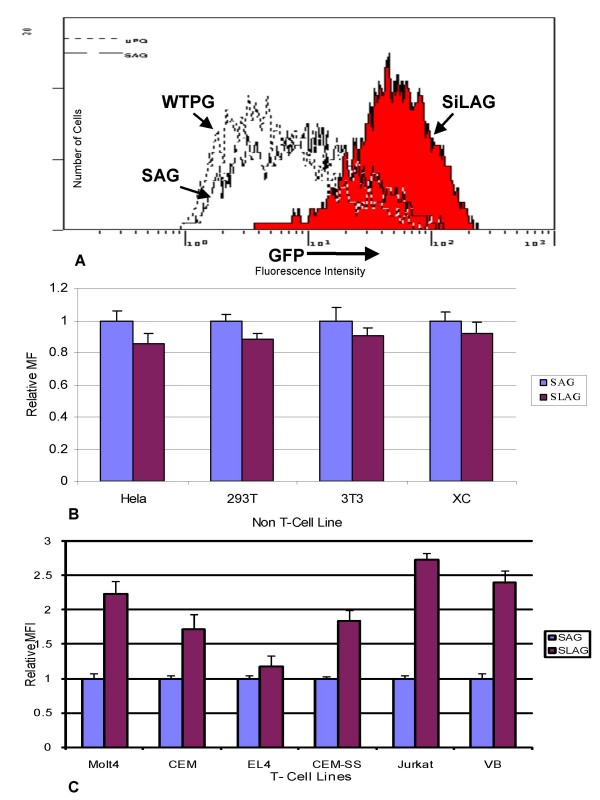
**Vectors Containing the ADA LCR 5' to the ADA Promoter Show Cell Specific Enhanced Expression**. **A**. Enhanced expression of eGFP under the SiLAG vector in Jurkat cells, a human T-cell line, as determined by FACS analysis. **B**. LCR-mediated enhancement of eGFP expression in both human and murine T-cell lines. **C**. The ADA LCR does not enhance expression of eGFP in non-T-cell lines. MFI of eGFP expression was measured by FACS and normalized to the MFI of SAG transduced cells.

One measure of LCR function is the ability to generate expression in a cell-specific manner. In some cell types, traditional retroviral vectors are known to express poorly despite successful integration [9]. Such cells exhibit lower relative titers due to this failure of expression [37]. A graph of the MFI of various vectors transduced in Jurkat cells demonstrates that ADA LCR containing vector, SiLAG yields higher cell-specific expression levels in Jurkat cells than the non-LCR counterparts, namely SAG and WTPG (Figure 3A).

To further evaluate this effect, additional human T-cell lines (Molt4, CEM, CEM-SS, and VB), the murine EL4 T-cell line, and non-T-cell lines (HeLa, 293T, 3T3, and XC) were used to assess the cell-type specific gene expression for the vector SAG and the best performing vector thus far in the Jurkat T-cell line, namely SLAG. When the ADA LCR was included in the vector, enhancement of reporter gene expression was observed in all T-cell lines tested, suggesting that the ADA LCR is functioning to increase gene expression in T-cell lines (Figure 3B). Incorporation of the ADA LCR did not result in enhanced gene expression in non-T-cell lines, but rather a slight reduction, (Figure 3C). Thus, the ADA LCR is functioning in a cell-type specific manner.

### The incorporation of additional cis-regulatory elements, in combination with the ADA LCR, increases eGFP expression in T-cell lines

**Figure 4:**
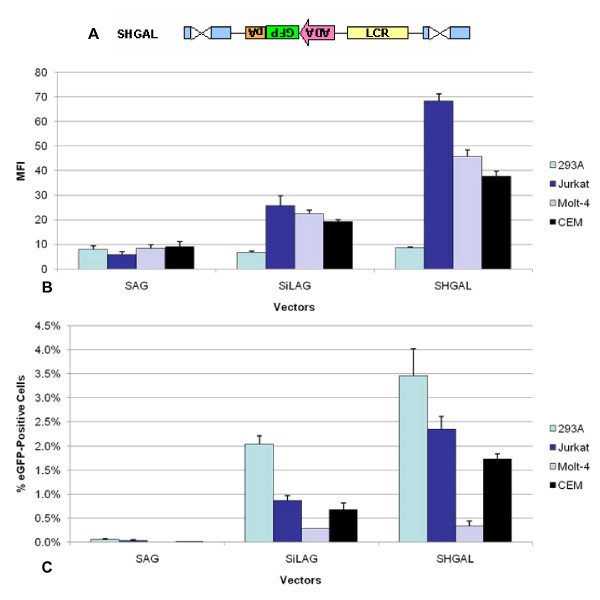
**Additional *cis*-regulatory elements further improve vector gene expression**. **A**. SHGAL contains the human growth hormone polyadenylation signal (H) (pA in orange) 5' to the eGFP. 293A, Jurkat, Molt-4 and CEM cells were transduced by SAG, SiLAG or SHGAL vectors at MOI of 0.1. The MFI of eGFP expression (**B**) and the percentage of eGFP positive cells (**C**) were evaluated using FACS analysis.

Previous studies have shown that successful lentiviral vectors have been developed with the β-globin promoter, gene and a polyadenylation signal placed in reverse orientation to the vector element and the β-globin LCR in the direct orientation 3' to these elements for increased stability [18]. Therefore, a similar arrangement was used for our studies; the ADA promoter, eGFP reporter gene, and human growth hormone polyadenylation signal were placed in reverse orientation within the vector. The LCR was placed in the forward orientation adjacent to the promoter to create a novel vector, named SHGAL (Figure 4A). Given the inverse orientation of the LCR with respect to the ADA promoter, the SHGAL vector is configured similarly to the SiLAG vector, which showed preferentially high reporter gene expression in T-cell lines.

The addition of the human growth hormone polyadenylation signal reduced the titer by approximately one log compared to the non-LCR containing vector, SAG, but the SHGAL vector was still able to maintain a relatively high titer of 8.5 × 10^4 ^in 293A cells (Table 1A, 1B). Although SHGAL displayed slightly reduced titer, the MFI in Jurkat cells had an 11.8 and 2.7-fold enhancement over levels observed in SAG and SiLAG, respectively. Enhanced gene expression was also observed in other T-cell lines such as CEM and Molt-4, however, expression levels remained unchanged in the non T-cell line, 293A (Figure 4B). The percentage of eGFP positive cells noticeably increased with elemental changes made to SAG, SiLAG, and SHGAL in both non-T-cell and T-cell lines (Figure 4C). However, an enhancement of gene expression was observed only in T-cell lines (Figure 4B). Thus, our studies revealed that the use of an inverted gene expression cassette with a human polyadenylation signal, placed in an inverted configuration, similar to that used in other integrating vectors to generate tissue-specific expression of the β-globin gene, yields substantially increased levels of tissue-specific gene expression.

### The addition of intron 7 further increases eGFP expression in a cell-type specific manner

**Figure 5:**
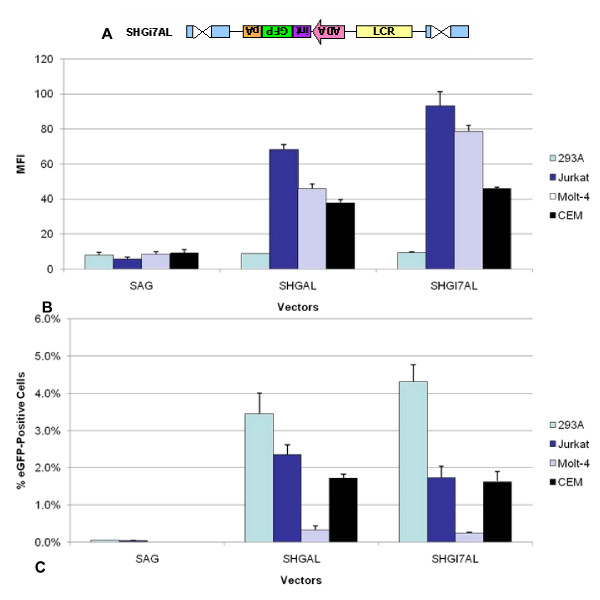
**Insertion of intron 7 of the ADA gene results in further enhancement of gene expression**. **A**. SHGI7AL contains intron 7 of the ADA gene in the inverted reverse orientation with respect to the vector elements. 293A, Jurkat, Molt-4 and CEM cells were transduced by SAG, SHGAL or SHGI7AL vectors at MO of 0.1. The MFI of eGFP expression (**B**) and the percentage of eGFP positive cells (**C**) were evaluated using FACS analysis.

The inclusion of an intron within a retroviral vector has been previously reported to facilitate processing of the mRNA transcripts, thus resulting in enhanced transgene expression [38]. In an attempt to further increase titer and MFI, three introns (7, 8, and 11) and a pair of introns (7 and 8) from the human ADA gene were introduced into the SHGAL vector. Small introns were chosen in light of the size limitations of a retroviral vector genome [39]. The introns were inserted either 5' or 3' to the eGFP gene to form the following vectors: SHI7GAL, SHI8GAL, SHI7+8GAL and SHI11GAL; and SHGI7AL, SHGI8AL, SHGI11AL, and SHGI7+8AL. Of theses vectors, only SHGI7AL (Figure 5A) demonstrated improved expression over the parental vector, SHGAL (**data not shown**). However, the insertion of intron 7 did not yield a significant change in 293T-derived titer as compared to SHGAL (Table 1B).

A comparison of the SHGI7AL versus the SHGAL vector in various T-cell lines and in the non-T-cell line, 293A, is shown in Figure 5B. The SHGI7AL vector yielded enhanced gene expression in all the T-cell lines tested, namely Jurkat, Molt-4 and CEM, as compared to SHGAL. Even though the number of positive cells is highest in 293A cells (Figure 5C), the level of gene expression is not enhanced in these non-T-cell line for SHGI7AL, as shown in Figure 5B, thus demonstrating that the gene is being expressed in a cell-type specific manner.

From prior studies, as well as the results of the present study, we see that an LCR is not always position-independent. Hence, several more constructs were created to test this property and to discern if further improvements could be made for titer and/or expression levels. The constructs generated were variations of vectors SHGAL and SHGI7AL with the LCR either in the forward or reverse orientation and located 3' to the polyadenylation signal or with the LCR in the reverse orientation as compared with SHGAL and SHGI7AL.

**Figure 6:**
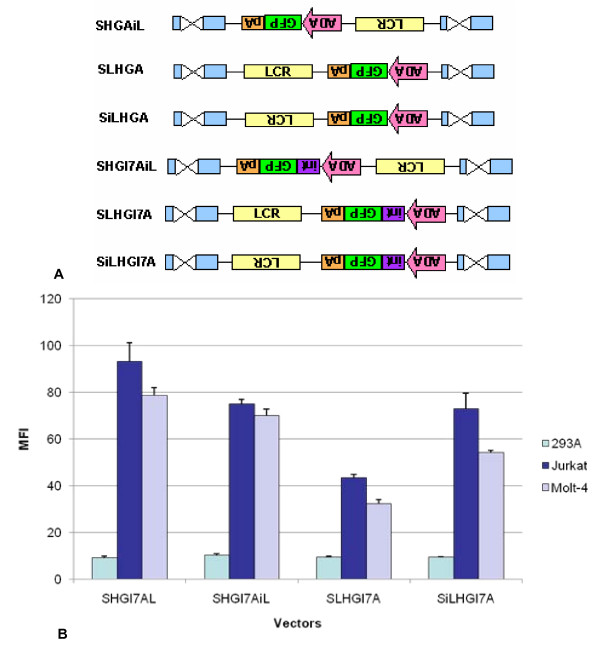
**SHGI7AL show no improved expression with the LCR in various positions and orientations**. **A**. SHGI7AL contains intron 7 inserted 5' to the ADA promoter, while SHGI7AiL contains the LCR in the reverse orientation. SLHGA, SLHGI7A, SiLHGA and SiLHGI7A possess the LCR 5' to the polyadenylation signal in both the forward and reverse direction respectively. These vectors were created to examine if the LCR functions independently of position and orientation. **B**. 293A, Jurkat, and Molt-4 cells were transduced by SHGI7AL, SHGI7AiL, SLHGI7A or SiLHGI7A vectors at MOI of 0.1. The MFI of eGFP expression was evaluated using FACS analysis.

SHGAiL and SHGI7AiL, which have the LCR is in the inverted orientation, produced titers that surpassed the parent vectors, SHGAL and SHGI7AL, by one log (Table 1B). SLHGA, SiLHGA, SLHGI7A and SiLHGI7A, in which the LCR was positioned 3' to the polyadenylation signal (Figure 6A), all failed to show improvements in titers and, in fact, yielded the poorest titers of all the vectors in the second series (Table 1B). Vectors containing intron 7 showed gradual improvements in MFI over SHGAL, except for SLHGI7A, but none of these vector configurations was able to exceed the MFI of SHGI7AL (Figure 6B). As was observed for all LCR-containing vectors to date, there was no enhancement in gene expression in the non-T-cell line, 293A.

## Discussion

Previous clinical trials have faced obstacles to successful gene therapy in three key areas: gene delivery, gene expression, and safety. The purpose of this study was to develop a new generation of murine retroviral vectors in which the viral regulatory signals are replaced with gene-specific human regulatory sequences in order to generate safer and more effective treatments for inherited immunodeficiencies. Many clinical trials have utilized retroviral vectors because their efficiency of gene delivery and capacity for integration into the host genome provide the greatest potential for long-term correction of a disease. However, there are a number of intrinsic problems and risks associated with using the traditional MuLV retroviral vector, as discussed by Baum *et al*. [17]. Recent advances in our understanding of cellular transcriptional regulation are facilitating the development of vectors completely regulated by human sequences which can generate appropriate and authentic expression of a therapeutic gene. Improvements in retroviral vector design are necessary to incorporate human regulatory sequences without adversely affecting gene transfer efficiency. Vectors with incorporated sequences that correspond to specific endogenous regulatory elements should prove to be safer and more effective than traditional retroviral vector designs due to more authentic regulation, which should result in less promiscuity and less variable expression.

The LCR from the ß-globin gene and its incorporation into lentiviral vectors has been used as a model system by a number of laboratories [18-23]. Recent work demonstrated considerable success [18,19]. Earlier studies using murine retroviral vectors and ß-globin elements encountered a number of problems including: 1) vector instability, with a high frequency of deletions and rearrangements, 2) inauthentic gene expression patterns, and 3) low levels of gene expression often accompanied by high levels of clonal variation and the silencing of expression over time [40-42]. In light of these difficulties, the successful use of lentiviral vectors for the treatment of ß-globinopathies has been a significant advancement. However, there are situations in which a lentiviral vector may not be ideal for gene transfer. While lentiviral vectors do exhibit a number of advantages over the traditional murine retroviral vector, there are, nonetheless, significant safety concerns about administering a lentivirus-based vector to a human patient, particularly for the treatment of a genetic disease in a child as opposed to the treatment of HIV or cancer in an adult. Therefore, as the long-term clinical effects of a lentiviral vector remain largely unknown, the development of a safe and efficacious murine retroviral vector could have a role in the treatment of a number of genetic diseases.

The model disease for testing gene therapy treatments has traditionally been SCID, caused either by ADA or γ_c _chain deficiencies [6,7,43,44]. These two genetic diseases are ideal because the administration of gene-corrected cells results in a positive selective advantage and preferential survival of "normal" gene-corrected cells [43]. Development of a leukemia-like syndrome in five patients following successful treatment of SCID, however, has demonstrated that unregulated gene expression is a potential danger, particularly when coupled with vector insertion adjacent to a potential proto-oncogene [11-13,45]. Even though this is a tragic unforeseen development, two of the five children have been successfully treated for leukemia while another child is currently receiving treatment [12,13].

Our studies indicate that the addition of the 2.3 kb ADA LCR placed within a SIN backbone is able to provide T-cell-specific gene expression in a retroviral vector with minimal effect on titer. This is observed in the SLAG and SiLAG vector construct, which both exhibited improvements in MFI by a 4.4-fold increase compared to SAG in the T-cell line, Jurkat. This increase in gene expression also was observed in other T-cell lines such as Molt-4 and CEM. Moreover, the introduction of additional *cis*-regulatory sequences, such as a human polyadenylation signal for the SHGAL vector or the introduction of both the human polyadenylation signal and intron 7 from the human ADA gene for the SHGi7AL vector further improved T-cell specific transgene expression, but displayed some decrease in titers in 293T cells, as compared to vectors containing only the LCR. The MFI of SHGAL and SHGI7AL was increased over 11.8 and 16.1-fold, respectively in Jurkat cells as compared to SAG. With the LCR in the inverted orientation, as seen in SHGAiL or SHGI7AiL, titer levels were restored, however the MFI was slightly decreased compared to SHGAL and SHGI7AL. Overall, the results show that the addition of accessory *cis*-regulatory elements can improve gene expression in a cell-type specific manner using a retroviral vector.

The extensive studies on the β-globin LCR in lentiviral vectors have demonstrated that by proper placement of optimal-sized LCR elements, it is possible to obtain a high level of globin expression in a significant percentage of erythroid cells in mice at an average vector copy number of 1-2 [18]. Thus, gene therapy vectors are reaching a level of sophistication that will allow clinical trials for sickle cell anemia and β-thalassemia. By incorporation of the ADA LCR into retroviral vectors, we have demonstrated that T-cell-specific gene expression can be obtained *in vitro*, suggesting that similar effects may be achieved *in vivo*. The next step is to test these humanized ADA LCR-containing vectors in primary cells, such as bone marrow cells and various populations of stem cells, followed by the transplantation of these transduced primary cells into a mouse model. We believe that these vectors have the potential to provide a safer and more effective treatment option for genetic immunodeficiencies.

## Conclusion

We have developed a series of fully humanized vector constructs containing several ADA regulatory elements. Vector SHGI7AL, which contains the human polyadenylation growth hormone, intron 7 from the human ADA gene, the ADA promoter and the ADA LCR, proved to show the highest level of expression compared with the other vectors. Even though the titer for this vector is not as high as some of the other vectors, this can be overcome by titer concentrations.

Vectors capable of authentic gene-specific regulation may prove to be a safer and more effective alternative to previous vectors for the treatment of ADA-SCID through more regulated, higher levels of gene expression. Furthermore, together with studies on the human β-globin LCR, the vector constructs presented herein shed light on the possibility of generating a global model construct for the creation of retroviral vectors capable of cell-specific expression for the treatment of various genetic diseases.

## Abbreviations

ADA: adenosine deaminase; MuLV: Moloney murine leukemia virus; LCR: locus control region; PGK: phosphoglycerate kinase promoter; eGFP: enhanced green fluorescent protein; SIN: self-inactivating; FACS: fluorescence-activated cell sorting; MOI: multiplicity of infection; MFI: mean fluorescent intensity; SCID: severe combined immunodeficiency; WT: wild type; P: phosphoglycerate kinase; G: enhanced green fluorescent protein reporter gene; S: self-inactivating; L: ADA locus control region; A: human ADA promoter; iL: inverted ADA locus control region; H: human growth hormone polyadenylation signal; I: intron; MCS: multicloning site.

## Competing interests

The authors declare that they have no competing interests.

## Authors' contributions

ATT carried out the experimental work, data analysis and revised the manuscript. BB also carried out the experimental work, data analysis and drafted the manuscript. Both BB and ATT contributed an equal amount of work to the project and share co-first authorship. EW participated in the experimental design and reviewed the manuscript. TKG and ZGP reviewed and helped revise the manuscript. FA conceived of the study and reviewed the manuscript. LAB participated in the design, coordination of the study, data analysis and reviewed the manuscript. All authors have read and approved of the final manuscript.

## Supplementary Material

Additional file 1 **Construct sequences.** Full sequences of the constructs and the chromosomal locations of the inserted control sequences (ADA promoter, ADA LCR, human growth hormone polyadenylation sequence and intron 7 of ADA). Click here for file
