# Supplementary material for: Retroviral vectors encoding ADA regulatory locus control region provide enhanced T-cell-specific transgene expression
Source: Genet Vaccines Ther. 2009 Dec 30;7:13. doi: 10.1186/1479-0556-7-13 (PMC2809042; doi:10.1186/1479-0556-7-13)
Supplement: Additional file 1 — Construct sequences. Full sequences of the constructs and the chromosomal locations of the inserted control sequences (ADA promoter, ADA LCR, human growth hormone polyadenylation sequence and intron 7 of ADA). [file 1479-0556-7-13-S1.DOC]

**Additional Information**

**Chromosomal locations of inserted regulatory regions used in these vectors are summarized below.**

Locations are based on the International Human Genome Sequencing Consortium March 2006 assembly human genome reference sequence (NCBI Build 36.1) as implemented at the University of California Santa Cruz Human Genome BLAT server

ADA Promoter sequence Chr 20 q13.12 bases 42,713,662 - 42,714,123

ADA LCR Chr 20 q 13.12 bases 42,707,107 - 42,709,367

ADA intron 7 Chr 20 q13.12 bases 42,684,978 - 42,685,071

HGH polyA site Chr 17 q23.3 bases 59,310,831 - 59,311,404

**Sequences of vectors are as follows.**

**WTPG**

5851 bp

5’LTR CMV promoter 1-530

R region 531-589

U5 region 590-665

Packaging signal 666-1042

PGK promoter 1052-1531

eGFP gene 1559-2278

3’LTR U3 region 2337-2786

R region 2787-2853

U5 region 2854-2930

T7 primer site 3012-2981

CGTTACATAACTTACGGTAAATGGCCCGCCGGCTGACCGCCCAACGACCCCCGCCCATTG

ACGTCAATAATGACGTATGTTCCCATAGTAACGCCAATAGGGACTTTCCATTGACGTCAA

TGGGTGGAGTATTTACGGTAAACTGCCCACTTGGCAGTACATCAAGTGTATCATATGCCA

AGTACGCCCCCTATTGACGTCAATGACGGTAAATGGCCCGCCTGGCATTATGCCCAGTAC

ATGACCTTATGGGACTTTCCTACTTGGCAGTACATCTACGTATTAGTCATCGCTATTACC

ATGGTGATGCGGTTTTGGCAGTACATCAATGGGCGTGGATAGCGGTTTGACTCACGGGGA

TTTCCAAGTCTCCACCCCATTGACGTCAATGGGAGTTTGTTTTGGCACCAAAATCAACGG

GACTTTCCAAAATGTCGTAACAACTCCGCCCCATTGACGCAAATGGGCGGTAGGCGTGTA

CGGTGGGAGGTCTATATAAGCAGAGCTCGTTTAGTGAACCGCGCCAGTCTTCCGATAGAC

TGCGTCGCCCGGGTACCCGTATTCCCAATAAAGCCTCTTGCTGTTTGCATCCGAATCGTG

GTCTCGCTGTTCCTTGGGAGGGTCTCCTCTGAGTGATTGACTACCCACGACGGGGGTCTT

TCATTTGGGGGCTCGTCCGGGATTCGGAGACCCCTGCCCAGGGACCACCGACCCACCACC

GGGAGGTAAGCTGGCCACCAACTTATCTGTGTCTGTCCGATTGTCTAGTGTCTATGACTG

ATTTTATGCGCCTGCGTCGGTACTAGTTAGCTAACTAGCTCTGTATCTGGCGGACCCGTG

GTGGAACTGACGAGTTCGGAACACCCGGCCGCAACCCTGGGAGACGTCCCAGGGACTTCG

GGGGCCGTTTTTGTGGCCCGACCTGAGTCCAAAAATCCCGATCGTTTTGGACTCTTTGGT

GCACCCCCCTTAGGAGGAGGGATATGTGGTTCTGGTAGGAGACGAGAACCTAAAACAGTT

CCCGCCTCCGTCTGAATTTTTGCTAAGCTTCGAATTCCGGGGTTGGGGTTGCGCCTTTTC

CAAGGCAGCCCTGGGTTTGCGCAGGGACGCGGCTGCTCTGGGCGTGGTTCCGGGAAACGC

AGCGGCGCCGACCCTGGGTCTCGCACATTCTTCACGTCCGTTCGCAGCGTCACCCGGATC

TTCGCCGCTACCCTTGTGGGCCCCCCGGCGACGCTTCCTCGTCCGCCCCTAAGTCGGGAA

GGTTCCTTGCGGTTCGCGGCGTGCCGGACGTGACAAACGGAAGCCGCACGTCTCACTAGT

ACCCTCGCAGACGGACAGCGCCAGGGAGCAATGGCAGCGCGCCGACCGCGATGGGCTGTG

GCCAATAGCGGCTGCTCAGCAGGGCGCGCCGAGAGCAGCGGCCGGGAAGGGGCGGTGCGG

GAGGCGGGGTGTGGGGCGGTAGTGTGGGCCCTGTTCCTGCCCGCGCGGTGTTCCGCATTC

TGCAAGCCTCCGGAGCGCACGTCGGCAGTCGATCTAGATCGATCCACCGGTCGCCACCAT

GGTGAGCAAGGGCGAGGAGCTGTTCACCGGGGTGGTGCCCATCCTGGTCGAGCTGGACGG

CGACGTAAACGGCCACAAGTTCAGCGTGTCCGGCGAGGGCGAGGGCGATGCCACCTACGG

CAAGCTGACCCTGAAGTTCATCTGCACCACCGGCAAGCTGCCCGTGCCCTGGCCCACCCT

CGTGACCACCCTGACCTACGGCGTGCAGTGCTTCAGCCGCTACCCCGACCACATGAAGCA

GCACGACTTCTTCAAGTCCGCCATGCCCGAAGGCTACGTCCAGGAGCGCACCATCTTCTT

CAAGGACGACGGCAACTACAAGACCCGCGCCGAGGTGAAGTTCGAGGGCGACACCCTGGT

GAACCGCATCGAGCTGAAGGGCATCGACTTCAAGGAGGACGGCAACATCCTGGGGCACAA

GCTGGAGTACAACTACAACAGCCACAACGTCTATATCATGGCCGACAAGCAGAAGAACGG

CATCAAGGTGAACTTCAAGATCCGCCACAACATCGAGGACGGCAGCGTGCAGCTCGCCGA

CCACTACCAGCAGAACACCCCCATCGGCGACGGCCCCGTGCTGCTGCCCGACAACCACTA

CCTGAGCACCCAGTCCGCCCTGAGCAAAGACCCCAACGAGAAGCGCGATCACATGGTCCT

GCTGGAGTTCGTGACCGCCGCCGGGATCACTCTCGGCATGGACGAGCTGTACAAGTAAAG

CGGCCATCCCATCGATAAAATAAAAAGATTTTATTTAGTCTCCAGAAAAAGGGGGGAATG

AAAGACCCCACCTGTAGGTTTGGCAAGCTAGCTTAAGTAACGCCATTTTGCAAGGCATGG

AAAAATACATAACTGAGAATAGAGAAGTTCAGATCAAGGTCAGGAACAGATGGAACAGCT

GAATATGGGCCAAACAGGATATCTGTGGTAAGCAGTTCCTGCCCCGGCTCAGGGCCAAGA

ACAGATGGAACAGCTGAATATGGGCCAAACAGGATATCTGTGGTAAGCAGTTCCTGCCCC

GGCTCAGGGCCAAGAACAGATGGTCCCCAGATGCGGTCCAGCCCTCAGCAGTTTCTAGAG

AACCATCAGATGTTTCCAGGGTGCCCCAAGGACCTGAAATGACCCTGTGCCTTATTTGAA

CTAACCAATCAGTTCGCTTCTCGCTTCTGTTCGCGCGCTTCTGCTCCCCGAGCTCAATAA

AAGAGCCCACAACCCCTCACTCGGGGCGCCAGTCCTCCGATTGACTGAGTCGCCCGGGTA

CCCGTGTATCCAATAAACCCTCTTGCAGTTGCATCCGACTTGTGGTCTCGCTGTTCCTTG

GGAGGGTCTCCTCTGAGTGATTGACTACCCGTCAGCGGGGGTCTTTCATTTGGGGGCTCG

TCCGGGATCGCGGCCGCCACCGCGGTGGAGCTCCAATTCGCCCTATAGTGAGTCGTATTA

CGCGCGCTCACTGGCCGTCGTTTTACAACGTCGTGACTGGGAAAACCCTGGCGTTACCCA

ACTTAATCGCCTTGCAGCACATCCCCCTTTCGCCAGCTGGCGTAATAGCGAAGAGGCCCG

CACCGATCGCCCTTCCCAACAGTTGCGCAGCCTGAATGGCGAATGGGACGCGCCCTGTAG

CGGCGCATTAAGCGCGGCGGGTGTGGTGGTTACGCGCAGCGTGACCGCTACACTTGCCAG

CGCCCTAGCGCCCGCTCCTTTCGCTTTCTTCCCTTCCTTTCTCGCCACGTTCGCCGGCTT

TCCCCGTCAAGCTCTAAATCGGGGGCTCCCTTTAGGGTTCCGATTTAGTGCTTTACGGCA

CCTCGACCCCAAAAAACTTGATTAGGGTGATGGTTCACGTAGTGGGCCATCGCCCTGATA

GACGGTTTTTCGCCCTTTGACGTTGGAGTCCACGTTCTTTAATAGTGGACTCTTGTTCCA

AACTGGAACAACACTCAACCCTATCTCGGTCTATTCTTTTGATTTATAAGGGATTTTGCC

GATTTCGGCCTATTGGTTAAAAAATGAGCTGATTTAACAAAAATTTAACGCGAATTTTAA

CAAAATATTAACGCTTACAATTTAGGTGGCACTTTTCGGGGAAATGTGCGCGGAACCCCT

ATTTGTTTATTTTTCTAAATACATTCAAATATGTATCCGCTCATGAGACAATAACCCTGA

TAAATGCTTCAATAATATTGAAAAAGGAAGAGTATGAGTATTCAACATTTCCGTGTCGCC

CTTATTCCCTTTTTTGCGGCATTTTGCCTTCCTGTTTTTGCTCACCCAGAAACGCTGGTG

AAAGTAAAAGATGCTGAAGATCAGTTGGGTGCACGAGTGGGTTACATCGAACTGGATCTC

AACAGCGGTAAGATCCTTGAGAGTTTTCGCCCCGAAGAACGTTTTCCAATGATGAGCACT

TTTAAAGTTCTGCTATGTGGCGCGGTATTATCCCGTATTGACGCCGGGCAAGAGCAACTC

GGTCGCCGCATACACTATTCTCAGAATGACTTGGTTGAGTACTCACCAGTCACAGAAAAG

CATCTTACGGATGGCATGACAGTAAGAGAATTATGCAGTGCTGCCATAACCATGAGTGAT

AACACTGCGGCCAACTTACTTCTGACAACGATCGGAGGACCGAAGGAGCTAACCGCTTTT

TTGCACAACATGGGGGATCATGTAACTCGCCTTGATCGTTGGGAACCGGAGCTGAATGAA

GCCATACCAAACGACGAGCGTGACACCACGATGCCTGTAGCAATGGCAACAACGTTGCGC

AAACTATTAACTGGCGAACTACTTACTCTAGCTTCCCGGCAACAATTAATAGACTGGATG

GAGGCGGATAAAGTTGCAGGACCACTTCTGCGCTCGGCCCTTCCGGCTGGCTGGTTTATT

GCTGATAAATCTGGAGCCGGTGAGCGTGGGTCTCGCGGTATCATTGCAGCACTGGGGCCA

GATGGTAAGCCCTCCCGTATCGTAGTTATCTACACGACGGGGAGTCAGGCAACTATGGAT

GAACGAAATAGACAGATCGCTGAGATAGGTGCCTCACTGATTAAGCATTGGTAACTGTCA

GACCAAGTTTACTCATATATACTTTAGATTGATTTAAAACTTCATTTTTAATTTAAAAGG

ATCTAGGTGAAGATCCTTTTTGATAATCTCATGACCAAAATCCCTTAACGTGAGTTTTCG

TTCCACTGAGCGTCAGACCCCGTAGAAAAGATCAAAGGATCTTCTTGAGATCCTTTTTTT

CTGCGCGTAATCTGCTGCTTGCAAACAAAAAAACCACCGCTACCAGCGGTGGTTTGTTTG

CCGGATCAAGAGCTACCAACTCTTTTTCCGAAGGTAACTGGCTTCAGCAGAGCGCAGATA

CCAAATACTGTCCTTCTAGTGTAGCCGTAGTTAGGCCACCACTTCAAGAACTCTGTAGCA

CCGCCTACATACCTCGCTCTGCTAATCCTGTTACCAGTGGCTGCTGCCAGTGGCGATAAG

TCGTGTCTTACCGGGTTGGACTCAAGACGATAGTTACCGGATAAGGCGCAGCGGTCGGGC

TGAACGGGGGGTTCGTGCACACAGCCCAGCTTGGAGCGAACGACCTACACCGAACTGAGA

TACCTACAGCGTGAGCTATGAGAAAGCGCCACGCTTCCCGAAGGGAGAAAGGCGGACAGG

TATCCGGTAAGCGGCAGGGTCGGAACAGGAGAGCGCACGAGGGAGCTTCCAGGGGGAAAC

GCCTGGTATCTTTATAGTCCTGTCGGGTTTCGCCACCTCTGACTTGAGCGTCGATTTTTG

TGATGCTCGTCAGGGGGGCGGAGCCTATGGAAAAACGCCAGCAACGCGGCCTTTTTACGG

TTCCTGGCCTTTTGCTGGCCTTTTGCTCACATGTTCTTTCCTGCGTTATCCCCTGATTCT

GTGGATAACCGTATTACCGCCTTTGAGTGAGCTGATACCGCTCGCCGCAGCCGAACGACC

GAGCGCAGCGAGTCAGTGAGCGAGGAAGCGGAAGAGCGCCCAATACGCAAACCGCCTCTC

CCCGCGCGTTGGCCGATTCATTAATGCAGCTGGCACGACAGGTTTCCCGACTGGAAAGCG

GGCAGTGAGCGCAACGCAATTAATGTGAGTTAGCTCACTCATTAGGCACCCCAGGCTTTA

CACTTTATGCTTCCGGCTCGTATGTTGTGTGGAATTGTGAGCGGATAACAATTTCACACA

GGAAACAGCTATGACCATGATTACGCCAAGCGCGCAATTAACCCTCACTAAAGGGAACAA

AAGCTGGGTACCGGGCCCCCCCTCGAGAGTC

**SAG**

5480 bp

5’LTR CMV promoter 1-530

R region 531-589

U5 region 590-665

Packaging signal 666-1042

ADA promoter 1052-1494

eGFP gene 1508-2227

3’LTR U3 region 2298-2414

R region 2415-2482

U5 region 2483-2559

T7 primer site 2640-2609

CGTTACATAACTTACGGTAAATGGCCCGCCGGCTGACCGCCCAACGACCCCCGCCCATTG

ACGTCAATAATGACGTATGTTCCCATAGTAACGCCAATAGGGACTTTCCATTGACGTCAA

TGGGTGGAGTATTTACGGTAAACTGCCCACTTGGCAGTACATCAAGTGTATCATATGCCA

AGTACGCCCCCTATTGACGTCAATGACGGTAAATGGCCCGCCTGGCATTATGCCCAGTAC

ATGACCTTATGGGACTTTCCTACTTGGCAGTACATCTACGTATTAGTCATCGCTATTACC

ATGGTGATGCGGTTTTGGCAGTACATCAATGGGCGTGGATAGCGGTTTGACTCACGGGGA

TTTCCAAGTCTCCACCCCATTGACGTCAATGGGAGTTTGTTTTGGCACCAAAATCAACGG

GACTTTCCAAAATGTCGTAACAACTCCGCCCCATTGACGCAAATGGGCGGTAGGCGTGTA

CGGTGGGAGGTCTATATAAGCAGAGCTCGTTTAGTGAACCGCGCCAGTCTTCCGATAGAC

TGCGTCGCCCGGGTACCCGTATTCCCAATAAAGCCTCTTGCTGTTTGCATCCGAATCGTG

GTCTCGCTGTTCCTTGGGAGGGTCTCCTCTGAGTGATTGACTACCCACGACGGGGGTCTT

TCATTTGGGGGCTCGTCCGGGATTCGGAGACCCCTGCCCAGGGACCACCGACCCACCACC

GGGAGGTAAGCTGGCCACCAACTTATCTGTGTCTGTCCGATTGTCTAGTGTCTATGACTG

ATTTTATGCGCCTGCGTCGGTACTAGTTAGCTAACTAGCTCTGTATCTGGCGGACCCGTG

GTGGAACTGACGAGTTCGGAACACCCGGCCGCAACCCTGGGAGACGTCCCAGGGACTTCG

GGGGCCGTTTTTGTGGCCCGACCTGAGTCCAAAAATCCCGATCGTTTTGGACTCTTTGGT

GCACCCCCCTTAGGAGGAGGGATATGTGGTTCTGGTAGGAGACGAGAACCTAAAACAGTT

CCCGCCTCCGTCTGAATTTTTGCTAAGCTTCCTCGAGGCTTGCGATGCTCCCGGGGTCCC

CGCTTCTAGCTTGGGCCTGGCGCACAGCAGCGCCCAGACTGCAGGGGGACGCTTGAAAGT

TGCTGGAGGAGCCGGGGGGAAGGCAGCGCCCAGCGAGGCGGCTGGAGCGCGCGCCCACAG

GTGGGTCCGGTCGGGCGCCGCGGGGCCGTAGTTTTCGGGTCGGCGGGCGAGGACGCCGGG

TCCAGAATTCCAGGAAATGCGCGATCCAGGCCGGCGGGCGGGGCGGGGGCTCCGGCGAGA

GGGCGGGCCCCGGGAACGGCGGCGGGCGGGGCGGGAGGCGGGGCCCGGCCCGTTAAGAAG

AGCGTGGCCGGCCGCGGCCACCGCTGGCCCCAGGGAAAGCCGAGCGGCCACCGAGCCGGC

AGAGACCCACCGAGCGGCGGCGGAGGGAGCAGCGCCGGGGCGCACGAGGGCACCACCGGT

CGCCACCATGGTGAGCAAGGGCGAGGAGCTGTTCACCGGGGTGGTGCCCATCCTGGTCGA

GCTGGACGGCGACGTAAACGGCCACAAGTTCAGCGTGTCCGGCGAGGGCGAGGGCGATGC

CACCTACGGCAAGCTGACCCTGAAGTTCATCTGCACCACCGGCAAGCTGCCCGTGCCCTG

GCCCACCCTCGTGACCACCCTGACCTACGGCGTGCAGTGCTTCAGCCGCTACCCCGACCA

CATGAAGCAGCACGACTTCTTCAAGTCCGCCATGCCCGAAGGCTACGTCCAGGAGCGCAC

CATCTTCTTCAAGGACGACGGCAACTACAAGACCCGCGCCGAGGTGAAGTTCGAGGGCGA

CACCCTGGTGAACCGCATCGAGCTGAAGGGCATCGACTTCAAGGAGGACGGCAACATCCT

GGGGCACAAGCTGGAGTACAACTACAACAGCCACAACGTCTATATCATGGCCGACAAGCA

GAAGAACGGCATCAAGGTGAACTTCAAGATCCGCCACAACATCGAGGACGGCAGCGTGCA

GCTCGCCGACCACTACCAGCAGAACACCCCCATCGGCGACGGCCCCGTGCTGCTGCCCGA

CAACCACTACCTGAGCACCCAGTCCGCCCTGAGCAAAGACCCCAACGAGAAGCGCGATCA

CATGGTCCTGCTGGAGTTCGTGACCGCCGCCGGGATCACTCTCGGCATGGACGAGCTGTA

CAAGTAAAGCGGCCGCGACTCTAGAGTCGACCATAGATAAAATAAAAGATTTTATTTAGT

CTCCAGAAAAAGGGGGGAATGAAAGACCCCACCTGTAGGTTTGGCAAGCTAGCGATATCA

GTTCGCTTCTCGCTTCTGTTCGCGCGCTTCTGCTCCCCGAGCTCAATAAAAGAGCCCACA

ACCCCTCACTCGGGGCGCCAGTCCTCCGATTGACTGAGTCGCCCGGGTACCCGTGTATCC

AATAAACCCTCTTGCAGTTGCATCCGACTTGTGGTCTCGCTGTTCCTTGGGAGGGTCTCC

TCTGAGTGATTGACTACCCGTCAGCGGGGGTCTTTCATTTGGGGGCTCGTCCGGGATCGC

GGCCGCCACCGCGGTGGAGCTCCAATTCGCCCTATAGTGAGTCGTATTACGCGCGCTCAC

TGGCCGTCGTTTTACAACGTCGTGACTGGGAAAACCCTGGCGTTACCCAACTTAATCGCC

TTGCAGCACATCCCCCTTTCGCCAGCTGGCGTAATAGCGAAGAGGCCCGCACCGATCGCC

CTTCCCAACAGTTGCGCAGCCTGAATGGCGAATGGGACGCGCCCTGTAGCGGCGCATTAA

GCGCGGCGGGTGTGGTGGTTACGCGCAGCGTGACCGCTACACTTGCCAGCGCCCTAGCGC

CCGCTCCTTTCGCTTTCTTCCCTTCCTTTCTCGCCACGTTCGCCGGCTTTCCCCGTCAAG

CTCTAAATCGGGGGCTCCCTTTAGGGTTCCGATTTAGTGCTTTACGGCACCTCGACCCCA

AAAAACTTGATTAGGGTGATGGTTCACGTAGTGGGCCATCGCCCTGATAGACGGTTTTTC

GCCCTTTGACGTTGGAGTCCACGTTCTTTAATAGTGGACTCTTGTTCCAAACTGGAACAA

CACTCAACCCTATCTCGGTCTATTCTTTTGATTTATAAGGGATTTTGCCGATTTCGGCCT

ATTGGTTAAAAAATGAGCTGATTTAACAAAAATTTAACGCGAATTTTAACAAAATATTAA

CGCTTACAATTTAGGTGGCACTTTTCGGGGAAATGTGCGCGGAACCCCTATTTGTTTATT

TTTCTAAATACATTCAAATATGTATCCGCTCATGAGACAATAACCCTGATAAATGCTTCA

ATAATATTGAAAAAGGAAGAGTATGAGTATTCAACATTTCCGTGTCGCCCTTATTCCCTT

TTTTGCGGCATTTTGCCTTCCTGTTTTTGCTCACCCAGAAACGCTGGTGAAAGTAAAAGA

TGCTGAAGATCAGTTGGGTGCACGAGTGGGTTACATCGAACTGGATCTCAACAGCGGTAA

GATCCTTGAGAGTTTTCGCCCCGAAGAACGTTTTCCAATGATGAGCACTTTTAAAGTTCT

GCTATGTGGCGCGGTATTATCCCGTATTGACGCCGGGCAAGAGCAACTCGGTCGCCGCAT

ACACTATTCTCAGAATGACTTGGTTGAGTACTCACCAGTCACAGAAAAGCATCTTACGGA

TGGCATGACAGTAAGAGAATTATGCAGTGCTGCCATAACCATGAGTGATAACACTGCGGC

CAACTTACTTCTGACAACGATCGGAGGACCGAAGGAGCTAACCGCTTTTTTGCACAACAT

GGGGGATCATGTAACTCGCCTTGATCGTTGGGAACCGGAGCTGAATGAAGCCATACCAAA

CGACGAGCGTGACACCACGATGCCTGTAGCAATGGCAACAACGTTGCGCAAACTATTAAC

TGGCGAACTACTTACTCTAGCTTCCCGGCAACAATTAATAGACTGGATGGAGGCGGATAA

AGTTGCAGGACCACTTCTGCGCTCGGCCCTTCCGGCTGGCTGGTTTATTGCTGATAAATC

TGGAGCCGGTGAGCGTGGGTCTCGCGGTATCATTGCAGCACTGGGGCCAGATGGTAAGCC

CTCCCGTATCGTAGTTATCTACACGACGGGGAGTCAGGCAACTATGGATGAACGAAATAG

ACAGATCGCTGAGATAGGTGCCTCACTGATTAAGCATTGGTAACTGTCAGACCAAGTTTA

CTCATATATACTTTAGATTGATTTAAAACTTCATTTTTAATTTAAAAGGATCTAGGTGAA

GATCCTTTTTGATAATCTCATGACCAAAATCCCTTAACGTGAGTTTTCGTTCCACTGAGC

GTCAGACCCCGTAGAAAAGATCAAAGGATCTTCTTGAGATCCTTTTTTTCTGCGCGTAAT

CTGCTGCTTGCAAACAAAAAAACCACCGCTACCAGCGGTGGTTTGTTTGCCGGATCAAGA

GCTACCAACTCTTTTTCCGAAGGTAACTGGCTTCAGCAGAGCGCAGATACCAAATACTGT

CCTTCTAGTGTAGCCGTAGTTAGGCCACCACTTCAAGAACTCTGTAGCACCGCCTACATA

CCTCGCTCTGCTAATCCTGTTACCAGTGGCTGCTGCCAGTGGCGATAAGTCGTGTCTTAC

CGGGTTGGACTCAAGACGATAGTTACCGGATAAGGCGCAGCGGTCGGGCTGAACGGGGGG

TTCGTGCACACAGCCCAGCTTGGAGCGAACGACCTACACCGAACTGAGATACCTACAGCG

TGAGCTATGAGAAAGCGCCACGCTTCCCGAAGGGAGAAAGGCGGACAGGTATCCGGTAAG

CGGCAGGGTCGGAACAGGAGAGCGCACGAGGGAGCTTCCAGGGGGAAACGCCTGGTATCT

TTATAGTCCTGTCGGGTTTCGCCACCTCTGACTTGAGCGTCGATTTTTGTGATGCTCGTC

AGGGGGGCGGAGCCTATGGAAAAACGCCAGCAACGCGGCCTTTTTACGGTTCCTGGCCTT

TTGCTGGCCTTTTGCTCACATGTTCTTTCCTGCGTTATCCCCTGATTCTGTGGATAACCG

TATTACCGCCTTTGAGTGAGCTGATACCGCTCGCCGCAGCCGAACGACCGAGCGCAGCGA

GTCAGTGAGCGAGGAAGCGGAAGAGCGCCCAATACGCAAACCGCCTCTCCCCGCGCGTTG

GCCGATTCATTAATGCAGCTGGCACGACAGGTTTCCCGACTGGAAAGCGGGCAGTGAGCG

CAACGCAATTAATGTGAGTTAGCTCACTCATTAGGCACCCCAGGCTTTACACTTTATGCT

TCCGGCTCGTATGTTGTGTGGAATTGTGAGCGGATAACAATTTCACACAGGAAACAGCTA

TGACCATGATTACGCCAAGCGCGCAATTAACCCTCACTAAAGGGAACAAAAGCTGGGTAC

CGGGCCCCCCCTCGAGAGTC

**SLAG**

7767 bp

5’LTR CMV promoter 1-530

R region 531-589

U3 region 590-665

Packaging signal 666-1042

ADA LCR 1058-3315

ADA promoter 3339-3781

eGFP gene 3795-4514

3’LTR DU3 region 4585-4702

R region 4703-4769

U5 region 4770-4846

T7 primer site 4927-4896

CGTTACATAACTTACGGTAAATGGCCCGCCGGCTGACCGCCCAACGACCCCCGCCCATTG

ACGTCAATAATGACGTATGTTCCCATAGTAACGCCAATAGGGACTTTCCATTGACGTCAA

TGGGTGGAGTATTTACGGTAAACTGCCCACTTGGCAGTACATCAAGTGTATCATATGCCA

AGTACGCCCCCTATTGACGTCAATGACGGTAAATGGCCCGCCTGGCATTATGCCCAGTAC

ATGACCTTATGGGACTTTCCTACTTGGCAGTACATCTACGTATTAGTCATCGCTATTACC

ATGGTGATGCGGTTTTGGCAGTACATCAATGGGCGTGGATAGCGGTTTGACTCACGGGGA

TTTCCAAGTCTCCACCCCATTGACGTCAATGGGAGTTTGTTTTGGCACCAAAATCAACGG

GACTTTCCAAAATGTCGTAACAACTCCGCCCCATTGACGCAAATGGGCGGTAGGCGTGTA

CGGTGGGAGGTCTATATAAGCAGAGCTCGTTTAGTGAACCGCGCCAGTCTTCCGATAGAC

TGCGTCGCCCGGGTACCCGTATTCCCAATAAAGCCTCTTGCTGTTTGCATCCGAATCGTG

GTCTCGCTGTTCCTTGGGAGGGTCTCCTCTGAGTGATTGACTACCCACGACGGGGGTCTT

TCATTTGGGGGCTCGTCCGGGATTCGGAGACCCCTGCCCAGGGACCACCGACCCACCACC

GGGAGGTAAGCTGGCCACCAACTTATCTGTGTCTGTCCGATTGTCTAGTGTCTATGACTG

ATTTTATGCGCCTGCGTCGGTACTAGTTAGCTAACTAGCTCTGTATCTGGCGGACCCGTG

GTGGAACTGACGAGTTCGGAACACCCGGCCGCAACCCTGGGAGACGTCCCAGGGACTTCG

GGGGCCGTTTTTGTGGCCCGACCTGAGTCCAAAAATCCCGATCGTTTTGGACTCTTTGGT

GCACCCCCCTTAGGAGGAGGGATATGTGGTTCTGGTAGGAGACGAGAACCTAAAACAGTT

CCCGCCTCCGTCTGAATTTTTGCTAAGCTCTAGAGGAGCATGCACCACCATGCCCGGCTA

ATTTTTTGTATTTTTAGTAGAGACGGGGTTTCTCCTTGTTGGCCAGGCTGGTCTCGAACT

CCCGACTTCAGGTGATCCGCCCACCTCAGCCTCCCAAAGTGCCGGGATTACAGGCATGAG

CCACCGCACCCGGCCAAGGGACAGCAGTTTCTAAACTGTCCCTCTCTGATGCAGAGGGGA

ATTGGGGCTAAATCAGCAATGTGCCTTTTCTGTCTCATATTTGAATGTCTACTCTGCACG

AGGCGCTGTCCTGCTTTGCATACAGTGACTCATTTAATGTTTATGTCAGCCCTCTGAGGA

AGGTCCTGTCCTATTATTAACTTCACTTATTATGAGGAAACTGAGACTCAGAGAGGGGAG

GGAACTTGCCAAAGTCACACAGCTGGCAAGCAGCAGAGCTAGACTTGAACCCAGATCTGC

CTGCACTCAAGTAGAAGCTGTTCATTGCTTTGCTCATTTGCCAATTCCACTTTATGCAAA

AAAGAGGGGGCAGTGTGGGGGGAAGAGTTAGAATCAGGGTGGCAGGGTGGGCCAGTGCAT

TAGCCCTGGGCTTCAGATGTACTGGGGTTGAATTCCTGCCTGCCGCTTAGCAGCTAGGGT

ACCTCAGGTAGACAACTCCTGAAACTCAGCTTCCCCCTCTGTAAAATGGGGTGACAAAAC

CAAGATCTTGGGGTTCTTGGGGAAACTGACATGCTGATTGGTTTTTGTACAGTGCCTGGC

TGGTAACAGCAGGCCCTCAGGGGTGCGTTTCCTTCCTGGGGACTGGAGTGGGGGTTGCAG

TAGACTCTGGGAGGCCTCTCCAGCTGCAGAATCTCCCTCCTCCCTCCTCCTTTTTGTCTT

CCTGACACAAAACCCACCAGCTGCACTTCTTTGGGCTTGCAGTGGCTTTCAGTTACCAGA

GCCACCTGTTAAAACAAAAATGTGCCTAGGAAGAGCCTGCCTTACCCATTTTGACTCACA

TGGCAGTTGGTGGTGGAGGGGAACAAAGGAGACTGAGTTTCATCGAAGCCTTTTGCTTCG

GAGGAGGAAGGGAGGATCAGAGAGAGGAAGTGGTCTGTGTTCACACAGGGAGGCAGGGGA

GGCCAGGCAGCTTCCCAATCCTGCATTCAACCTCAGGGTGGGCTTGACCTGGGTGGCTGG

GGGCCCTGTGATCCAGGAGAGACTTGTCCACCTGCTCAGGTGTCTTGAAGGGGTCCCTGT

GGTACCCCCTGGGCGGGGCAAGGTAGTAGGACCATGGTCTGGCTGGGGAGGTGGAGAGGA

GCAGGCTGTGGGCGCAGAGTGAGGTTGGAATCTGTATTTACCCAAGGTGTTGGGGGTAGG

CTTGCCCTCAGCCCTTAATGTTCTCAGGCCCCTGAGCAGTTGTGGGGGATAACCTCTGCA

CTCCTAGTGACCAGGGAGCTAGAACAGCAAGGAATTTGAACTTGGACACCAGCTGGGGTC

AGGCTCTCTGGGTCTGAGTCCTGATTTCCCACTTTCCAGCTAGAGGAGCTTGAATGAGTC

ATTTAACTTCACGGTGCCTCAGTTTCCCCTCTCTAAAATGAGAATTATACCCATACCCAC

CTCTCAAACACCAAGTGCAGGCCTGGCTCAGAGCAGGTGCTGCAGCAATAGCTGCCATTG

GTCAGCATCATCATCATGGTTGGTAATGGTCCTACTTTGACTTTTGAGACAGAGTCTCAC

TCTGTCGCCCAGGCTGGAGTGCAGTGGTGCAATCTCGGCTCACTACAACCTCTGCTCCCG

GGTTCAAGTGATTCTTCTGCCTCAGTCTCCCAAGTAGTTGGGATTACAGGTGTGCGCCAC

CATGCCTGGCTAATTTTTGTGTTTTTAGTAGAGACAGGGTTTCACCATGTTGGCCATAAC

AATGGCTGTCCTTGAAGTAGTCCTGGGTACCAGGTGCCTTCAGTGACTTTTTTTTTTTTT

TTTTTTTTGAGCTGGAGTCTTCCTCTGTCACCCAAGCTGGAGTGCAGTGGCACGATTTTG

GCTCACTGCAACCTCTGCCTCCTGGGTTCATGCGATCCTCCTGCCTCAGCCTCCCAAGTA

GCTGGGACTTGGGATACACTTGCCCCCGCTGGTCCTCCCTTCCACCTCTGTGAAGAGGAG

GTCTCAAACTCCTGGCCTCAAGTGATCCACCCACCTCAGCCTCCCAAAGTGCTGGGATTT

CAAGAGTGAGCCACCGCACCTGGCCCCTGTTTAGATGTTAGCATCAGTGACCCAGCACCT

TGCTATGTGGCATGCCTGCAGGTCGACTCTAGAGCTTCCTCGAGGCTTGCGATGCTCCCG

GGGTCCCCGCTTCTAGCTTGGGCCTGGCGCACAGCAGCGCCCAGACTGCAGGGGGACGCT

TGAAAGTTGCTGGAGGAGCCGGGGGGAAGGCAGCGCCCAGCGAGGCGGCTGGAGCGCGCG

CCCACAGGTGGGTCCGGTCGGGCGCCGCGGGGCCGTAGTTTTCGGGTCGGCGGGCGAGGA

CGCCGGGTCCAGAATTCCAGGAAATGCGCGATCCAGGCCGGCGGGCGGGGCGGGGGCTCC

GGCGAGAGGGCGGGCCCCGGGAACGGCGGCGGGCGGGGCGGGAGGCGGGGCCCGGCCCGT

TAAGAAGAGCGTGGCCGGCCGCGGCCACCGCTGGCCCCAGGGAAAGCCGAGCGGCCACCG

AGCCGGCAGAGACCCACCGAGCGGCGGCGGAGGGAGCAGCGCCGGGGCGCACGAGGGCAC

CACCGGTCGCCACCATGGTGAGCAAGGGCGAGGAGCTGTTCACCGGGGTGGTGCCCATCC

TGGTCGAGCTGGACGGCGACGTAAACGGCCACAAGTTCAGCGTGTCCGGCGAGGGCGAGG

GCGATGCCACCTACGGCAAGCTGACCCTGAAGTTCATCTGCACCACCGGCAAGCTGCCCG

TGCCCTGGCCCACCCTCGTGACCACCCTGACCTACGGCGTGCAGTGCTTCAGCCGCTACC

CCGACCACATGAAGCAGCACGACTTCTTCAAGTCCGCCATGCCCGAAGGCTACGTCCAGG

AGCGCACCATCTTCTTCAAGGACGACGGCAACTACAAGACCCGCGCCGAGGTGAAGTTCG

AGGGCGACACCCTGGTGAACCGCATCGAGCTGAAGGGCATCGACTTCAAGGAGGACGGCA

ACATCCTGGGGCACAAGCTGGAGTACAACTACAACAGCCACAACGTCTATATCATGGCCG

ACAAGCAGAAGAACGGCATCAAGGTGAACTTCAAGATCCGCCACAACATCGAGGACGGCA

GCGTGCAGCTCGCCGACCACTACCAGCAGAACACCCCCATCGGCGACGGCCCCGTGCTGC

TGCCCGACAACCACTACCTGAGCACCCAGTCCGCCCTGAGCAAAGACCCCAACGAGAAGC

GCGATCACATGGTCCTGCTGGAGTTCGTGACCGCCGCCGGGATCACTCTCGGCATGGACG

AGCTGTACAAGTAAAGCGGCCGCGACTCTAGAGTCGACCATAGATAAAATAAAAGATTTT

ATTTAGTCTCCAGAAAAAGGGGGGAATGAAAGACCCCACCTGTAGGTTTGGCAAGCTAGC

GATATCAGTTCGCTTCTCGCTTCTGTTCGCGCGCTTCTGCTCCCCGAGCTCAATAAAAGA

GCCCACAACCCCTCACTCGGGGCGCCAGTCCTCCGATTGACTGAGTCGCCCGGGTACCCG

TGTATCCAATAAACCCTCTTGCAGTTGCATCCGACTTGTGGTCTCGCTGTTCCTTGGGAG

GGTCTCCTCTGAGTGATTGACTACCCGTCAGCGGGGGTCTTTCATTTGGGGGCTCGTCCG

GGATCGCGGCCGCCACCGCGGTGGAGCTCCAATTCGCCCTATAGTGAGTCGTATTACGCG

CGCTCACTGGCCGTCGTTTTACAACGTCGTGACTGGGAAAACCCTGGCGTTACCCAACTT

AATCGCCTTGCAGCACATCCCCCTTTCGCCAGCTGGCGTAATAGCGAAGAGGCCCGCACC

GATCGCCCTTCCCAACAGTTGCGCAGCCTGAATGGCGAATGGGACGCGCCCTGTAGCGGC

GCATTAAGCGCGGCGGGTGTGGTGGTTACGCGCAGCGTGACCGCTACACTTGCCAGCGCC

CTAGCGCCCGCTCCTTTCGCTTTCTTCCCTTCCTTTCTCGCCACGTTCGCCGGCTTTCCC

CGTCAAGCTCTAAATCGGGGGCTCCCTTTAGGGTTCCGATTTAGTGCTTTACGGCACCTC

GACCCCAAAAAACTTGATTAGGGTGATGGTTCACGTAGTGGGCCATCGCCCTGATAGACG

GTTTTTCGCCCTTTGACGTTGGAGTCCACGTTCTTTAATAGTGGACTCTTGTTCCAAACT

GGAACAACACTCAACCCTATCTCGGTCTATTCTTTTGATTTATAAGGGATTTTGCCGATT

TCGGCCTATTGGTTAAAAAATGAGCTGATTTAACAAAAATTTAACGCGAATTTTAACAAA

ATATTAACGCTTACAATTTAGGTGGCACTTTTCGGGGAAATGTGCGCGGAACCCCTATTT

GTTTATTTTTCTAAATACATTCAAATATGTATCCGCTCATGAGACAATAACCCTGATAAA

TGCTTCAATAATATTGAAAAAGGAAGAGTATGAGTATTCAACATTTCCGTGTCGCCCTTA

TTCCCTTTTTTGCGGCATTTTGCCTTCCTGTTTTTGCTCACCCAGAAACGCTGGTGAAAG

TAAAAGATGCTGAAGATCAGTTGGGTGCACGAGTGGGTTACATCGAACTGGATCTCAACA

GCGGTAAGATCCTTGAGAGTTTTCGCCCCGAAGAACGTTTTCCAATGATGAGCACTTTTA

AAGTTCTGCTATGTGGCGCGGTATTATCCCGTATTGACGCCGGGCAAGAGCAACTCGGTC

GCCGCATACACTATTCTCAGAATGACTTGGTTGAGTACTCACCAGTCACAGAAAAGCATC

TTACGGATGGCATGACAGTAAGAGAATTATGCAGTGCTGCCATAACCATGAGTGATAACA

CTGCGGCCAACTTACTTCTGACAACGATCGGAGGACCGAAGGAGCTAACCGCTTTTTTGC

ACAACATGGGGGATCATGTAACTCGCCTTGATCGTTGGGAACCGGAGCTGAATGAAGCCA

TACCAAACGACGAGCGTGACACCACGATGCCTGTAGCAATGGCAACAACGTTGCGCAAAC

TATTAACTGGCGAACTACTTACTCTAGCTTCCCGGCAACAATTAATAGACTGGATGGAGG

CGGATAAAGTTGCAGGACCACTTCTGCGCTCGGCCCTTCCGGCTGGCTGGTTTATTGCTG

ATAAATCTGGAGCCGGTGAGCGTGGGTCTCGCGGTATCATTGCAGCACTGGGGCCAGATG

GTAAGCCCTCCCGTATCGTAGTTATCTACACGACGGGGAGTCAGGCAACTATGGATGAAC

GAAATAGACAGATCGCTGAGATAGGTGCCTCACTGATTAAGCATTGGTAACTGTCAGACC

AAGTTTACTCATATATACTTTAGATTGATTTAAAACTTCATTTTTAATTTAAAAGGATCT

AGGTGAAGATCCTTTTTGATAATCTCATGACCAAAATCCCTTAACGTGAGTTTTCGTTCC

ACTGAGCGTCAGACCCCGTAGAAAAGATCAAAGGATCTTCTTGAGATCCTTTTTTTCTGC

GCGTAATCTGCTGCTTGCAAACAAAAAAACCACCGCTACCAGCGGTGGTTTGTTTGCCGG

ATCAAGAGCTACCAACTCTTTTTCCGAAGGTAACTGGCTTCAGCAGAGCGCAGATACCAA

ATACTGTCCTTCTAGTGTAGCCGTAGTTAGGCCACCACTTCAAGAACTCTGTAGCACCGC

CTACATACCTCGCTCTGCTAATCCTGTTACCAGTGGCTGCTGCCAGTGGCGATAAGTCGT

GTCTTACCGGGTTGGACTCAAGACGATAGTTACCGGATAAGGCGCAGCGGTCGGGCTGAA

CGGGGGGTTCGTGCACACAGCCCAGCTTGGAGCGAACGACCTACACCGAACTGAGATACC

TACAGCGTGAGCTATGAGAAAGCGCCACGCTTCCCGAAGGGAGAAAGGCGGACAGGTATC

CGGTAAGCGGCAGGGTCGGAACAGGAGAGCGCACGAGGGAGCTTCCAGGGGGAAACGCCT

GGTATCTTTATAGTCCTGTCGGGTTTCGCCACCTCTGACTTGAGCGTCGATTTTTGTGAT

GCTCGTCAGGGGGGCGGAGCCTATGGAAAAACGCCAGCAACGCGGCCTTTTTACGGTTCC

TGGCCTTTTGCTGGCCTTTTGCTCACATGTTCTTTCCTGCGTTATCCCCTGATTCTGTGG

ATAACCGTATTACCGCCTTTGAGTGAGCTGATACCGCTCGCCGCAGCCGAACGACCGAGC

GCAGCGAGTCAGTGAGCGAGGAAGCGGAAGAGCGCCCAATACGCAAACCGCCTCTCCCCG

CGCGTTGGCCGATTCATTAATGCAGCTGGCACGACAGGTTTCCCGACTGGAAAGCGGGCA

GTGAGCGCAACGCAATTAATGTGAGTTAGCTCACTCATTAGGCACCCCAGGCTTTACACT

TTATGCTTCCGGCTCGTATGTTGTGTGGAATTGTGAGCGGATAACAATTTCACACAGGAA

ACAGCTATGACCATGATTACGCCAAGCGCGCAATTAACCCTCACTAAAGGGAACAAAAGC

TGGGTACCGGGCCCCCCCTCGAGAGTC

**SiLAG**

7767 bp

5’LTR CMV promoter 1-530

R region 531-589

U3 region 590-665

Packaging signal 666-1042

ADA LCR 3324-1067 (reverse orientation)

ADA promoter 3339-3781

eGFP gene 3795-4514

3’LTR DU3 region 4585-4702

R region 4703-4769

U5 region 4770-4846

T7 primer site 4927-4896

CGTTACATAACTTACGGTAAATGGCCCGCCGGCTGACCGCCCAACGACCCCCGCCCATTG

ACGTCAATAATGACGTATGTTCCCATAGTAACGCCAATAGGGACTTTCCATTGACGTCAA

TGGGTGGAGTATTTACGGTAAACTGCCCACTTGGCAGTACATCAAGTGTATCATATGCCA

AGTACGCCCCCTATTGACGTCAATGACGGTAAATGGCCCGCCTGGCATTATGCCCAGTAC

ATGACCTTATGGGACTTTCCTACTTGGCAGTACATCTACGTATTAGTCATCGCTATTACC

ATGGTGATGCGGTTTTGGCAGTACATCAATGGGCGTGGATAGCGGTTTGACTCACGGGGA

TTTCCAAGTCTCCACCCCATTGACGTCAATGGGAGTTTGTTTTGGCACCAAAATCAACGG

GACTTTCCAAAATGTCGTAACAACTCCGCCCCATTGACGCAAATGGGCGGTAGGCGTGTA

CGGTGGGAGGTCTATATAAGCAGAGCTCGTTTAGTGAACCGCGCCAGTCTTCCGATAGAC

TGCGTCGCCCGGGTACCCGTATTCCCAATAAAGCCTCTTGCTGTTTGCATCCGAATCGTG

GTCTCGCTGTTCCTTGGGAGGGTCTCCTCTGAGTGATTGACTACCCACGACGGGGGTCTT

TCATTTGGGGGCTCGTCCGGGATTCGGAGACCCCTGCCCAGGGACCACCGACCCACCACC

GGGAGGTAAGCTGGCCACCAACTTATCTGTGTCTGTCCGATTGTCTAGTGTCTATGACTG

ATTTTATGCGCCTGCGTCGGTACTAGTTAGCTAACTAGCTCTGTATCTGGCGGACCCGTG

GTGGAACTGACGAGTTCGGAACACCCGGCCGCAACCCTGGGAGACGTCCCAGGGACTTCG

GGGGCCGTTTTTGTGGCCCGACCTGAGTCCAAAAATCCCGATCGTTTTGGACTCTTTGGT

GCACCCCCCTTAGGAGGAGGGATATGTGGTTCTGGTAGGAGACGAGAACCTAAAACAGTT

CCCGCCTCCGTCTGAATTTTTGCTAAGCTCTAGAGTCGACCTGCAGGCATGCCACATAGC

AAGGTGCTGGGTCACTGATGCTAACATCTAAACAGGGGCCAGGTGCGGTGGCTCACTCTT

GAAATCCCAGCACTTTGGGAGGCTGAGGTGGGTGGATCACTTGAGGCCAGGAGTTTGAGA

CCTCCTCTTCACAGAGGTGGAAGGGAGGACCAGCGGGGGCAAGTGTATCCCAAGTCCCAG

CTACTTGGGAGGCTGAGGCAGGAGGATCGCATGAACCCAGGAGGCAGAGGTTGCAGTGAG

CCAAAATCGTGCCACTGCACTCCAGCTTGGGTGACAGAGGAAGACTCCAGCTCAAAAAAA

AAAAAAAAAAAAAAGTCACTGAAGGCACCTGGTACCCAGGACTACTTCAAGGACAGCCAT

TGTTATGGCCAACATGGTGAAACCCTGTCTCTACTAAAAACACAAAAATTAGCCAGGCAT

GGTGGCGCACACCTGTAATCCCAACTACTTGGGAGACTGAGGCAGAAGAATCACTTGAAC

CCGGGAGCAGAGGTTGTAGTGAGCCGAGATTGCACCACTGCACTCCAGCCTGGGCGACAG

AGTGAGACTCTGTCTCAAAAGTCAAAGTAGGACCATTACCAACCATGATGATGATGCTGA

CCAATGGCAGCTATTGCTGCAGCACCTGCTCTGAGCCAGGCCTGCACTTGGTGTTTGAGA

GGTGGGTATGGGTATAATTCTCATTTTAGAGAGGGGAAACTGAGGCACCGTGAAGTTAAA

TGACTCATTCAAGCTCCTCTAGCTGGAAAGTGGGAAATCAGGACTCAGACCCAGAGAGCC

TGACCCCAGCTGGTGTCCAAGTTCAAATTCCTTGCTGTTCTAGCTCCCTGGTCACTAGGA

GTGCAGAGGTTATCCCCCACAACTGCTCAGGGGCCTGAGAACATTAAGGGCTGAGGGCAA

GCCTACCCCCAACACCTTGGGTAAATACAGATTCCAACCTCACTCTGCGCCCACAGCCTG

CTCCTCTCCACCTCCCCAGCCAGACCATGGTCCTACTACCTTGCCCCGCCCAGGGGGTAC

CACAGGGACCCCTTCAAGACACCTGAGCAGGTGGACAAGTCTCTCCTGGATCACAGGGCC

CCCAGCCACCCAGGTCAAGCCCACCCTGAGGTTGAATGCAGGATTGGGAAGCTGCCTGGC

CTCCCCTGCCTCCCTGTGTGAACACAGACCACTTCCTCTCTCTGATCCTCCCTTCCTCCT

CCGAAGCAAAAGGCTTCGATGAAACTCAGTCTCCTTTGTTCCCCTCCACCACCAACTGCC

ATGTGAGTCAAAATGGGTAAGGCAGGCTCTTCCTAGGCACATTTTTGTTTTAACAGGTGG

CTCTGGTAACTGAAAGCCACTGCAAGCCCAAAGAAGTGCAGCTGGTGGGTTTTGTGTCAG

GAAGACAAAAAGGAGGAGGGAGGAGGGAGATTCTGCAGCTGGAGAGGCCTCCCAGAGTCT

ACTGCAACCCCCACTCCAGTCCCCAGGAAGGAAACGCACCCCTGAGGGCCTGCTGTTACC

AGCCAGGCACTGTACAAAAACCAATCAGCATGTCAGTTTCCCCAAGAACCCCAAGATCTT

GGTTTTGTCACCCCATTTTACAGAGGGGGAAGCTGAGTTTCAGGAGTTGTCTACCTGAGG

TACCCTAGCTGCTAAGCGGCAGGCAGGAATTCAACCCCAGTACATCTGAAGCCCAGGGCT

AATGCACTGGCCCACCCTGCCACCCTGATTCTAACTCTTCCCCCCACACTGCCCCCTCTT

TTTTGCATAAAGTGGAATTGGCAAATGAGCAAAGCAATGAACAGCTTCTACTTGAGTGCA

GGCAGATCTGGGTTCAAGTCTAGCTCTGCTGCTTGCCAGCTGTGTGACTTTGGCAAGTTC

CCTCCCCTCTCTGAGTCTCAGTTTCCTCATAATAAGTGAAGTTAATAATAGGACAGGACC

TTCCTCAGAGGGCTGACATAAACATTAAATGAGTCACTGTATGCAAAGCAGGACAGCGCC

TCGTGCAGAGTAGACATTCAAATATGAGACAGAAAAGGCACATTGCTGATTTAGCCCCAA

TTCCCCTCTGCATCAGAGAGGGACAGTTTAGAAACTGCTGTCCCTTGGCCGGGTGCGGTG

GCTCATGCCTGTAATCCCGGCACTTTGGGAGGCTGAGGTGGGCGGATCACCTGAAGTCGG

GAGTTCGAGACCAGCCTGGCCAACAAGGAGAAACCCCGTCTCTACTAAAAATACAAAAAA

TTAGCCGGGCATGGTGGTGCATGCTCCTCTAGAGCTTCCTCGAGGCTTGCGATGCTCCCG

GGGTCCCCGCTTCTAGCTTGGGCCTGGCGCACAGCAGCGCCCAGACTGCAGGGGGACGCT

TGAAAGTTGCTGGAGGAGCCGGGGGGAAGGCAGCGCCCAGCGAGGCGGCTGGAGCGCGCG

CCCACAGGTGGGTCCGGTCGGGCGCCGCGGGGCCGTAGTTTTCGGGTCGGCGGGCGAGGA

CGCCGGGTCCAGAATTCCAGGAAATGCGCGATCCAGGCCGGCGGGCGGGGCGGGGGCTCC

GGCGAGAGGGCGGGCCCCGGGAACGGCGGCGGGCGGGGCGGGAGGCGGGGCCCGGCCCGT

TAAGAAGAGCGTGGCCGGCCGCGGCCACCGCTGGCCCCAGGGAAAGCCGAGCGGCCACCG

AGCCGGCAGAGACCCACCGAGCGGCGGCGGAGGGAGCAGCGCCGGGGCGCACGAGGGCAC

CACCGGTCGCCACCATGGTGAGCAAGGGCGAGGAGCTGTTCACCGGGGTGGTGCCCATCC

TGGTCGAGCTGGACGGCGACGTAAACGGCCACAAGTTCAGCGTGTCCGGCGAGGGCGAGG

GCGATGCCACCTACGGCAAGCTGACCCTGAAGTTCATCTGCACCACCGGCAAGCTGCCCG

TGCCCTGGCCCACCCTCGTGACCACCCTGACCTACGGCGTGCAGTGCTTCAGCCGCTACC

CCGACCACATGAAGCAGCACGACTTCTTCAAGTCCGCCATGCCCGAAGGCTACGTCCAGG

AGCGCACCATCTTCTTCAAGGACGACGGCAACTACAAGACCCGCGCCGAGGTGAAGTTCG

AGGGCGACACCCTGGTGAACCGCATCGAGCTGAAGGGCATCGACTTCAAGGAGGACGGCA

ACATCCTGGGGCACAAGCTGGAGTACAACTACAACAGCCACAACGTCTATATCATGGCCG

ACAAGCAGAAGAACGGCATCAAGGTGAACTTCAAGATCCGCCACAACATCGAGGACGGCA

GCGTGCAGCTCGCCGACCACTACCAGCAGAACACCCCCATCGGCGACGGCCCCGTGCTGC

TGCCCGACAACCACTACCTGAGCACCCAGTCCGCCCTGAGCAAAGACCCCAACGAGAAGC

GCGATCACATGGTCCTGCTGGAGTTCGTGACCGCCGCCGGGATCACTCTCGGCATGGACG

AGCTGTACAAGTAAAGCGGCCGCGACTCTAGAGTCGACCATAGATAAAATAAAAGATTTT

ATTTAGTCTCCAGAAAAAGGGGGGAATGAAAGACCCCACCTGTAGGTTTGGCAAGCTAGC

GATATCAGTTCGCTTCTCGCTTCTGTTCGCGCGCTTCTGCTCCCCGAGCTCAATAAAAGA

GCCCACAACCCCTCACTCGGGGCGCCAGTCCTCCGATTGACTGAGTCGCCCGGGTACCCG

TGTATCCAATAAACCCTCTTGCAGTTGCATCCGACTTGTGGTCTCGCTGTTCCTTGGGAG

GGTCTCCTCTGAGTGATTGACTACCCGTCAGCGGGGGTCTTTCATTTGGGGGCTCGTCCG

GGATCGCGGCCGCCACCGCGGTGGAGCTCCAATTCGCCCTATAGTGAGTCGTATTACGCG

CGCTCACTGGCCGTCGTTTTACAACGTCGTGACTGGGAAAACCCTGGCGTTACCCAACTT

AATCGCCTTGCAGCACATCCCCCTTTCGCCAGCTGGCGTAATAGCGAAGAGGCCCGCACC

GATCGCCCTTCCCAACAGTTGCGCAGCCTGAATGGCGAATGGGACGCGCCCTGTAGCGGC

GCATTAAGCGCGGCGGGTGTGGTGGTTACGCGCAGCGTGACCGCTACACTTGCCAGCGCC

CTAGCGCCCGCTCCTTTCGCTTTCTTCCCTTCCTTTCTCGCCACGTTCGCCGGCTTTCCC

CGTCAAGCTCTAAATCGGGGGCTCCCTTTAGGGTTCCGATTTAGTGCTTTACGGCACCTC

GACCCCAAAAAACTTGATTAGGGTGATGGTTCACGTAGTGGGCCATCGCCCTGATAGACG

GTTTTTCGCCCTTTGACGTTGGAGTCCACGTTCTTTAATAGTGGACTCTTGTTCCAAACT

GGAACAACACTCAACCCTATCTCGGTCTATTCTTTTGATTTATAAGGGATTTTGCCGATT

TCGGCCTATTGGTTAAAAAATGAGCTGATTTAACAAAAATTTAACGCGAATTTTAACAAA

ATATTAACGCTTACAATTTAGGTGGCACTTTTCGGGGAAATGTGCGCGGAACCCCTATTT

GTTTATTTTTCTAAATACATTCAAATATGTATCCGCTCATGAGACAATAACCCTGATAAA

TGCTTCAATAATATTGAAAAAGGAAGAGTATGAGTATTCAACATTTCCGTGTCGCCCTTA

TTCCCTTTTTTGCGGCATTTTGCCTTCCTGTTTTTGCTCACCCAGAAACGCTGGTGAAAG

TAAAAGATGCTGAAGATCAGTTGGGTGCACGAGTGGGTTACATCGAACTGGATCTCAACA

GCGGTAAGATCCTTGAGAGTTTTCGCCCCGAAGAACGTTTTCCAATGATGAGCACTTTTA

AAGTTCTGCTATGTGGCGCGGTATTATCCCGTATTGACGCCGGGCAAGAGCAACTCGGTC

GCCGCATACACTATTCTCAGAATGACTTGGTTGAGTACTCACCAGTCACAGAAAAGCATC

TTACGGATGGCATGACAGTAAGAGAATTATGCAGTGCTGCCATAACCATGAGTGATAACA

CTGCGGCCAACTTACTTCTGACAACGATCGGAGGACCGAAGGAGCTAACCGCTTTTTTGC

ACAACATGGGGGATCATGTAACTCGCCTTGATCGTTGGGAACCGGAGCTGAATGAAGCCA

TACCAAACGACGAGCGTGACACCACGATGCCTGTAGCAATGGCAACAACGTTGCGCAAAC

TATTAACTGGCGAACTACTTACTCTAGCTTCCCGGCAACAATTAATAGACTGGATGGAGG

CGGATAAAGTTGCAGGACCACTTCTGCGCTCGGCCCTTCCGGCTGGCTGGTTTATTGCTG

ATAAATCTGGAGCCGGTGAGCGTGGGTCTCGCGGTATCATTGCAGCACTGGGGCCAGATG

GTAAGCCCTCCCGTATCGTAGTTATCTACACGACGGGGAGTCAGGCAACTATGGATGAAC

GAAATAGACAGATCGCTGAGATAGGTGCCTCACTGATTAAGCATTGGTAACTGTCAGACC

AAGTTTACTCATATATACTTTAGATTGATTTAAAACTTCATTTTTAATTTAAAAGGATCT

AGGTGAAGATCCTTTTTGATAATCTCATGACCAAAATCCCTTAACGTGAGTTTTCGTTCC

ACTGAGCGTCAGACCCCGTAGAAAAGATCAAAGGATCTTCTTGAGATCCTTTTTTTCTGC

GCGTAATCTGCTGCTTGCAAACAAAAAAACCACCGCTACCAGCGGTGGTTTGTTTGCCGG

ATCAAGAGCTACCAACTCTTTTTCCGAAGGTAACTGGCTTCAGCAGAGCGCAGATACCAA

ATACTGTCCTTCTAGTGTAGCCGTAGTTAGGCCACCACTTCAAGAACTCTGTAGCACCGC

CTACATACCTCGCTCTGCTAATCCTGTTACCAGTGGCTGCTGCCAGTGGCGATAAGTCGT

GTCTTACCGGGTTGGACTCAAGACGATAGTTACCGGATAAGGCGCAGCGGTCGGGCTGAA

CGGGGGGTTCGTGCACACAGCCCAGCTTGGAGCGAACGACCTACACCGAACTGAGATACC

TACAGCGTGAGCTATGAGAAAGCGCCACGCTTCCCGAAGGGAGAAAGGCGGACAGGTATC

CGGTAAGCGGCAGGGTCGGAACAGGAGAGCGCACGAGGGAGCTTCCAGGGGGAAACGCCT

GGTATCTTTATAGTCCTGTCGGGTTTCGCCACCTCTGACTTGAGCGTCGATTTTTGTGAT

GCTCGTCAGGGGGGCGGAGCCTATGGAAAAACGCCAGCAACGCGGCCTTTTTACGGTTCC

TGGCCTTTTGCTGGCCTTTTGCTCACATGTTCTTTCCTGCGTTATCCCCTGATTCTGTGG

ATAACCGTATTACCGCCTTTGAGTGAGCTGATACCGCTCGCCGCAGCCGAACGACCGAGC

GCAGCGAGTCAGTGAGCGAGGAAGCGGAAGAGCGCCCAATACGCAAACCGCCTCTCCCCG

CGCGTTGGCCGATTCATTAATGCAGCTGGCACGACAGGTTTCCCGACTGGAAAGCGGGCA

GTGAGCGCAACGCAATTAATGTGAGTTAGCTCACTCATTAGGCACCCCAGGCTTTACACT

TTATGCTTCCGGCTCGTATGTTGTGTGGAATTGTGAGCGGATAACAATTTCACACAGGAA

ACAGCTATGACCATGATTACGCCAAGCGCGCAATTAACCCTCACTAAAGGGAACAAAAGC

TGGGTACCGGGCCCCCCCTCGAGAGTC

**SAGL**

7747 bp

5’LTR CMV promoter 1-530

R region 531-589

U5 region 590-665

Packaging signal 666-1042

ADA promoter 1052-1494

eGFP gene 1508-2227

ADA LCR 2249-4506

3’LTR U3 region 4565-4682

R region 4683-4749

U5 region 4750-4826

T7 primer site 4907-4876

CGTTACATAACTTACGGTAAATGGCCCGCCGGCTGACCGCCCAACGACCCCCGCCCATTG

ACGTCAATAATGACGTATGTTCCCATAGTAACGCCAATAGGGACTTTCCATTGACGTCAA

TGGGTGGAGTATTTACGGTAAACTGCCCACTTGGCAGTACATCAAGTGTATCATATGCCA

AGTACGCCCCCTATTGACGTCAATGACGGTAAATGGCCCGCCTGGCATTATGCCCAGTAC

ATGACCTTATGGGACTTTCCTACTTGGCAGTACATCTACGTATTAGTCATCGCTATTACC

ATGGTGATGCGGTTTTGGCAGTACATCAATGGGCGTGGATAGCGGTTTGACTCACGGGGA

TTTCCAAGTCTCCACCCCATTGACGTCAATGGGAGTTTGTTTTGGCACCAAAATCAACGG

GACTTTCCAAAATGTCGTAACAACTCCGCCCCATTGACGCAAATGGGCGGTAGGCGTGTA

CGGTGGGAGGTCTATATAAGCAGAGCTCGTTTAGTGAACCGCGCCAGTCTTCCGATAGAC

TGCGTCGCCCGGGTACCCGTATTCCCAATAAAGCCTCTTGCTGTTTGCATCCGAATCGTG

GTCTCGCTGTTCCTTGGGAGGGTCTCCTCTGAGTGATTGACTACCCACGACGGGGGTCTT

TCATTTGGGGGCTCGTCCGGGATTCGGAGACCCCTGCCCAGGGACCACCGACCCACCACC

GGGAGGTAAGCTGGCCACCAACTTATCTGTGTCTGTCCGATTGTCTAGTGTCTATGACTG

ATTTTATGCGCCTGCGTCGGTACTAGTTAGCTAACTAGCTCTGTATCTGGCGGACCCGTG

GTGGAACTGACGAGTTCGGAACACCCGGCCGCAACCCTGGGAGACGTCCCAGGGACTTCG

GGGGCCGTTTTTGTGGCCCGACCTGAGTCCAAAAATCCCGATCGTTTTGGACTCTTTGGT

GCACCCCCCTTAGGAGGAGGGATATGTGGTTCTGGTAGGAGACGAGAACCTAAAACAGTT

CCCGCCTCCGTCTGAATTTTTGCTAAGCTTCCTCGAGGCTTGCGATGCTCCCGGGGTCCC

CGCTTCTAGCTTGGGCCTGGCGCACAGCAGCGCCCAGACTGCAGGGGGACGCTTGAAAGT

TGCTGGAGGAGCCGGGGGGAAGGCAGCGCCCAGCGAGGCGGCTGGAGCGCGCGCCCACAG

GTGGGTCCGGTCGGGCGCCGCGGGGCCGTAGTTTTCGGGTCGGCGGGCGAGGACGCCGGG

TCCAGAATTCCAGGAAATGCGCGATCCAGGCCGGCGGGCGGGGCGGGGGCTCCGGCGAGA

GGGCGGGCCCCGGGAACGGCGGCGGGCGGGGCGGGAGGCGGGGCCCGGCCCGTTAAGAAG

AGCGTGGCCGGCCGCGGCCACCGCTGGCCCCAGGGAAAGCCGAGCGGCCACCGAGCCGGC

AGAGACCCACCGAGCGGCGGCGGAGGGAGCAGCGCCGGGGCGCACGAGGGCACCACCGGT

CGCCACCATGGTGAGCAAGGGCGAGGAGCTGTTCACCGGGGTGGTGCCCATCCTGGTCGA

GCTGGACGGCGACGTAAACGGCCACAAGTTCAGCGTGTCCGGCGAGGGCGAGGGCGATGC

CACCTACGGCAAGCTGACCCTGAAGTTCATCTGCACCACCGGCAAGCTGCCCGTGCCCTG

GCCCACCCTCGTGACCACCCTGACCTACGGCGTGCAGTGCTTCAGCCGCTACCCCGACCA

CATGAAGCAGCACGACTTCTTCAAGTCCGCCATGCCCGAAGGCTACGTCCAGGAGCGCAC

CATCTTCTTCAAGGACGACGGCAACTACAAGACCCGCGCCGAGGTGAAGTTCGAGGGCGA

CACCCTGGTGAACCGCATCGAGCTGAAGGGCATCGACTTCAAGGAGGACGGCAACATCCT

GGGGCACAAGCTGGAGTACAACTACAACAGCCACAACGTCTATATCATGGCCGACAAGCA

GAAGAACGGCATCAAGGTGAACTTCAAGATCCGCCACAACATCGAGGACGGCAGCGTGCA

GCTCGCCGACCACTACCAGCAGAACACCCCCATCGGCGACGGCCCCGTGCTGCTGCCCGA

CAACCACTACCTGAGCACCCAGTCCGCCCTGAGCAAAGACCCCAACGAGAAGCGCGATCA

CATGGTCCTGCTGGAGTTCGTGACCGCCGCCGGGATCACTCTCGGCATGGACGAGCTGTA

CAAGTAAAGCGGCCGCGACTCTAGAGGAGCATGCACCACCATGCCCGGCTAATTTTTTGT

ATTTTTAGTAGAGACGGGGTTTCTCCTTGTTGGCCAGGCTGGTCTCGAACTCCCGACTTC

AGGTGATCCGCCCACCTCAGCCTCCCAAAGTGCCGGGATTACAGGCATGAGCCACCGCAC

CCGGCCAAGGGACAGCAGTTTCTAAACTGTCCCTCTCTGATGCAGAGGGGAATTGGGGCT

AAATCAGCAATGTGCCTTTTCTGTCTCATATTTGAATGTCTACTCTGCACGAGGCGCTGT

CCTGCTTTGCATACAGTGACTCATTTAATGTTTATGTCAGCCCTCTGAGGAAGGTCCTGT

CCTATTATTAACTTCACTTATTATGAGGAAACTGAGACTCAGAGAGGGGAGGGAACTTGC

CAAAGTCACACAGCTGGCAAGCAGCAGAGCTAGACTTGAACCCAGATCTGCCTGCACTCA

AGTAGAAGCTGTTCATTGCTTTGCTCATTTGCCAATTCCACTTTATGCAAAAAAGAGGGG

GCAGTGTGGGGGGAAGAGTTAGAATCAGGGTGGCAGGGTGGGCCAGTGCATTAGCCCTGG

GCTTCAGATGTACTGGGGTTGAATTCCTGCCTGCCGCTTAGCAGCTAGGGTACCTCAGGT

AGACAACTCCTGAAACTCAGCTTCCCCCTCTGTAAAATGGGGTGACAAAACCAAGATCTT

GGGGTTCTTGGGGAAACTGACATGCTGATTGGTTTTTGTACAGTGCCTGGCTGGTAACAG

CAGGCCCTCAGGGGTGCGTTTCCTTCCTGGGGACTGGAGTGGGGGTTGCAGTAGACTCTG

GGAGGCCTCTCCAGCTGCAGAATCTCCCTCCTCCCTCCTCCTTTTTGTCTTCCTGACACA

AAACCCACCAGCTGCACTTCTTTGGGCTTGCAGTGGCTTTCAGTTACCAGAGCCACCTGT

TAAAACAAAAATGTGCCTAGGAAGAGCCTGCCTTACCCATTTTGACTCACATGGCAGTTG

GTGGTGGAGGGGAACAAAGGAGACTGAGTTTCATCGAAGCCTTTTGCTTCGGAGGAGGAA

GGGAGGATCAGAGAGAGGAAGTGGTCTGTGTTCACACAGGGAGGCAGGGGAGGCCAGGCA

GCTTCCCAATCCTGCATTCAACCTCAGGGTGGGCTTGACCTGGGTGGCTGGGGGCCCTGT

GATCCAGGAGAGACTTGTCCACCTGCTCAGGTGTCTTGAAGGGGTCCCTGTGGTACCCCC

TGGGCGGGGCAAGGTAGTAGGACCATGGTCTGGCTGGGGAGGTGGAGAGGAGCAGGCTGT

GGGCGCAGAGTGAGGTTGGAATCTGTATTTACCCAAGGTGTTGGGGGTAGGCTTGCCCTC

AGCCCTTAATGTTCTCAGGCCCCTGAGCAGTTGTGGGGGATAACCTCTGCACTCCTAGTG

ACCAGGGAGCTAGAACAGCAAGGAATTTGAACTTGGACACCAGCTGGGGTCAGGCTCTCT

GGGTCTGAGTCCTGATTTCCCACTTTCCAGCTAGAGGAGCTTGAATGAGTCATTTAACTT

CACGGTGCCTCAGTTTCCCCTCTCTAAAATGAGAATTATACCCATACCCACCTCTCAAAC

ACCAAGTGCAGGCCTGGCTCAGAGCAGGTGCTGCAGCAATAGCTGCCATTGGTCAGCATC

ATCATCATGGTTGGTAATGGTCCTACTTTGACTTTTGAGACAGAGTCTCACTCTGTCGCC

CAGGCTGGAGTGCAGTGGTGCAATCTCGGCTCACTACAACCTCTGCTCCCGGGTTCAAGT

GATTCTTCTGCCTCAGTCTCCCAAGTAGTTGGGATTACAGGTGTGCGCCACCATGCCTGG

CTAATTTTTGTGTTTTTAGTAGAGACAGGGTTTCACCATGTTGGCCATAACAATGGCTGT

CCTTGAAGTAGTCCTGGGTACCAGGTGCCTTCAGTGACTTTTTTTTTTTTTTTTTTTTTG

AGCTGGAGTCTTCCTCTGTCACCCAAGCTGGAGTGCAGTGGCACGATTTTGGCTCACTGC

AACCTCTGCCTCCTGGGTTCATGCGATCCTCCTGCCTCAGCCTCCCAAGTAGCTGGGACT

TGGGATACACTTGCCCCCGCTGGTCCTCCCTTCCACCTCTGTGAAGAGGAGGTCTCAAAC

TCCTGGCCTCAAGTGATCCACCCACCTCAGCCTCCCAAAGTGCTGGGATTTCAAGAGTGA

GCCACCGCACCTGGCCCCTGTTTAGATGTTAGCATCAGTGACCCAGCACCTTGCTATGTG

GCATGCCTGCAGGTCGACCATAGATAAAATAAAAGATTTTATTTAGTCTCCAGAAAAAGG

GGGGAATGAAAGACCCCACCTGTAGGTTTGGCAAGCTAGCGATATCAGTTCGCTTCTCGC

TTCTGTTCGCGCGCTTCTGCTCCCCGAGCTCAATAAAAGAGCCCACAACCCCTCACTCGG

GGCGCCAGTCCTCCGATTGACTGAGTCGCCCGGGTACCCGTGTATCCAATAAACCCTCTT

GCAGTTGCATCCGACTTGTGGTCTCGCTGTTCCTTGGGAGGGTCTCCTCTGAGTGATTGA

CTACCCGTCAGCGGGGGTCTTTCATTTGGGGGCTCGTCCGGGATCGCGGCCGCCACCGCG

GTGGAGCTCCAATTCGCCCTATAGTGAGTCGTATTACGCGCGCTCACTGGCCGTCGTTTT

ACAACGTCGTGACTGGGAAAACCCTGGCGTTACCCAACTTAATCGCCTTGCAGCACATCC

CCCTTTCGCCAGCTGGCGTAATAGCGAAGAGGCCCGCACCGATCGCCCTTCCCAACAGTT

GCGCAGCCTGAATGGCGAATGGGACGCGCCCTGTAGCGGCGCATTAAGCGCGGCGGGTGT

GGTGGTTACGCGCAGCGTGACCGCTACACTTGCCAGCGCCCTAGCGCCCGCTCCTTTCGC

TTTCTTCCCTTCCTTTCTCGCCACGTTCGCCGGCTTTCCCCGTCAAGCTCTAAATCGGGG

GCTCCCTTTAGGGTTCCGATTTAGTGCTTTACGGCACCTCGACCCCAAAAAACTTGATTA

GGGTGATGGTTCACGTAGTGGGCCATCGCCCTGATAGACGGTTTTTCGCCCTTTGACGTT

GGAGTCCACGTTCTTTAATAGTGGACTCTTGTTCCAAACTGGAACAACACTCAACCCTAT

CTCGGTCTATTCTTTTGATTTATAAGGGATTTTGCCGATTTCGGCCTATTGGTTAAAAAA

TGAGCTGATTTAACAAAAATTTAACGCGAATTTTAACAAAATATTAACGCTTACAATTTA

GGTGGCACTTTTCGGGGAAATGTGCGCGGAACCCCTATTTGTTTATTTTTCTAAATACAT

TCAAATATGTATCCGCTCATGAGACAATAACCCTGATAAATGCTTCAATAATATTGAAAA

AGGAAGAGTATGAGTATTCAACATTTCCGTGTCGCCCTTATTCCCTTTTTTGCGGCATTT

TGCCTTCCTGTTTTTGCTCACCCAGAAACGCTGGTGAAAGTAAAAGATGCTGAAGATCAG

TTGGGTGCACGAGTGGGTTACATCGAACTGGATCTCAACAGCGGTAAGATCCTTGAGAGT

TTTCGCCCCGAAGAACGTTTTCCAATGATGAGCACTTTTAAAGTTCTGCTATGTGGCGCG

GTATTATCCCGTATTGACGCCGGGCAAGAGCAACTCGGTCGCCGCATACACTATTCTCAG

AATGACTTGGTTGAGTACTCACCAGTCACAGAAAAGCATCTTACGGATGGCATGACAGTA

AGAGAATTATGCAGTGCTGCCATAACCATGAGTGATAACACTGCGGCCAACTTACTTCTG

ACAACGATCGGAGGACCGAAGGAGCTAACCGCTTTTTTGCACAACATGGGGGATCATGTA

ACTCGCCTTGATCGTTGGGAACCGGAGCTGAATGAAGCCATACCAAACGACGAGCGTGAC

ACCACGATGCCTGTAGCAATGGCAACAACGTTGCGCAAACTATTAACTGGCGAACTACTT

ACTCTAGCTTCCCGGCAACAATTAATAGACTGGATGGAGGCGGATAAAGTTGCAGGACCA

CTTCTGCGCTCGGCCCTTCCGGCTGGCTGGTTTATTGCTGATAAATCTGGAGCCGGTGAG

CGTGGGTCTCGCGGTATCATTGCAGCACTGGGGCCAGATGGTAAGCCCTCCCGTATCGTA

GTTATCTACACGACGGGGAGTCAGGCAACTATGGATGAACGAAATAGACAGATCGCTGAG

ATAGGTGCCTCACTGATTAAGCATTGGTAACTGTCAGACCAAGTTTACTCATATATACTT

TAGATTGATTTAAAACTTCATTTTTAATTTAAAAGGATCTAGGTGAAGATCCTTTTTGAT

AATCTCATGACCAAAATCCCTTAACGTGAGTTTTCGTTCCACTGAGCGTCAGACCCCGTA

GAAAAGATCAAAGGATCTTCTTGAGATCCTTTTTTTCTGCGCGTAATCTGCTGCTTGCAA

ACAAAAAAACCACCGCTACCAGCGGTGGTTTGTTTGCCGGATCAAGAGCTACCAACTCTT

TTTCCGAAGGTAACTGGCTTCAGCAGAGCGCAGATACCAAATACTGTCCTTCTAGTGTAG

CCGTAGTTAGGCCACCACTTCAAGAACTCTGTAGCACCGCCTACATACCTCGCTCTGCTA

ATCCTGTTACCAGTGGCTGCTGCCAGTGGCGATAAGTCGTGTCTTACCGGGTTGGACTCA

AGACGATAGTTACCGGATAAGGCGCAGCGGTCGGGCTGAACGGGGGGTTCGTGCACACAG

CCCAGCTTGGAGCGAACGACCTACACCGAACTGAGATACCTACAGCGTGAGCTATGAGAA

AGCGCCACGCTTCCCGAAGGGAGAAAGGCGGACAGGTATCCGGTAAGCGGCAGGGTCGGA

ACAGGAGAGCGCACGAGGGAGCTTCCAGGGGGAAACGCCTGGTATCTTTATAGTCCTGTC

GGGTTTCGCCACCTCTGACTTGAGCGTCGATTTTTGTGATGCTCGTCAGGGGGGCGGAGC

CTATGGAAAAACGCCAGCAACGCGGCCTTTTTACGGTTCCTGGCCTTTTGCTGGCCTTTT

GCTCACATGTTCTTTCCTGCGTTATCCCCTGATTCTGTGGATAACCGTATTACCGCCTTT

GAGTGAGCTGATACCGCTCGCCGCAGCCGAACGACCGAGCGCAGCGAGTCAGTGAGCGAG

GAAGCGGAAGAGCGCCCAATACGCAAACCGCCTCTCCCCGCGCGTTGGCCGATTCATTAA

TGCAGCTGGCACGACAGGTTTCCCGACTGGAAAGCGGGCAGTGAGCGCAACGCAATTAAT

GTGAGTTAGCTCACTCATTAGGCACCCCAGGCTTTACACTTTATGCTTCCGGCTCGTATG

TTGTGTGGAATTGTGAGCGGATAACAATTTCACACAGGAAACAGCTATGACCATGATTAC

GCCAAGCGCGCAATTAACCCTCACTAAAGGGAACAAAAGCTGGGTACCGGGCCCCCCCTC

GAGAGTC

**SAGiL**

7747 bp

5’LTR CMV promoter 1-530

R region 531-589

U5 region 590-665

Packaging signal 666-1042

ADA promoter 1052-1494

eGFP gene 1508-2227

ADA LCR 4515-2258 (reverse orientation)

3’LTR U3 region 4565-4682

R region 4683-4749

U5 region 4750-4826

T7 primer site 4907-4876

CGTTACATAACTTACGGTAAATGGCCCGCCGGCTGACCGCCCAACGACCCCCGCCCATTGACGTCAATAATGACGTATGTTCCCATAGTAACGCCAATAGGGACTTTCCATTGACGTCAATGGGTGGAGTATTTACGGTAAACTGCCCACTTGGCAGTACATCAAGTGTATCATATGCCAAGTACGCCCCCTATTGACGTCAATGACGGTAAATGGCCCGCCTGGCATTATGCCCAGTACATGACCTTATGGGACTTTCCTACTTGGCAGTACATCTACGTATTAGTCATCGCTATTACCATGGTGATGCGGTTTTGGCAGTACATCAATGGGCGTGGATAGCGGTTTGACTCACGGGGATTTCCAAGTCTCCACCCCATTGACGTCAATGGGAGTTTGTTTTGGCACCAAAATCAACGGGACTTTCCAAAATGTCGTAACAACTCCGCCCCATTGACGCAAATGGGCGGTAGGCGTGTACGGTGGGAGGTCTATATAAGCAGAGCTCGTTTAGTGAACCGCGCCAGTCTTCCGATAGACTGCGTCGCCCGGGTACCCGTATTCCCAATAAAGCCTCTTGCTGTTTGCATCCGAATCGTGGTCTCGCTGTTCCTTGGGAGGGTCTCCTCTGAGTGATTGACTACCCACGACGGGGGTCTTTCATTTGGGGGCTCGTCCGGGATTCGGAGACCCCTGCCCAGGGACCACCGACCCACCACCGGGAGGTAAGCTGGCCACCAACTTATCTGTGTCTGTCCGATTGTCTAGTGTCTATGACTGATTTTATGCGCCTGCGTCGGTACTAGTTAGCTAACTAGCTCTGTATCTGGCGGACCCGTGGTGGAACTGACGAGTTCGGAACACCCGGCCGCAACCCTGGGAGACGTCCCAGGGACTTCGGGGGCCGTTTTTGTGGCCCGACCTGAGTCCAAAAATCCCGATCGTTTTGGACTCTTTGGTGCACCCCCCTTAGGAGGAGGGATATGTGGTTCTGGTAGGAGACGAGAACCTAAAACAGTTCCCGCCTCCGTCTGAATTTTTGCTAAGCTTCCTCGAGGCTTGCGATGCTCCCGGGGTCCCCGCTTCTAGCTTGGGCCTGGCGCACAGCAGCGCCCAGACTGCAGGGGGACGCTTGAAAGTTGCTGGAGGAGCCGGGGGGAAGGCAGCGCCCAGCGAGGCGGCTGGAGCGCGCGCCCACAGGTGGGTCCGGTCGGGCGCCGCGGGGCCGTAGTTTTCGGGTCGGCGGGCGAGGACGCCGGGTCCAGAATTCCAGGAAATGCGCGATCCAGGCCGGCGGGCGGGGCGGGGGCTCCGGCGAGAGGGCGGGCCCCGGGAACGGCGGCGGGCGGGGCGGGAGGCGGGGCCCGGCCCGTTAAGAAGAGCGTGGCCGGCCGCGGCCACCGCTGGCCCCAGGGAAAGCCGAGCGGCCACCGAGCCGGCAGAGACCCACCGAGCGGCGGCGGAGGGAGCAGCGCCGGGGCGCACGAGGGCACCACCGGTCGCCACCATGGTGAGCAAGGGCGAGGAGCTGTTCACCGGGGTGGTGCCCATCCTGGTCGAGCTGGACGGCGACGTAAACGGCCACAAGTTCAGCGTGTCCGGCGAGGGCGAGGGCGATGCCACCTACGGCAAGCTGACCCTGAAGTTCATCTGCACCACCGGCAAGCTGCCCGTGCCCTGGCCCACCCTCGTGACCACCCTGACCTACGGCGTGCAGTGCTTCAGCCGCTACCCCGACCACATGAAGCAGCACGACTTCTTCAAGTCCGCCATGCCCGAAGGCTACGTCCAGGAGCGCACCATCTTCTTCAAGGACGACGGCAACTACAAGACCCGCGCCGAGGTGAAGTTCGAGGGCGACACCCTGGTGAACCGCATCGAGCTGAAGGGCATCGACTTCAAGGAGGACGGCAACATCCTGGGGCACAAGCTGGAGTACAACTACAACAGCCACAACGTCTATATCATGGCCGACAAGCAGAAGAACGGCATCAAGGTGAACTTCAAGATCCGCCACAACATCGAGGACGGCAGCGTGCAGCTCGCCGACCACTACCAGCAGAACACCCCCATCGGCGACGGCCCCGTGCTGCTGCCCGACAACCACTACCTGAGCACCCAGTCCGCCCTGAGCAAAGACCCCAACGAGAAGCGCGATCACATGGTCCTGCTGGAGTTCGTGACCGCCGCCGGGATCACTCTCGGCATGGACGAGCTGTACAAGTAAAGCGGCCGCGACTCTAGAGTCGACCTGCAGGCATGCCACATAGCAAGGTGCTGGGTCACTGATGCTAACATCTAAACAGGGGCCAGGTGCGGTGGCTCACTCTTGAAATCCCAGCACTTTGGGAGGCTGAGGTGGGTGGATCACTTGAGGCCAGGAGTTTGAGACCTCCTCTTCACAGAGGTGGAAGGGAGGACCAGCGGGGGCAAGTGTATCCCAAGTCCCAGCTACTTGGGAGGCTGAGGCAGGAGGATCGCATGAACCCAGGAGGCAGAGGTTGCAGTGAGCCAAAATCGTGCCACTGCACTCCAGCTTGGGTGACAGAGGAAGACTCCAGCTCAAAAAAAAAAAAAAAAAAAAAGTCACTGAAGGCACCTGGTACCCAGGACTACTTCAAGGACAGCCATTGTTATGGCCAACATGGTGAAACCCTGTCTCTACTAAAAACACAAAAATTAGCCAGGCATGGTGGCGCACACCTGTAATCCCAACTACTTGGGAGACTGAGGCAGAAGAATCACTTGAACCCGGGAGCAGAGGTTGTAGTGAGCCGAGATTGCACCACTGCACTCCAGCCTGGGCGACAGAGTGAGACTCTGTCTCAAAAGTCAAAGTAGGACCATTACCAACCATGATGATGATGCTGACCAATGGCAGCTATTGCTGCAGCACCTGCTCTGAGCCAGGCCTGCACTTGGTGTTTGAGAGGTGGGTATGGGTATAATTCTCATTTTAGAGAGGGGAAACTGAGGCACCGTGAAGTTAAATGACTCATTCAAGCTCCTCTAGCTGGAAAGTGGGAAATCAGGACTCAGACCCAGAGAGCCTGACCCCAGCTGGTGTCCAAGTTCAAATTCCTTGCTGTTCTAGCTCCCTGGTCACTAGGAGTGCAGAGGTTATCCCCCACAACTGCTCAGGGGCCTGAGAACATTAAGGGCTGAGGGCAAGCCTACCCCCAACACCTTGGGTAAATACAGATTCCAACCTCACTCTGCGCCCACAGCCTGCTCCTCTCCACCTCCCCAGCCAGACCATGGTCCTACTACCTTGCCCCGCCCAGGGGGTACCACAGGGACCCCTTCAAGACACCTGAGCAGGTGGACAAGTCTCTCCTGGATCACAGGGCCCCCAGCCACCCAGGTCAAGCCCACCCTGAGGTTGAATGCAGGATTGGGAAGCTGCCTGGCCTCCCCTGCCTCCCTGTGTGAACACAGACCACTTCCTCTCTCTGATCCTCCCTTCCTCCTCCGAAGCAAAAGGCTTCGATGAAACTCAGTCTCCTTTGTTCCCCTCCACCACCAACTGCCATGTGAGTCAAAATGGGTAAGGCAGGCTCTTCCTAGGCACATTTTTGTTTTAACAGGTGGCTCTGGTAACTGAAAGCCACTGCAAGCCCAAAGAAGTGCAGCTGGTGGGTTTTGTGTCAGGAAGACAAAAAGGAGGAGGGAGGAGGGAGATTCTGCAGCTGGAGAGGCCTCCCAGAGTCTACTGCAACCCCCACTCCAGTCCCCAGGAAGGAAACGCACCCCTGAGGGCCTGCTGTTACCAGCCAGGCACTGTACAAAAACCAATCAGCATGTCAGTTTCCCCAAGAACCCCAAGATCTTGGTTTTGTCACCCCATTTTACAGAGGGGGAAGCTGAGTTTCAGGAGTTGTCTACCTGAGGTACCCTAGCTGCTAAGCGGCAGGCAGGAATTCAACCCCAGTACATCTGAAGCCCAGGGCTAATGCACTGGCCCACCCTGCCACCCTGATTCTAACTCTTCCCCCCACACTGCCCCCTCTTTTTTGCATAAAGTGGAATTGGCAAATGAGCAAAGCAATGAACAGCTTCTACTTGAGTGCAGGCAGATCTGGGTTCAAGTCTAGCTCTGCTGCTTGCCAGCTGTGTGACTTTGGCAAGTTCCCTCCCCTCTCTGAGTCTCAGTTTCCTCATAATAAGTGAAGTTAATAATAGGACAGGACCTTCCTCAGAGGGCTGACATAAACATTAAATGAGTCACTGTATGCAAAGCAGGACAGCGCCTCGTGCAGAGTAGACATTCAAATATGAGACAGAAAAGGCACATTGCTGATTTAGCCCCAATTCCCCTCTGCATCAGAGAGGGACAGTTTAGAAACTGCTGTCCCTTGGCCGGGTGCGGTGGCTCATGCCTGTAATCCCGGCACTTTGGGAGGCTGAGGTGGGCGGATCACCTGAAGTCGGGAGTTCGAGACCAGCCTGGCCAACAAGGAGAAACCCCGTCTCTACTAAAAATACAAAAAATTAGCCGGGCATGGTGGTGCATGCTCCTCTAGGACCATAGATAAAATAAAAGATTTTATTTAGTCTCCAGAAAAAGGGGGGAATGAAAGACCCCACCTGTAGGTTTGGCAAGCTAGCGATATCAGTTCGCTTCTCGCTTCTGTTCGCGCGCTTCTGCTCCCCGAGCTCAATAAAAGAGCCCACAACCCCTCACTCGGGGCGCCAGTCCTCCGATTGACTGAGTCGCCCGGGTACCCGTGTATCCAATAAACCCTCTTGCAGTTGCATCCGACTTGTGGTCTCGCTGTTCCTTGGGAGGGTCTCCTCTGAGTGATTGACTACCCGTCAGCGGGGGTCTTTCATTTGGGGGCTCGTCCGGGATCGCGGCCGCCACCGCGGTGGAGCTCCAATTCGCCCTATAGTGAGTCGTATTACGCGCGCTCACTGGCCGTCGTTTTACAACGTCGTGACTGGGAAAACCCTGGCGTTACCCAACTTAATCGCCTTGCAGCACATCCCCCTTTCGCCAGCTGGCGTAATAGCGAAGAGGCCCGCACCGATCGCCCTTCCCAACAGTTGCGCAGCCTGAATGGCGAATGGGACGCGCCCTGTAGCGGCGCATTAAGCGCGGCGGGTGTGGTGGTTACGCGCAGCGTGACCGCTACACTTGCCAGCGCCCTAGCGCCCGCTCCTTTCGCTTTCTTCCCTTCCTTTCTCGCCACGTTCGCCGGCTTTCCCCGTCAAGCTCTAAATCGGGGGCTCCCTTTAGGGTTCCGATTTAGTGCTTTACGGCACCTCGACCCCAAAAAACTTGATTAGGGTGATGGTTCACGTAGTGGGCCATCGCCCTGATAGACGGTTTTTCGCCCTTTGACGTTGGAGTCCACGTTCTTTAATAGTGGACTCTTGTTCCAAACTGGAACAACACTCAACCCTATCTCGGTCTATTCTTTTGATTTATAAGGGATTTTGCCGATTTCGGCCTATTGGTTAAAAAATGAGCTGATTTAACAAAAATTTAACGCGAATTTTAACAAAATATTAACGCTTACAATTTAGGTGGCACTTTTCGGGGAAATGTGCGCGGAACCCCTATTTGTTTATTTTTCTAAATACATTCAAATATGTATCCGCTCATGAGACAATAACCCTGATAAATGCTTCAATAATATTGAAAAAGGAAGAGTATGAGTATTCAACATTTCCGTGTCGCCCTTATTCCCTTTTTTGCGGCATTTTGCCTTCCTGTTTTTGCTCACCCAGAAACGCTGGTGAAAGTAAAAGATGCTGAAGATCAGTTGGGTGCACGAGTGGGTTACATCGAACTGGATCTCAACAGCGGTAAGATCCTTGAGAGTTTTCGCCCCGAAGAACGTTTTCCAATGATGAGCACTTTTAAAGTTCTGCTATGTGGCGCGGTATTATCCCGTATTGACGCCGGGCAAGAGCAACTCGGTCGCCGCATACACTATTCTCAGAATGACTTGGTTGAGTACTCACCAGTCACAGAAAAGCATCTTACGGATGGCATGACAGTAAGAGAATTATGCAGTGCTGCCATAACCATGAGTGATAACACTGCGGCCAACTTACTTCTGACAACGATCGGAGGACCGAAGGAGCTAACCGCTTTTTTGCACAACATGGGGGATCATGTAACTCGCCTTGATCGTTGGGAACCGGAGCTGAATGAAGCCATACCAAACGACGAGCGTGACACCACGATGCCTGTAGCAATGGCAACAACGTTGCGCAAACTATTAACTGGCGAACTACTTACTCTAGCTTCCCGGCAACAATTAATAGACTGGATGGAGGCGGATAAAGTTGCAGGACCACTTCTGCGCTCGGCCCTTCCGGCTGGCTGGTTTATTGCTGATAAATCTGGAGCCGGTGAGCGTGGGTCTCGCGGTATCATTGCAGCACTGGGGCCAGATGGTAAGCCCTCCCGTATCGTAGTTATCTACACGACGGGGAGTCAGGCAACTATGGATGAACGAAATAGACAGATCGCTGAGATAGGTGCCTCACTGATTAAGCATTGGTAACTGTCAGACCAAGTTTACTCATATATACTTTAGATTGATTTAAAACTTCATTTTTAATTTAAAAGGATCTAGGTGAAGATCCTTTTTGATAATCTCATGACCAAAATCCCTTAACGTGAGTTTTCGTTCCACTGAGCGTCAGACCCCGTAGAAAAGATCAAAGGATCTTCTTGAGATCCTTTTTTTCTGCGCGTAATCTGCTGCTTGCAAACAAAAAAACCACCGCTACCAGCGGTGGTTTGTTTGCCGGATCAAGAGCTACCAACTCTTTTTCCGAAGGTAACTGGCTTCAGCAGAGCGCAGATACCAAATACTGTCCTTCTAGTGTAGCCGTAGTTAGGCCACCACTTCAAGAACTCTGTAGCACCGCCTACATACCTCGCTCTGCTAATCCTGTTACCAGTGGCTGCTGCCAGTGGCGATAAGTCGTGTCTTACCGGGTTGGACTCAAGACGATAGTTACCGGATAAGGCGCAGCGGTCGGGCTGAACGGGGGGTTCGTGCACACAGCCCAGCTTGGAGCGAACGACCTACACCGAACTGAGATACCTACAGCGTGAGCTATGAGAAAGCGCCACGCTTCCCGAAGGGAGAAAGGCGGACAGGTATCCGGTAAGCGGCAGGGTCGGAACAGGAGAGCGCACGAGGGAGCTTCCAGGGGGAAACGCCTGGTATCTTTATAGTCCTGTCGGGTTTCGCCACCTCTGACTTGAGCGTCGATTTTTGTGATGCTCGTCAGGGGGGCGGAGCCTATGGAAAAACGCCAGCAACGCGGCCTTTTTACGGTTCCTGGCCTTTTGCTGGCCTTTTGCTCACATGTTCTTTCCTGCGTTATCCCCTGATTCTGTGGATAACCGTATTACCGCCTTTGAGTGAGCTGATACCGCTCGCCGCAGCCGAACGACCGAGCGCAGCGAGTCAGTGAGCGAGGAAGCGGAAGAGCGCCCAATACGCAAACCGCCTCTCCCCGCGCGTTGGCCGATTCATTAATGCAGCTGGCACGACAGGTTTCCCGACTGGAAAGCGGGCAGTGAGCGCAACGCAATTAATGTGAGTTAGCTCACTCATTAGGCACCCCAGGCTTTACACTTTATGCTTCCGGCTCGTATGTTGTGTGGAATTGTGAGCGGATAACAATTTCACACAGGAAACAGCTATGACCATGATTACGCCAAGCGCGCAATTAACCCTCACTAAAGGGAACAAAAGCTGGGTACCGGGCCCCCCCTCGAGAGTC

**SHGAL**

8350 bp

5’LTR CMV promoter 1-530

R region 531-589

U5 region 590-665

Packaging signal 666-1042

hGH pA 1630-1046 (reverse orientation)

eGFP gene 2367-1648 (reverse orientation)

ADA promoter 2823-2381 (reverse orientation)

ADA LCR 2852-5109

3’LTR DU3 region 5168-5285

R region 5286-5352

U5 region 5353-5429

T7 primer site 5510-5479

CGTTACATAACTTACGGTAAATGGCCCGCCGGCTGACCGCCCAACGACCCCCGCCCATTG

ACGTCAATAATGACGTATGTTCCCATAGTAACGCCAATAGGGACTTTCCATTGACGTCAA

TGGGTGGAGTATTTACGGTAAACTGCCCACTTGGCAGTACATCAAGTGTATCATATGCCA

AGTACGCCCCCTATTGACGTCAATGACGGTAAATGGCCCGCCTGGCATTATGCCCAGTAC

ATGACCTTATGGGACTTTCCTACTTGGCAGTACATCTACGTATTAGTCATCGCTATTACC

ATGGTGATGCGGTTTTGGCAGTACATCAATGGGCGTGGATAGCGGTTTGACTCACGGGGA

TTTCCAAGTCTCCACCCCATTGACGTCAATGGGAGTTTGTTTTGGCACCAAAATCAACGG

GACTTTCCAAAATGTCGTAACAACTCCGCCCCATTGACGCAAATGGGCGGTAGGCGTGTA

CGGTGGGAGGTCTATATAAGCAGAGCTCGTTTAGTGAACCGCGCCAGTCTTCCGATAGAC

TGCGTCGCCCGGGTACCCGTATTCCCAATAAAGCCTCTTGCTGTTTGCATCCGAATCGTG

GTCTCGCTGTTCCTTGGGAGGGTCTCCTCTGAGTGATTGACTACCCACGACGGGGGTCTT

TCATTTGGGGGCTCGTCCGGGATTCGGAGACCCCTGCCCAGGGACCACCGACCCACCACC

GGGAGGTAAGCTGGCCACCAACTTATCTGTGTCTGTCCGATTGTCTAGTGTCTATGACTG

ATTTTATGCGCCTGCGTCGGTACTAGTTAGCTAACTAGCTCTGTATCTGGCGGACCCGTG

GTGGAACTGACGAGTTCGGAACACCCGGCCGCAACCCTGGGAGACGTCCCAGGGACTTCG

GGGGCCGTTTTTGTGGCCCGACCTGAGTCCAAAAATCCCGATCGTTTTGGACTCTTTGGT

GCACCCCCCTTAGGAGGAGGGATATGTGGTTCTGGTAGGAGACGAGAACCTAAAACAGTT

CCCGCCTCCGTCTGAATTTTTGCTAAGCTTGCCAAGCAAACAACTCAAATGTCCAACTGG

CTGGGCATGGCCAGGTAGCCCATGCTGTGTCTGGACGTCCTTCTGCTGGTATAATTATTT

TAAAATCACAGGACAGGGAAGGGCCCAGTGGCTCATACCTGTAATCCCAGCAATTTGGGA

GGCCGAGGCGGGCGGATTACCTGAGGTCAGGAGATGGAGACCAGACTGGCCAATATGGTG

AAACCCCGTCTCTACCAAAAATACAAAAATTAGCTGAGCCTGGTCGTGCATGCCTGGAAT

CCCAACTATTCGGGAGACTGAGGCAGGAGAATCGCTTGAACCCAGGAGGCGGAGATTGCA

GCGAGCCAAGATCGTGCCACTGCACTCCAGCCTGGTTCCCAATAGACCCTGAAGGCCCTA

CAGGTTGTCTTCCCAACCTGCCCCTTGCTCCATACCACCCGCCTCCACCCCATAATATTA

TACAAGGACACCTAGTCAAACAAAATGATGCAACTTAATTTTATTAGGACAAGGCTGGTG

GGCACTGGAGTAGCACCTTCCACGACCAGGAGAGGCACTGGGGAGGGGTCACAGGGATGC

CACGTTAACTCTAGAGTCGCGGCCGCTTTACTTGTACAGCTCGTCCATGCCGAGAGTGAT

CCCGGCGGCGGTCACGAACTCCAGCAGGACCATGTGATCGCGCTTCTCGTTGGGGTCTTT

GCTCAGGGCGGACTGGGTGCTCAGGTAGTGGTTGTCGGGCAGCAGCACGGGGCCGTCGCC

GATGGGGGTGTTCTGCTGGTAGTGGTCGGCGAGCTGCACGCTGCCGTCCTCGATGTTGTG

GCGGATCTTGAAGTTCACCTTGATGCCGTTCTTCTGCTTGTCGGCCATGATATAGACGTT

GTGGCTGTTGTAGTTGTACTCCAGCTTGTGCCCCAGGATGTTGCCGTCCTCCTTGAAGTC

GATGCCCTTCAGCTCGATGCGGTTCACCAGGGTGTCGCCCTCGAACTTCACCTCGGCGCG

GGTCTTGTAGTTGCCGTCGTCCTTGAAGAAGATGGTGCGCTCCTGGACGTAGCCTTCGGG

CATGGCGGACTTGAAGAAGTCGTGCTGCTTCATGTGGTCGGGGTAGCGGCTGAAGCACTG

CACGCCGTAGGTCAGGGTGGTCACGAGGGTGGGCCAGGGCACGGGCAGCTTGCCGGTGGT

GCAGATGAACTTCAGGGTCAGCTTGCCGTAGGTGGCATCGCCCTCGCCCTCGCCGGACAC

GCTGAACTTGTGGCCGTTTACGTCGCCGTCCAGCTCGACCAGGATGGGCACCACCCCGGT

GAACAGCTCCTCGCCCTTGCTCACCATGGTGGCGACCGGTGGTGCCCTCGTGCGCCCCGG

CGCTGCTCCCTCCGCCGCCGCTCGGTGGGTCTCTGCCGGCTCGGTGGCCGCTCGGCTTTC

CCTGGGGCCAGCGGTGGCCGCGGCCGGCCACGCTCTTCTTAACGGGCCGGGCCCCGCCTC

CCGCCCCGCCCGCCGCCGTTCCCGGGGCCCGCCCTCTCGCCGGAGCCCCCGCCCCGCCCG

CCGGCCTGGATCGCGCATTTCCTGGAATTCTGGACCCGGCGTCCTCGCCCGCCGACCCGA

AAACTACGGCCCCGCGGCGCCCGACCGGACCCACCTGTGGGCGCGCGCTCCAGCCGCCTC

GCTGGGCGCTGCCTTCCCCCCGGCTCCTCCAGCAACTTTCAAGCGTCCCCCTGCAGTCTG

GGCGCTGCTGTGCGCCAGGCCCAAGCTAGAAGCGGGGACCCCGGGAGCATCGCAAGCCTC

GAGGAAGCTTGAATTCGGATCCCCTAGAGGAGCATGCACCACCATGCCCGGCTAATTTTT

TGTATTTTTAGTAGAGACGGGGTTTCTCCTTGTTGGCCAGGCTGGTCTCGAACTCCCGAC

TTCAGGTGATCCGCCCACCTCAGCCTCCCAAAGTGCCGGGATTACAGGCATGAGCCACCG

CACCCGGCCAAGGGACAGCAGTTTCTAAACTGTCCCTCTCTGATGCAGAGGGGAATTGGG

GCTAAATCAGCAATGTGCCTTTTCTGTCTCATATTTGAATGTCTACTCTGCACGAGGCGC

TGTCCTGCTTTGCATACAGTGACTCATTTAATGTTTATGTCAGCCCTCTGAGGAAGGTCC

TGTCCTATTATTAACTTCACTTATTATGAGGAAACTGAGACTCAGAGAGGGGAGGGAACT

TGCCAAAGTCACACAGCTGGCAAGCAGCAGAGCTAGACTTGAACCCAGATCTGCCTGCAC

TCAAGTAGAAGCTGTTCATTGCTTTGCTCATTTGCCAATTCCACTTTATGCAAAAAAGAG

GGGGCAGTGTGGGGGGAAGAGTTAGAATCAGGGTGGCAGGGTGGGCCAGTGCATTAGCCC

TGGGCTTCAGATGTACTGGGGTTGAATTCCTGCCTGCCGCTTAGCAGCTAGGGTACCTCA

GGTAGACAACTCCTGAAACTCAGCTTCCCCCTCTGTAAAATGGGGTGACAAAACCAAGAT

CTTGGGGTTCTTGGGGAAACTGACATGCTGATTGGTTTTTGTACAGTGCCTGGCTGGTAA

CAGCAGGCCCTCAGGGGTGCGTTTCCTTCCTGGGGACTGGAGTGGGGGTTGCAGTAGACT

CTGGGAGGCCTCTCCAGCTGCAGAATCTCCCTCCTCCCTCCTCCTTTTTGTCTTCCTGAC

ACAAAACCCACCAGCTGCACTTCTTTGGGCTTGCAGTGGCTTTCAGTTACCAGAGCCACC

TGTTAAAACAAAAATGTGCCTAGGAAGAGCCTGCCTTACCCATTTTGACTCACATGGCAG

TTGGTGGTGGAGGGGAACAAAGGAGACTGAGTTTCATCGAAGCCTTTTGCTTCGGAGGAG

GAAGGGAGGATCAGAGAGAGGAAGTGGTCTGTGTTCACACAGGGAGGCAGGGGAGGCCAG

GCAGCTTCCCAATCCTGCATTCAACCTCAGGGTGGGCTTGACCTGGGTGGCTGGGGGCCC

TGTGATCCAGGAGAGACTTGTCCACCTGCTCAGGTGTCTTGAAGGGGTCCCTGTGGTACC

CCCTGGGCGGGGCAAGGTAGTAGGACCATGGTCTGGCTGGGGAGGTGGAGAGGAGCAGGC

TGTGGGCGCAGAGTGAGGTTGGAATCTGTATTTACCCAAGGTGTTGGGGGTAGGCTTGCC

CTCAGCCCTTAATGTTCTCAGGCCCCTGAGCAGTTGTGGGGGATAACCTCTGCACTCCTA

GTGACCAGGGAGCTAGAACAGCAAGGAATTTGAACTTGGACACCAGCTGGGGTCAGGCTC

TCTGGGTCTGAGTCCTGATTTCCCACTTTCCAGCTAGAGGAGCTTGAATGAGTCATTTAA

CTTCACGGTGCCTCAGTTTCCCCTCTCTAAAATGAGAATTATACCCATACCCACCTCTCA

AACACCAAGTGCAGGCCTGGCTCAGAGCAGGTGCTGCAGCAATAGCTGCCATTGGTCAGC

ATCATCATCATGGTTGGTAATGGTCCTACTTTGACTTTTGAGACAGAGTCTCACTCTGTC

GCCCAGGCTGGAGTGCAGTGGTGCAATCTCGGCTCACTACAACCTCTGCTCCCGGGTTCA

AGTGATTCTTCTGCCTCAGTCTCCCAAGTAGTTGGGATTACAGGTGTGCGCCACCATGCC

TGGCTAATTTTTGTGTTTTTAGTAGAGACAGGGTTTCACCATGTTGGCCATAACAATGGC

TGTCCTTGAAGTAGTCCTGGGTACCAGGTGCCTTCAGTGACTTTTTTTTTTTTTTTTTTT

TTGAGCTGGAGTCTTCCTCTGTCACCCAAGCTGGAGTGCAGTGGCACGATTTTGGCTCAC

TGCAACCTCTGCCTCCTGGGTTCATGCGATCCTCCTGCCTCAGCCTCCCAAGTAGCTGGG

ACTTGGGATACACTTGCCCCCGCTGGTCCTCCCTTCCACCTCTGTGAAGAGGAGGTCTCA

AACTCCTGGCCTCAAGTGATCCACCCACCTCAGCCTCCCAAAGTGCTGGGATTTCAAGAG

TGAGCCACCGCACCTGGCCCCTGTTTAGATGTTAGCATCAGTGACCCAGCACCTTGCTAT

GTGGCATGCCTGCAGGTCGACCATAGATAAAATAAAAGATTTTATTTAGTCTCCAGAAAA

AGGGGGGAATGAAAGACCCCACCTGTAGGTTTGGCAAGCTAGCGATATCAGTTCGCTTCT

CGCTTCTGTTCGCGCGCTTCTGCTCCCCGAGCTCAATAAAAGAGCCCACAACCCCTCACT

CGGGGCGCCAGTCCTCCGATTGACTGAGTCGCCCGGGTACCCGTGTATCCAATAAACCCT

CTTGCAGTTGCATCCGACTTGTGGTCTCGCTGTTCCTTGGGAGGGTCTCCTCTGAGTGAT

TGACTACCCGTCAGCGGGGGTCTTTCATTTGGGGGCTCGTCCGGGATCGCGGCCGCCACC

GCGGTGGAGCTCCAATTCGCCCTATAGTGAGTCGTATTACGCGCGCTCACTGGCCGTCGT

TTTACAACGTCGTGACTGGGAAAACCCTGGCGTTACCCAACTTAATCGCCTTGCAGCACA

TCCCCCTTTCGCCAGCTGGCGTAATAGCGAAGAGGCCCGCACCGATCGCCCTTCCCAACA

GTTGCGCAGCCTGAATGGCGAATGGGACGCGCCCTGTAGCGGCGCATTAAGCGCGGCGGG

TGTGGTGGTTACGCGCAGCGTGACCGCTACACTTGCCAGCGCCCTAGCGCCCGCTCCTTT

CGCTTTCTTCCCTTCCTTTCTCGCCACGTTCGCCGGCTTTCCCCGTCAAGCTCTAAATCG

GGGGCTCCCTTTAGGGTTCCGATTTAGTGCTTTACGGCACCTCGACCCCAAAAAACTTGA

TTAGGGTGATGGTTCACGTAGTGGGCCATCGCCCTGATAGACGGTTTTTCGCCCTTTGAC

GTTGGAGTCCACGTTCTTTAATAGTGGACTCTTGTTCCAAACTGGAACAACACTCAACCC

TATCTCGGTCTATTCTTTTGATTTATAAGGGATTTTGCCGATTTCGGCCTATTGGTTAAA

AAATGAGCTGATTTAACAAAAATTTAACGCGAATTTTAACAAAATATTAACGCTTACAAT

TTAGGTGGCACTTTTCGGGGAAATGTGCGCGGAACCCCTATTTGTTTATTTTTCTAAATA

CATTCAAATATGTATCCGCTCATGAGACAATAACCCTGATAAATGCTTCAATAATATTGA

AAAAGGAAGAGTATGAGTATTCAACATTTCCGTGTCGCCCTTATTCCCTTTTTTGCGGCA

TTTTGCCTTCCTGTTTTTGCTCACCCAGAAACGCTGGTGAAAGTAAAAGATGCTGAAGAT

CAGTTGGGTGCACGAGTGGGTTACATCGAACTGGATCTCAACAGCGGTAAGATCCTTGAG

AGTTTTCGCCCCGAAGAACGTTTTCCAATGATGAGCACTTTTAAAGTTCTGCTATGTGGC

GCGGTATTATCCCGTATTGACGCCGGGCAAGAGCAACTCGGTCGCCGCATACACTATTCT

CAGAATGACTTGGTTGAGTACTCACCAGTCACAGAAAAGCATCTTACGGATGGCATGACA

GTAAGAGAATTATGCAGTGCTGCCATAACCATGAGTGATAACACTGCGGCCAACTTACTT

CTGACAACGATCGGAGGACCGAAGGAGCTAACCGCTTTTTTGCACAACATGGGGGATCAT

GTAACTCGCCTTGATCGTTGGGAACCGGAGCTGAATGAAGCCATACCAAACGACGAGCGT

GACACCACGATGCCTGTAGCAATGGCAACAACGTTGCGCAAACTATTAACTGGCGAACTA

CTTACTCTAGCTTCCCGGCAACAATTAATAGACTGGATGGAGGCGGATAAAGTTGCAGGA

CCACTTCTGCGCTCGGCCCTTCCGGCTGGCTGGTTTATTGCTGATAAATCTGGAGCCGGT

GAGCGTGGGTCTCGCGGTATCATTGCAGCACTGGGGCCAGATGGTAAGCCCTCCCGTATC

GTAGTTATCTACACGACGGGGAGTCAGGCAACTATGGATGAACGAAATAGACAGATCGCT

GAGATAGGTGCCTCACTGATTAAGCATTGGTAACTGTCAGACCAAGTTTACTCATATATA

CTTTAGATTGATTTAAAACTTCATTTTTAATTTAAAAGGATCTAGGTGAAGATCCTTTTT

GATAATCTCATGACCAAAATCCCTTAACGTGAGTTTTCGTTCCACTGAGCGTCAGACCCC

GTAGAAAAGATCAAAGGATCTTCTTGAGATCCTTTTTTTCTGCGCGTAATCTGCTGCTTG

CAAACAAAAAAACCACCGCTACCAGCGGTGGTTTGTTTGCCGGATCAAGAGCTACCAACT

CTTTTTCCGAAGGTAACTGGCTTCAGCAGAGCGCAGATACCAAATACTGTCCTTCTAGTG

TAGCCGTAGTTAGGCCACCACTTCAAGAACTCTGTAGCACCGCCTACATACCTCGCTCTG

CTAATCCTGTTACCAGTGGCTGCTGCCAGTGGCGATAAGTCGTGTCTTACCGGGTTGGAC

TCAAGACGATAGTTACCGGATAAGGCGCAGCGGTCGGGCTGAACGGGGGGTTCGTGCACA

CAGCCCAGCTTGGAGCGAACGACCTACACCGAACTGAGATACCTACAGCGTGAGCTATGA

GAAAGCGCCACGCTTCCCGAAGGGAGAAAGGCGGACAGGTATCCGGTAAGCGGCAGGGTC

GGAACAGGAGAGCGCACGAGGGAGCTTCCAGGGGGAAACGCCTGGTATCTTTATAGTCCT

GTCGGGTTTCGCCACCTCTGACTTGAGCGTCGATTTTTGTGATGCTCGTCAGGGGGGCGG

AGCCTATGGAAAAACGCCAGCAACGCGGCCTTTTTACGGTTCCTGGCCTTTTGCTGGCCT

TTTGCTCACATGTTCTTTCCTGCGTTATCCCCTGATTCTGTGGATAACCGTATTACCGCC

TTTGAGTGAGCTGATACCGCTCGCCGCAGCCGAACGACCGAGCGCAGCGAGTCAGTGAGC

GAGGAAGCGGAAGAGCGCCCAATACGCAAACCGCCTCTCCCCGCGCGTTGGCCGATTCAT

TAATGCAGCTGGCACGACAGGTTTCCCGACTGGAAAGCGGGCAGTGAGCGCAACGCAATT

AATGTGAGTTAGCTCACTCATTAGGCACCCCAGGCTTTACACTTTATGCTTCCGGCTCGT

ATGTTGTGTGGAATTGTGAGCGGATAACAATTTCACACAGGAAACAGCTATGACCATGAT

TACGCCAAGCGCGCAATTAACCCTCACTAAAGGGAACAAAAGCTGGGTACCGGGCCCCCC

CTCGAGAGTC

**SHGAiL**

8395 bp

5’LTR CMV promoter 1-531

R region 532-590

U5 region 596-666

Packaging signal 667-1023

hGH pA 1647-1063 (reverse orientation)

eGFP gene 2384-1665 (reverse orientation)

ADA promoter 2840-2398 (reverse orientation)

ADA LCR 5132-2876 (reverse orientation)

3’LTR DU3 region 5213-5330

R region 5331-5397

U5 region 5398-5474

CGTTACATAACTTACGGTAAATGGCCCGCCTGGCTGACCGCCCAACGACCCCCGCCCATTGACGTCAATAATGACGTATGTTCCCATAGTAACGCCAATAGGGACTTTCCATTGACGTCAATGGGTGGAGTATTTACGGTAAACTGCCCACTTGGCAGTACATCAAGTGTATCATATGCCAAGTACGCCCCCTATTGACGTCAATGACGGTAAATGGCCCGCCTGGCATTATGCCCAGTACATGACCTTATGGGACTTTCCTACTTGGCAGTACATCTACGTATTAGTCATCGCTATTACCATGGTGATGCGGTTTTGGCAGTACATCAATGGGCGTGGATAGCGGTTTGACTCACGGGGATTTCCAAGTCTCCACCCCATTGACGTCAATGGGAGTTTGTTTTGGCACCAAAATCAACGGGACTTTCCAAAATGTCGTAACAACTCCGCCCCATTGACGCAAATGGGCGGTAGGCGTGTACGGTGGGAGGTCTATATAAGCAGAGCTCGTTTAGTGAACCGCGCCAGTCTTCCGATAGACTGCGTCGCCCGGGTACCCGTATTCCCAATAAAGCCTCTTGCTGTTTGCATCCGAATCGTGGTCTCGCTGTTCCTTGGGAGGGTCTCCTCTGAGTGATTGACTACCCACGACGGGGGTCTTTCATTTGGGGGCTCGTCCGGGATTTGGAGACCCCTGCCCAGGGACCACCGACCCACCACCGGGAGGTAAGCTGGCCAGCAACTTATCTGTGTCTGTCCGATTGTCTAGTGTCTATGTTTGATGTTATGCGCCTGCGTCTGTACTAGTTAGCTAACTAGCTCTGTATCTGGCGGACCCGTGGTGGAACTGACGAGTTCTGAACACCCGGCCGCAACCCTGGGAGACGTCCCAGGGACTTTGGGGGCCGTTTTTGTGGCCCGACCTGAGGAAGGGAGTCGATGTGGAATCCGACCCCGTCAGGATATGTGGTTCTGGTAGGAGACGAGAACCTAAAACAGTTCCCGCCTCCGTCTGAATTTTTGCTAAGCTCTAGAGTCGACACGCGTGCGATCGCTCGCGATAGCTTGCCAAGCAAACAACTCAAATGTCCAACTGGCTGGGCATGGCCAGGTAGCCCATGCTGTGTCTGGACGTCCTTCTGCTGGTATAATTATTTTAAAATCACAGGACAGGGAAGGGCCCAGTGGCTCATACCTGTAATCCCAGCAATTTGGGAGGCCGAGGCGGGCGGATTACCTGAGGTCAGGAGATGGAGACCAGACTGGCCAATATGGTGAAACCCCGTCTCTACCAAAAATACAAAAATTAGCTGAGCCTGGTCGTGCATGCCTGGAATCCCAACTATTCGGGAGACTGAGGCAGGAGAATCGCTTGAACCCAGGAGGCGGAGATTGCAGCGAGCCAAGATCGTGCCACTGCACTCCAGCTTGGTTCCCAATAGACCCCGCAGGCCCTACAGGTTGCCTTCCCAACTTGCCCCTTGCTCCATACCACCCCCCTCCACCCCATAATATTATAGAAGGACACCTAGTCAGACAAAATGATGCAACTTAATTTTATTAGGACAAGGCTGGTGGGCACTGGAGTGGCAACTTCCAGGGCCAGGAGAGGCACTGGGGAGGGGTCACAGGGATGCCACGTTAACTCTAGAGTCGCGGCCGCTTTACTTGTACAGCTCGTCCATGCCGAGAGTGATCCCGGCGGCGGTCACGAACTCCAGCAGGACCATGTGATCGCGCTTCTCGTTGGGGTCTTTGCTCAGGGCGGACTGGGTGCTCAGGTAGTGGTTGTCGGGCAGCAGCACGGGGCCGTCGCCGATGGGGGTGTTCTGCTGGTAGTGGTCGGCGAGCTGCACGCTGCCGTCCTCGATGTTGTGGCGGATCTTGAAGTTCACCTTGATGCCGTTCTTCTGCTTGTCGGCCATGATATAGACGTTGTGGCTGTTGTAGTTGTACTCCAGCTTGTGCCCCAGGATGTTGCCGTCCTCCTTGAAGTCGATGCCCTTCAGCTCGATGCGGTTCACCAGGGTGTCGCCCTCGAACTTCACCTCGGCGCGGGTCTTGTAGTTGCCGTCGTCCTTGAAGAAGATGGTGCGCTCCTGGACGTAGCCTTCGGGCATGGCGGACTTGAAGAAGTCGTGCTGCTTCATGTGGTCGGGGTAGCGGCTGAAGCACTGCACGCCGTAGGTCAGGGTGGTCACGAGGGTGGGCCAGGGCACGGGCAGCTTGCCGGTGGTGCAGATGAACTTCAGGGTCAGCTTGCCGTAGGTGGCATCGCCCTCGCCCTCGCCGGACACGCTGAACTTGTGGCCGTTTACGTCGCCGTCCAGCTCGACCAGGATGGGCACCACCCCGGTGAACAGCTCCTCGCCCTTGCTCACCATGGTGGCGACCGGTGGTGCCCTCGTGCGCCCCGGCGCTGCTCCCTCCGCCGCCGCTCGGTGGGTCTCTGCCGGCTCGGTGGCCGCTCGGCTTTCCCTGGGGCCAGCGGTGGCCGCGGCCGGCCACGCTCTTCTTAACGGGCCGGGCCCCGCCTCCCGCCCCGCCCGCCGCCGTTCCCGGGGCCCGCCCTCTCGCCGGAGCCCCCGCCCCGCCCGCCGGCCTGGATCGCGCATTTCCTGGAATTCTGGACCCGGCGTCCTCGCCCGCCGACCCGAAAACTACGGCCCCGCGGCGCCCGACCGGACCCACCTGTGGGCGCGCGCTCCAGCCGCCTCGCTGGGCGCTGCCTTCCCCCCGGCTCCTCCAGCAACTTTCAAGCGTCCCCCTGCAGTCTGGGCGCTGCTGTGCGCCAGGCCCAAGCTAGAAGCGGGGACCCCGGGAGCATCGCAAGCCTCGAGGGCGCGCCTTAATTAACACCGGCGAAGCTTGGATCCATGCCACATAGCAAGGTGCTGGGTCACTGATGCTAACATCTAAACAGGGGCCAGGTGCGGTGGCTCACTCTTGAAATCCCAGCACTTTGGGAGGCTGAGGTGGGTGGATCACTTGAGGCCAGGAGTTTGAGACCTCCTCTTCACAGAGGTGGAAGGGAGGACCAGCGGGGGCAAGTGTATCCCAAGTCCCAGCTACTTGGGAGGCTGAGGCAGGAGGATCGCATGAACCCAGGAGGCAGAGGTTGCAGTGAGCCAAAATCGTGCCACTGCACTCCAGCTTGGGTGACAGAGGAAGACTCCAGCTCAAAAAAAAAAAAAAAAAAAAAGTCACTGAAGGCACCTGGTACCCAGGACTACTTCAAGGACAGCCATTGTTATGGCCAACATGGTGAAACCCTGTCTCTACTAAAAACACAAAAATTAGCCAGGCATGGTGGCGCACACCTGTAATCCCAACTACTTGGGAGACTGAGGCAGAAGAATCACTTGAACCCGGGAGGCAGAGGTTGTAGTGAGCCGAGATTGCACCACTGCACTCCAGCCTGGGCGACAGAGTGAGACTCTGTCTCAAAAGTCAAAGTAGGACCATTACCACCATGATGATGATGCTGACCAATGGCAGCTATTGCTGCAGCACCTGCTCTGAGCCAGGCCTGCACTTGGTGTTTGAGAGGTGGGTATGGGTATAATTCTCATTTTAGAGAGGGGAAACTGAGGCACCGTGAAGTTAAATGACTCATTCAAGCTCCTCTAGCTGGAAAGTGGGAAATCAGGACTCAGACCCAGAGAGCCTGACCCCAGCTGGTGTCCAAGTTCAAATTCCTTGCTGTTCTAGCTCCCTGGTCACTAGGAGTGCAGAGGTTATCCCCCACAACTGCTCAGGGGCCTGAGAACATTAAGGGCTGAGGGCAAGCCTACCCCCAACACCTTGGGTAAATACAGATTCCAACCTCACTCTGCGCCCACAGCCTGCTCCTCTCCACCTCCCCAGCCAGACCATGGTCCTACTACCTTGCCCCGCCCAGGGGGTACCACAGGGACCCCTTCAAGACACCTGAGCAGGTGGACAAGTCTCTCCTGGATCACAGGGCCCCCAGCCACCCAGGTCAAGCCCACCCTGAGGTTGAATGCAGGATTGGGAAGCTGCCTGGCCTCCCCTGCCTCCCTGTGTGAACACAGACCACTTCCTCTCTCTGATCCTCCCTTCCTCCTCCGAAGCAAAAGGCTTCGATGAAACTCAGTCTCCTTTGTTCCCCTCCACCACCAACTGCCATGTGAGTCAAAATGGGTAAGGCAGGCTCTTCCTAGGCACATTTTTGTTTTAACAGGTGGCTCTGGTAACTGAAAGCCACTGCAAGCCCAAAGAAGTGCAGCTGGTGGGTTTTGTGTCAGGAAGACAAAAAGGAGGAGGGAGGAGGGAGATTCTGCAGCTGGAGAGGCCTCCCAGAGTCTACTGCAACCCCCACTCCAGTCCCCAGGAAGGAAACGCACCCCTGAGGGCCTGCTGTTACCAGCCAGGCACTGTACAAAAACCAATCAGCATGTCAGTTTCCCCAAGAACCCCAAGATCTTGGTTTTGTCACCCCATTTTACAGAGGGGGAAGCTGAGTTTCAGGAGTTGTCTACCTGAGGTACCCTAGCTGCTAAGCGGCAGGCAGGAATTCAACCCCAGTACATCTGAAGCCCAGGGCTAATGCACTGGCCCACCCTGCCACCCTGATTCTAACTCTTCCCCCCACACTGCCCCCTCTTTTTTGCATAAAGTGGAATTGGCAAATGAGCAAAGCAATGAACAGCTTCTACTTGAGTGCAGGCAGATCTGGGTTCAAGTCTAGCTCTGCTGCTTGCCAGCTGTGTGACTTTGGCAAGTTCCCTCCCCTCTCTGAGTCTCAGTTTCCTCATAATAAGTGAAGTTAATAATAGGACAGGACCTTCCTCAGAGGGCTGACATAAACATTAAATGAGTCACTGTATGCAAAGCAGGACAGCGCCTCGTGCAGAGTAGACATTCAAATATGAGACAGAAAAGGCACATTGCTGATTTAGCCCCAATTCCCCTCTGCATCAGAGAGGGACAGTTTAGAAACTGCTGTCCCTTGGCCGGGTGCGGTGGCTCATGCCTGTAATCCCGGCACTTTGGGAGGCTGAGGTGGGCGGATCACCTGAAGTCGGGAGTTCGAGACCAGCCTGGCCAACAAGGAGAAACCCCGTCTCTACTAAAAATACAAAAAATTAGCCGGGCATGGTGGTGCATGCCTTAAGTATCGATACACGTGGTTTAAACGTCGACCATAGATAAAATAAAAGATTTTATTTAGTCTCCAGAAAAAGGGGGGAATGAAAGACCCCACCTGTAGGTTTGGCAAGCTAGCGATATCAGTTCGCTTCTCGCTTCTGTTCGCGCGCTTCTGCTCCCCGAGCTCAATAAAAGAGCCCACAACCCCTCACTCGGGGCGCCAGTCCTCCGATTGACTGAGTCGCCCGGGTACCCGTGTATCCAATAAACCCTCTTGCAGTTGCATCCGACTTGTGGTCTCGCTGTTCCTTGGGAGGGTCTCCTCTGAGTGATTGACTACCCGTCAGCGGGGGTCTTTCATTTGGGGGCTCGTCCGGGATCGCGGCCGCCACCGCGGTGGAGCTCCAATTCGCCCTATAGTGAGTCGTATTACGCGCGCTCACTGGCCGTCGTTTTACAACGTCGTGACTGGGAAAACCCTGGCGTTACCCAACTTAATCGCCTTGCAGCACATCCCCCTTTCGCCAGCTGGCGTAATAGCGAAGAGGCCCGCACCGATCGCCCTTCCCAACAGTTGCGCAGCCTGAATGGCGAATGGGACGCGCCCTGTAGCGGCGCATTAAGCGCGGCGGGTGTGGTGGTTACGCGCAGCGTGACCGCTACACTTGCCAGCGCCCTAGCGCCCGCTCCTTTCGCTTTCTTCCCTTCCTTTCTCGCCACGTTCGCCGGCTTTCCCCGTCAAGCTCTAAATCGGGGGCTCCCTTTAGGGTTCCGATTTAGTGCTTTACGGCACCTCGACCCCAAAAAACTTGATTAGGGTGATGGTTCACGTAGTGGGCCATCGCCCTGATAGACGGTTTTTCGCCCTTTGACGTTGGAGTCCACGTTCTTTAATAGTGGACTCTTGTTCCAAACTGGAACAACACTCAACCCTATCTCGGTCTATTCTTTTGATTTATAAGGGATTTTGCCGATTTCGGCCTATTGGTTAAAAAATGAGCTGATTTAACAAAAATTTAACGCGAATTTTAACAAAATATTAACGCTTACAATTTAGGTGGCACTTTTCGGGGAAATGTGCGCGGAACCCCTATTTGTTTATTTTTCTAAATACATTCAAATATGTATCCGCTCATGAGACAATAACCCTGATAAATGCTTCAATAATATTGAAAAAGGAAGAGTATGAGTATTCAACATTTCCGTGTCGCCCTTATTCCCTTTTTTGCGGCATTTTGCCTTCCTGTTTTTGCTCACCCAGAAACGCTGGTGAAAGTAAAAGATGCTGAAGATCAGTTGGGTGCACGAGTGGGTTACATCGAACTGGATCTCAACAGCGGTAAGATCCTTGAGAGTTTTCGCCCCGAAGAACGTTTTCCAATGATGAGCACTTTTAAAGTTCTGCTATGTGGCGCGGTATTATCCCGTATTGACGCCGGGCAAGAGCAACTCGGTCGCCGCATACACTATTCTCAGAATGACTTGGTTGAGTACTCACCAGTCACAGAAAAGCATCTTACGGATGGCATGACAGTAAGAGAATTATGCAGTGCTGCCATAACCATGAGTGATAACACTGCGGCCAACTTACTTCTGACAACGATCGGAGGACCGAAGGAGCTAACCGCTTTTTTGCACAACATGGGGGATCATGTAACTCGCCTTGATCGTTGGGAACCGGAGCTGAATGAAGCCATACCAAACGACGAGCGTGACACCACGATGCCTGTAGCAATGGCAACAACGTTGCGCAAACTATTAACTGGCGAACTACTTACTCTAGCTTCCCGGCAACAATTAATAGACTGGATGGAGGCGGATAAAGTTGCAGGACCACTTCTGCGCTCGGCCCTTCCGGCTGGCTGGTTTATTGCTGATAAATCTGGAGCCGGTGAGCGTGGGTCTCGCGGTATCATTGCAGCACTGGGGCCAGATGGTAAGCCCTCCCGTATCGTAGTTATCTACACGACGGGGAGTCAGGCAACTATGGATGAACGAAATAGACAGATCGCTGAGATAGGTGCCTCACTGATTAAGCATTGGTAACTGTCAGACCAAGTTTACTCATATATACTTTAGATTGATTTAAAACTTCATTTTTAATTTAAAAGGATCTAGGTGAAGATCCTTTTTGATAATCTCATGACCAAAATCCCTTAACGTGAGTTTTCGTTCCACTGAGCGTCAGACCCCGTAGAAAAGATCAAAGGATCTTCTTGAGATCCTTTTTTTCTGCGCGTAATCTGCTGCTTGCAAACAAAAAAACCACCGCTACCAGCGGTGGTTTGTTTGCCGGATCAAGAGCTACCAACTCTTTTTCCGAAGGTAACTGGCTTCAGCAGAGCGCAGATACCAAATACTGTCCTTCTAGTGTAGCCGTAGTTAGGCCACCACTTCAAGAACTCTGTAGCACCGCCTACATACCTCGCTCTGCTAATCCTGTTACCAGTGGCTGCTGCCAGTGGCGATAAGTCGTGTCTTACCGGGTTGGACTCAAGACGATAGTTACCGGATAAGGCGCAGCGGTCGGGCTGAACGGGGGGTTCGTGCACACAGCCCAGCTTGGAGCGAACGACCTACACCGAACTGAGATACCTACAGCGTGAGCTATGAGAAAGCGCCACGCTTCCCGAAGGGAGAAAGGCGGACAGGTATCCGGTAAGCGGCAGGGTCGGAACAGGAGAGCGCACGAGGGAGCTTCCAGGGGGAAACGCCTGGTATCTTTATAGTCCTGTCGGGTTTCGCCACCTCTGACTTGAGCGTCGATTTTTGTGATGCTCGTCAGGGGGGCGGAGCCTATGGAAAAACGCCAGCAACGCGGCCTTTTTACGGTTCCTGGCCTTTTGCTGGCCTTTTGCTCACATGTTCTTTCCTGCGTTATCCCCTGATTCTGTGGATAACCGTATTACCGCCTTTGAGTGAGCTGATACCGCTCGCCGCAGCCGAACGACCGAGCGCAGCGAGTCAGTGAGCGAGGAAGCGGAAGAGCGCCCAATACGCAAACCGCCTCTCCCCGCGCGTTGGCCGATTCATTAATGCAGCTGGCACGACAGGTTTCCCGACTGGAAAGCGGGCAGTGAGCGCAACGCAATTAATGTGAGTTAGCTCACTCATTAGGCACCCCAGGCTTTACACTTTATGCTTCCGGCTCGTATGTTGTGTGGAATTGTGAGCGGATAACAATTTCACACAGGAAACAGCTATGACCATGATTACGCCAAGCGCGCAATTAACCCTCACTAAAGGGAACAAAAGCTGGGTACCGGGCCCCCCCTCGAGAGTC

**SHGI7AL**

8499 bp

5’LTR CMV promoter 1-531

R region 532-590

U5 region 596-666

Packaging signal 667-1023

hGH pA 1647-1063 (reverse orientation)

eGFP gene 2384-1665 (reverse orientation)

Intron 7 2483-2408 (reverse orientation)

ADA promoter 2944-2502 (reverse orientation)

ADA LCR 2973-5229 (reverse orientation)

3’LTR DU3 region 5317-5434

R region 5435-5501

U5 region 5502-5578

CGTTACATAACTTACGGTAAATGGCCCGCCTGGCTGACCGCCCAACGACCCCCGCCCATTGACGTCAATAATGACGTATGTTCCCATAGTAACGCCAATAGGGACTTTCCATTGACGTCAATGGGTGGAGTATTTACGGTAAACTGCCCACTTGGCAGTACATCAAGTGTATCATATGCCAAGTACGCCCCCTATTGACGTCAATGACGGTAAATGGCCCGCCTGGCATTATGCCCAGTACATGACCTTATGGGACTTTCCTACTTGGCAGTACATCTACGTATTAGTCATCGCTATTACCATGGTGATGCGGTTTTGGCAGTACATCAATGGGCGTGGATAGCGGTTTGACTCACGGGGATTTCCAAGTCTCCACCCCATTGACGTCAATGGGAGTTTGTTTTGGCACCAAAATCAACGGGACTTTCCAAAATGTCGTAACAACTCCGCCCCATTGACGCAAATGGGCGGTAGGCGTGTACGGTGGGAGGTCTATATAAGCAGAGCTCGTTTAGTGAACCGCGCCAGTCTTCCGATAGACTGCGTCGCCCGGGTACCCGTATTCCCAATAAAGCCTCTTGCTGTTTGCATCCGAATCGTGGTCTCGCTGTTCCTTGGGAGGGTCTCCTCTGAGTGATTGACTACCCACGACGGGGGTCTTTCATTTGGGGGCTCGTCCGGGATTTGGAGACCCCTGCCCAGGGACCACCGACCCACCACCGGGAGGTAAGCTGGCCAGCAACTTATCTGTGTCTGTCCGATTGTCTAGTGTCTATGTTTGATGTTATGCGCCTGCGTCTGTACTAGTTAGCTAACTAGCTCTGTATCTGGCGGACCCGTGGTGGAACTGACGAGTTCTGAACACCCGGCCGCAACCCTGGGAGACGTCCCAGGGACTTTGGGGGCCGTTTTTGTGGCCCGACCTGAGGAAGGGAGTCGATGTGGAATCCGACCCCGTCAGGATATGTGGTTCTGGTAGGAGACGAGAACCTAAAACAGTTCCCGCCTCCGTCTGAATTTTTGCTAAGCTCTAGAGTCGACACGCGTGCGATCGCTCGCGATAGCTTGCCAAGCAAACAACTCAAATGTCCAACTGGCTGGGCATGGCCAGGTAGCCCATGCTGTGTCTGGACGTCCTTCTGCTGGTATAATTATTTTAAAATCACAGGACAGGGAAGGGCCCAGTGGCTCATACCTGTAATCCCAGCAATTTGGGAGGCCGAGGCGGGCGGATTACCTGAGGTCAGGAGATGGAGACCAGACTGGCCAATATGGTGAAACCCCGTCTCTACCAAAAATACAAAAATTAGCTGAGCCTGGTCGTGCATGCCTGGAATCCCAACTATTCGGGAGACTGAGGCAGGAGAATCGCTTGAACCCAGGAGGCGGAGATTGCAGCGAGCCAAGATCGTGCCACTGCACTCCAGCCTGGTTCCCAATAGACCCTGAAGGCCCTACAGGTTGTCTTCCCAACCTGCCCCTTGCTCCATACCACCCGCCTCCACCCCATAATATTATACAAGGACACCTAGTCAAACAAAATGATGCAACTTAATTTTATTAGGACAAGGCTGGTGGGCACTGGAGTGGCAACTTCCAGGGCCAGGAGAGGCACTGGGGAGGGGTCACAGGGATGCCACGTTAACTCTAGAGTCGCGGCCGCTTTACTTGTACAGCTCGTCCATGCCGAGAGTGATCCCGGCGGCGGTCACGAACTCCAGCAGGACCATGTGATCGCGCTTCTCGTTGGGGTCTTTGCTCAGGGCGGACTGGGTGCTCAGGTAGTGGTTGTCGGGCAGCAGCACGGGGCCGTCGCCGATGGGGGTGTTCTGCTGGTAGTGGTCGGCGAGCTGCACGCTGCCGTCCTCGATGTTGTGGCGGATCTTGAAGTTCACCTTGATGCCGTTCTTCTGCTTGTCGGCCATGATATAGACGTTGTGGCTGTTGTAGTTGTACTCCAGCTTGTGCCCCAGGATGTTGCCGTCCTCCTTGAAGTCGATGCCCTTCAGCTCGATGCGGTTCACCAGGGTGTCGCCCTCGAACTTCACCTCGGCGCGGGTCTTGTAGTTGCCGTCGTCCTTGAAGAAGATGGTGCGCTCCTGGACGTAGCCTTCGGGCATGGCGGACTTGAAGAAGTCGTGCTGCTTCATGTGGTCGGGGTAGCGGCTGAAGCACTGCACGCCGTAGGTCAGGGTGGTCACGAGGGTGGGCCAGGGCACGGGCAGCTTGCCGGTGGTGCAGATGAACTTCAGGGTCAGCTTGCCGTAGGTGGCATCGCCCTCGCCCTCGCCGGACACGCTGAACTTGTGGCCGTTTACGTCGCCGTCCAGCTCGACCAGGATGGGCACCACCCCGGTGAACAGCTCCTCGCCCTTGCTCACCATGGTGGCGACCGGATCTCCACAGCCTGTAGAGAAGCAGAATAGAGCCAAGTATGGGAGGAGGCAGTGAGGAGGGACCCCATGGCCAGCCCAGGCCCTCACCTCTTTTACTGATCCGGTGGTGCCCTCGTGCGCCCCGGCGCTGCTCCCTCCGCCGCCGCTCGGTGGGTCTCTGCCGGCTCGGTGGCCGCTCGGCTTTCCCTGGGGCCAGCGGTGGCCGCGGCCGGCCACGCTCTTCTTAACGGGCCGGGCCCCGCCTCCCGCCCCGCCCGCCGCCGTTCCCGGGGCCCGCCCTCTCGCCGGAGCCCCCGCCCCGCCCGCCGGCCTGGATCGCGCATTTCCTGGAATTCTGGACCCGGCGTCCTCGCCCGCCGACCCGAAAACTACGGCCCCGCGGCGCCCGACCGGACCCACCTGTGGGCGCGCGCTCCAGCCGCCTCGCTGGGCGCTGCCTTCCCCCCGGCTCCTCCAGCAACTTTCAAGCGTCCCCCTGCAGTCTGGGCGCTGCTGTGCGCCAGGCCCAAGCTAGAAGCGGGGACCCCGGGAGCATCGCAAGCCTCGAGGGCGCGCCCACGTGTATCGATACTTAAGGCATGCACCACCATGCCCGGCTAATTTTTTGTATTTTTAGTAGAGACGGGGTTTCTCCTTGTTGGCCAGGCTGGTCTCGAACTCCCGACTTCAGGTGATCCGCCCACCTCAGCCTCCCAAAGTGCCGGGATTACAGGCATGAGCCACCGCACCCGGCCAAGGGACAGCAGTTTCTAAACTGTCCCTCTCTGATGCAGAGGGGAATTGGGGCTAAATCAGCAATGTGCCTTTTCTGTCTCATATTTGAATGTCTACTCTGCACGAGGCGCTGTCCTGCTTTGCATACAGTGACTCATTTAATGTTTATGTCAGCCCTCTGAGGAAGGTCCTGTCCTATTATTAACTTCACTTATTATGAGGAAACTGAGACTCAGAGAGGGGAGGGAACTTGCCAAAGTCACACAGCTGGCAAGCAGCAGAGCTAGACTTGAACCCAGATCTGCCTGCACTCAAGTAGAAGCTGTTCATTGCTTTGCTCATTTGCCAATTCCACTTTATGCAAAAAAGAGGGGGCAGTGTGGGGGGAAGAGTTAGAATCAGGGTGGCAGGGTGGGCCAGTGCATTAGCCCTGGGCTTCAGATGTACTGGGGTTGAATTCCTGCCTGCCGCTTAGCAGCTAGGGTACCTCAGGTAGACAACTCCTGAAACTCAGCTTCCCCCTCTGTAAAATGGGGTGACAAAACCAAGATCTTGGGGTTCTTGGGGAAACTGACATGCTGATTGGTTTTTGTACAGTGCCTGGCTGGTAACAGCAGGCCCTCAGGGGTGCGTTTCCTTCCTGGGGACTGGAGTGGGGGTTGCAGTAGACTCTGGGAGGCCTCTCCAGCTGCAGAATCTCCCTCCTCCCTCCTCCTTTTTGTCTTCCTGACACAAAACCCACCAGCTGCACTTCTTTGGGCTTGCAGTGGCTTTCAGTTACCAGAGCCACCTGTTAAAACAAAAATGTGCCTAGGAAGAGCCTGCCTTACCCATTTTGACTCACATGGCAGTTGGTGGTGGAGGGGAACAAAGGAGACTGAGTTTCATCGAAGCCTTTTGCTTCGGAGGAGGAAGGGAGGATCAGAGAGAGGAAGTGGTCTGTGTTCACACAGGGAGGCAGGGGAGGCCAGGCAGCTTCCCAATCCTGCATTCAACCTCAGGGTGGGCTTGACCTGGGTGGCTGGGGGCCCTGTGATCCAGGAGAGACTTGTCCACCTGCTCAGGTGTCTTGAAGGGGTCCCTGTGGTACCCCCTGGGCGGGGCAAGGTAGTAGGACCATGGTCTGGCTGGGGAGGTGGAGAGGAGCAGGCTGTGGGCGCAGAGTGAGGTTGGAATCTGTATTTACCCAAGGTGTTGGGGGTAGGCTTGCCCTCAGCCCTTAATGTTCTCAGGCCCCTGAGCAGTTGTGGGGGATAACCTCTGCACTCCTAGTGACCAGGGAGCTAGAACAGCAAGGAATTTGAACTTGGACACCAGCTGGGGTCAGGCTCTCTGGGTCTGAGTCCTGATTTCCCACTTTCCAGCTAGAGGAGCTTGAATGAGTCATTTAACTTCACGGTGCCTCAGTTTCCCCTCTCTAAAATGAGAATTATACCCATACCCACCTCTCAAACACCAAGTGCAGGCCTGGCTCAGAGCAGGTGCTGCAGCAATAGCTGCCATTGGTCAGCATCATCATCATGGTGGTAATGGTCCTACTTTGACTTTTGAGACAGAGTCTCACTCTGTCGCCCAGGCTGGAGTGCAGTGGTGCAATCTCGGCTCACTACAACCTCTGCCTCCCGGGTTCAAGTGATTCTTCTGCCTCAGTCTCCCAAGTAGTTGGGATTACAGGTGTGCGCCACCATGCCTGGCTAATTTTTGTGTTTTTAGTAGAGACAGGGTTTCACCATGTTGGCCATAACAATGGCTGTCCTTGAAGTAGTCCTGGGTACCAGGTGCCTTCAGTGACTTTTTTTTTTTTTTTTTTTTTGAGCTGGAGTCTTCCTCTGTCACCCAAGCTGGAGTGCAGTGGCACGATTTTGGCTCACTGCAACCTCTGCCTCCTGGGTTCATGCGATCCTCCTGCCTCAGCCTCCCAAGTAGCTGGGACTTGGGATACACTTGCCCCCGCTGGTCCTCCCTTCCACCTCTGTGAAGAGGAGGTCTCAAACTCCTGGCCTCAAGTGATCCACCCACCTCAGCCTCCCAAAGTGCTGGGATTTCAAGAGTGAGCCACCGCACCTGGCCCCTGTTTAGATGTTAGCATCAGTGACCCAGCACCTTGCTATGTGGCATGGATCCAAGCTTCGCCGGTGTTAATTAAGTTTAAACGTCGACCATAGATAAAATAAAAGATTTTATTTAGTCTCCAGAAAAAGGGGGGAATGAAAGACCCCACCTGTAGGTTTGGCAAGCTAGCGATATCAGTTCGCTTCTCGCTTCTGTTCGCGCGCTTCTGCTCCCCGAGCTCAATAAAAGAGCCCACAACCCCTCACTCGGGGCGCCAGTCCTCCGATTGACTGAGTCGCCCGGGTACCCGTGTATCCAATAAACCCTCTTGCAGTTGCATCCGACTTGTGGTCTCGCTGTTCCTTGGGAGGGTCTCCTCTGAGTGATTGACTACCCGTCAGCGGGGGTCTTTCATTTGGGGGCTCGTCCGGGATCGCGGCCGCCACCGCGGTGGAGCTCCAATTCGCCCTATAGTGAGTCGTATTACGCGCGCTCACTGGCCGTCGTTTTACAACGTCGTGACTGGGAAAACCCTGGCGTTACCCAACTTAATCGCCTTGCAGCACATCCCCCTTTCGCCAGCTGGCGTAATAGCGAAGAGGCCCGCACCGATCGCCCTTCCCAACAGTTGCGCAGCCTGAATGGCGAATGGGACGCGCCCTGTAGCGGCGCATTAAGCGCGGCGGGTGTGGTGGTTACGCGCAGCGTGACCGCTACACTTGCCAGCGCCCTAGCGCCCGCTCCTTTCGCTTTCTTCCCTTCCTTTCTCGCCACGTTCGCCGGCTTTCCCCGTCAAGCTCTAAATCGGGGGCTCCCTTTAGGGTTCCGATTTAGTGCTTTACGGCACCTCGACCCCAAAAAACTTGATTAGGGTGATGGTTCACGTAGTGGGCCATCGCCCTGATAGACGGTTTTTCGCCCTTTGACGTTGGAGTCCACGTTCTTTAATAGTGGACTCTTGTTCCAAACTGGAACAACACTCAACCCTATCTCGGTCTATTCTTTTGATTTATAAGGGATTTTGCCGATTTCGGCCTATTGGTTAAAAAATGAGCTGATTTAACAAAAATTTAACGCGAATTTTAACAAAATATTAACGCTTACAATTTAGGTGGCACTTTTCGGGGAAATGTGCGCGGAACCCCTATTTGTTTATTTTTCTAAATACATTCAAATATGTATCCGCTCATGAGACAATAACCCTGATAAATGCTTCAATAATATTGAAAAAGGAAGAGTATGAGTATTCAACATTTCCGTGTCGCCCTTATTCCCTTTTTTGCGGCATTTTGCCTTCCTGTTTTTGCTCACCCAGAAACGCTGGTGAAAGTAAAAGATGCTGAAGATCAGTTGGGTGCACGAGTGGGTTACATCGAACTGGATCTCAACAGCGGTAAGATCCTTGAGAGTTTTCGCCCCGAAGAACGTTTTCCAATGATGAGCACTTTTAAAGTTCTGCTATGTGGCGCGGTATTATCCCGTATTGACGCCGGGCAAGAGCAACTCGGTCGCCGCATACACTATTCTCAGAATGACTTGGTTGAGTACTCACCAGTCACAGAAAAGCATCTTACGGATGGCATGACAGTAAGAGAATTATGCAGTGCTGCCATAACCATGAGTGATAACACTGCGGCCAACTTACTTCTGACAACGATCGGAGGACCGAAGGAGCTAACCGCTTTTTTGCACAACATGGGGGATCATGTAACTCGCCTTGATCGTTGGGAACCGGAGCTGAATGAAGCCATACCAAACGACGAGCGTGACACCACGATGCCTGTAGCAATGGCAACAACGTTGCGCAAACTATTAACTGGCGAACTACTTACTCTAGCTTCCCGGCAACAATTAATAGACTGGATGGAGGCGGATAAAGTTGCAGGACCACTTCTGCGCTCGGCCCTTCCGGCTGGCTGGTTTATTGCTGATAAATCTGGAGCCGGTGAGCGTGGGTCTCGCGGTATCATTGCAGCACTGGGGCCAGATGGTAAGCCCTCCCGTATCGTAGTTATCTACACGACGGGGAGTCAGGCAACTATGGATGAACGAAATAGACAGATCGCTGAGATAGGTGCCTCACTGATTAAGCATTGGTAACTGTCAGACCAAGTTTACTCATATATACTTTAGATTGATTTAAAACTTCATTTTTAATTTAAAAGGATCTAGGTGAAGATCCTTTTTGATAATCTCATGACCAAAATCCCTTAACGTGAGTTTTCGTTCCACTGAGCGTCAGACCCCGTAGAAAAGATCAAAGGATCTTCTTGAGATCCTTTTTTTCTGCGCGTAATCTGCTGCTTGCAAACAAAAAAACCACCGCTACCAGCGGTGGTTTGTTTGCCGGATCAAGAGCTACCAACTCTTTTTCCGAAGGTAACTGGCTTCAGCAGAGCGCAGATACCAAATACTGTCCTTCTAGTGTAGCCGTAGTTAGGCCACCACTTCAAGAACTCTGTAGCACCGCCTACATACCTCGCTCTGCTAATCCTGTTACCAGTGGCTGCTGCCAGTGGCGATAAGTCGTGTCTTACCGGGTTGGACTCAAGACGATAGTTACCGGATAAGGCGCAGCGGTCGGGCTGAACGGGGGGTTCGTGCACACAGCCCAGCTTGGAGCGAACGACCTACACCGAACTGAGATACCTACAGCGTGAGCTATGAGAAAGCGCCACGCTTCCCGAAGGGAGAAAGGCGGACAGGTATCCGGTAAGCGGCAGGGTCGGAACAGGAGAGCGCACGAGGGAGCTTCCAGGGGGAAACGCCTGGTATCTTTATAGTCCTGTCGGGTTTCGCCACCTCTGACTTGAGCGTCGATTTTTGTGATGCTCGTCAGGGGGGCGGAGCCTATGGAAAAACGCCAGCAACGCGGCCTTTTTACGGTTCCTGGCCTTTTGCTGGCCTTTTGCTCACATGTTCTTTCCTGCGTTATCCCCTGATTCTGTGGATAACCGTATTACCGCCTTTGAGTGAGCTGATACCGCTCGCCGCAGCCGAACGACCGAGCGCAGCGAGTCAGTGAGCGAGGAAGCGGAAGAGCGCCCAATACGCAAACCGCCTCTCCCCGCGCGTTGGCCGATTCATTAATGCAGCTGGCACGACAGGTTTCCCGACTGGAAAGCGGGCAGTGAGCGCAACGCAATTAATGTGAGTTAGCTCACTCATTAGGCACCCCAGGCTTTACACTTTATGCTTCCGGCTCGTATGTTGTGTGGAATTGTGAGCGGATAACAATTTCACACAGGAAACAGCTATGACCATGATTACGCCAAGCGCGCAATTAACCCTCACTAAAGGGAACAAAAGCTGGGTACCGGGCCCCCCCTCGAGAGTC

**SHGI7AiL**

8499bp

5’LTR CMV promoter 1-531

R region 532-590

U5 region 591-666

Packaging signal 667-1023

hGH pA 1647-1063 (reverse orientation)

eGFP gene 2384-1665 (reverse orientation)

Intron 7 2483-2408 (reverse orientation)

ADA promoter 2944-2502 (reverse orientation)

ADA LCR 5236-2980 (reverse orientation)

3’LTR DU3 region 5317-5434

R region 5435-5501

U5 region 5502-5578

CGTTACATAACTTACGGTAAATGGCCCGCCTGGCTGACCGCCCAACGACCCCCGCCCATTGACGTCAATAATGACGTATGTTCCCATAGTAACGCCAATAGGGACTTTCCATTGACGTCAATGGGTGGAGTATTTACGGTAAACTGCCCACTTGGCAGTACATCAAGTGTATCATATGCCAAGTACGCCCCCTATTGACGTCAATGACGGTAAATGGCCCGCCTGGCATTATGCCCAGTACATGACCTTATGGGACTTTCCTACTTGGCAGTACATCTACGTATTAGTCATCGCTATTACCATGGTGATGCGGTTTTGGCAGTACATCAATGGGCGTGGATAGCGGTTTGACTCACGGGGATTTCCAAGTCTCCACCCCATTGACGTCAATGGGAGTTTGTTTTGGCACCAAAATCAACGGGACTTTCCAAAATGTCGTAACAACTCCGCCCCATTGACGCAAATGGGCGGTAGGCGTGTACGGTGGGAGGTCTATATAAGCAGAGCTCGTTTAGTGAACCGCGCCAGTCTTCCGATAGACTGCGTCGCCCGGGTACCCGTATTCCCAATAAAGCCTCTTGCTGTTTGCATCCGAATCGTGGTCTCGCTGTTCCTTGGGAGGGTCTCCTCTGAGTGATTGACTACCCACGACGGGGGTCTTTCATTTGGGGGCTCGTCCGGGATTTGGAGACCCCTGCCCAGGGACCACCGACCCACCACCGGGAGGTAAGCTGGCCAGCAACTTATCTGTGTCTGTCCGATTGTCTAGTGTCTATGTTTGATGTTATGCGCCTGCGTCTGTACTAGTTAGCTAACTAGCTCTGTATCTGGCGGACCCGTGGTGGAACTGACGAGTTCTGAACACCCGGCCGCAACCCTGGGAGACGTCCCAGGGACTTTGGGGGCCGTTTTTGTGGCCCGACCTGAGGAAGGGAGTCGATGTGGAATCCGACCCCGTCAGGATATGTGGTTCTGGTAGGAGACGAGAACCTAAAACAGTTCCCGCCTCCGTCTGAATTTTTGCTAAGCTCTAGAGTCGACACGCGTGCGATCGCTCGCGATAGCTTGCCAAGCAAACAACTCAAATGTCCAACTGGCTGGGCATGGCCAGGTAGCCCATGCTGTGTCTGGACGTCCTTCTGCTGGTATAATTATTTTAAAATCACAGGACAGGGAAGGGCCCAGTGGCTCATACCTGTAATCCCAGCAATTTGGGAGGCCGAGGCGGGCGGATTACCTGAGGTCAGGAGATGGAGACCAGACTGGCCAATATGGTGAAACCCCGTCTCTACCAAAAATACAAAAATTAGCTGAGCCTGGTCGTGCATGCCTGGAATCCCAACTATTCGGGAGACTGAGGCAGGAGAATCGCTTGAACCCAGGAGGCGGAGATTGCAGCGAGCCAAGATCGTGCCACTGCACTCCAGCCTGGTTCCCAATAGACCCTGAAGGCCCTACAGGTTGTCTTCCCAACCTGCCCCTTGCTCCATACCACCCGCCTCCACCCCATAATATTATACAAGGACACCTAGTCAAACAAAATGATGCAACTTAATTTTATTAGGACAAGGCTGGTGGGCACTGGAGTGGCAACTTCCAGGGCCAGGAGAGGCACTGGGGAGGGGTCACAGGGATGCCACGTTAACTCTAGAGTCGCGGCCGCTTTACTTGTACAGCTCGTCCATGCCGAGAGTGATCCCGGCGGCGGTCACGAACTCCAGCAGGACCATGTGATCGCGCTTCTCGTTGGGGTCTTTGCTCAGGGCGGACTGGGTGCTCAGGTAGTGGTTGTCGGGCAGCAGCACGGGGCCGTCGCCGATGGGGGTGTTCTGCTGGTAGTGGTCGGCGAGCTGCACGCTGCCGTCCTCGATGTTGTGGCGGATCTTGAAGTTCACCTTGATGCCGTTCTTCTGCTTGTCGGCCATGATATAGACGTTGTGGCTGTTGTAGTTGTACTCCAGCTTGTGCCCCAGGATGTTGCCGTCCTCCTTGAAGTCGATGCCCTTCAGCTCGATGCGGTTCACCAGGGTGTCGCCCTCGAACTTCACCTCGGCGCGGGTCTTGTAGTTGCCGTCGTCCTTGAAGAAGATGGTGCGCTCCTGGACGTAGCCTTCGGGCATGGCGGACTTGAAGAAGTCGTGCTGCTTCATGTGGTCGGGGTAGCGGCTGAAGCACTGCACGCCGTAGGTCAGGGTGGTCACGAGGGTGGGCCAGGGCACGGGCAGCTTGCCGGTGGTGCAGATGAACTTCAGGGTCAGCTTGCCGTAGGTGGCATCGCCCTCGCCCTCGCCGGACACGCTGAACTTGTGGCCGTTTACGTCGCCGTCCAGCTCGACCAGGATGGGCACCACCCCGGTGAACAGCTCCTCGCCCTTGCTCACCATGGTGGCGACCGGATCTCCACAGCCTGTAGAGAAGCAGAATAGAGCCAAGTATGGGAGGAGGCAGTGAGGAGGGACCCCATGGCCAGCCCAGGCCCTCACCTCTTTTACTGATCCGGTGGTGCCCTCGTGCGCCCCGGCGCTGCTCCCTCCGCCGCCGCTCGGTGGGTCTCTGCCGGCTCGGTGGCCGCTCGGCTTTCCCTGGGGCCAGCGGTGGCCGCGGCCGGCCACGCTCTTCTTAACGGGCCGGGCCCCGCCTCCCGCCCCGCCCGCCGCCGTTCCCGGGGCCCGCCCTCTCGCCGGAGCCCCCGCCCCGCCCGCCGGCCTGGATCGCGCATTTCCTGGAATTCTGGACCCGGCGTCCTCGCCCGCCGACCCGAAAACTACGGCCCCGCGGCGCCCGACCGGACCCACCTGTGGGCGCGCGCTCCAGCCGCCTCGCTGGGCGCTGCCTTCCCCCCGGCTCCTCCAGCAACTTTCAAGCGTCCCCCTGCAGTCTGGGCGCTGCTGTGCGCCAGGCCCAAGCTAGAAGCGGGGACCCCGGGAGCATCGCAAGCCTCGAGGGCGCGCCTTAATTAACACCGGCGAAGCTTGGATCCATGCCACATAGCAAGGTGCTGGGTCACTGATGCTAACATCTAAACAGGGGCCAGGTGCGGTGGCTCACTCTTGAAATCCCAGCACTTTGGGAGGCTGAGGTGGGTGGATCACTTGAGGCCAGGAGTTTGAGACCTCCTCTTCACAGAGGTGGAAGGGAGGACCAGCGGGGGCAAGTGTATCCCAAGTCCCAGCTACTTGGGAGGCTGAGGCAGGAGGATCGCATGAACCCAGGAGGCAGAGGTTGCAGTGAGCCAAAATCGTGCCACTGCACTCCAGCTTGGGTGACAGAGGAAGACTCCAGCTCAAAAAAAAAAAAAAAAAAAAAGTCACTGAAGGCACCTGGTACCCAGGACTACTTCAAGGACAGCCATTGTTATGGCCAACATGGTGAAACCCTGTCTCTACTAAAAACACAAAAATTAGCCAGGCATGGTGGCGCACACCTGTAATCCCAACTACTTGGGAGACTGAGGCAGAAGAATCACTTGAACCCGGGAGGCAGAGGTTGTAGTGAGCCGAGATTGCACCACTGCACTCCAGCCTGGGCGACAGAGTGAGACTCTGTCTCAAAAGTCAAAGTAGGACCATTACCACCATGATGATGATGCTGACCAATGGCAGCTATTGCTGCAGCACCTGCTCTGAGCCAGGCCTGCACTTGGTGTTTGAGAGGTGGGTATGGGTATAATTCTCATTTTAGAGAGGGGAAACTGAGGCACCGTGAAGTTAAATGACTCATTCAAGCTCCTCTAGCTGGAAAGTGGGAAATCAGGACTCAGACCCAGAGAGCCTGACCCCAGCTGGTGTCCAAGTTCAAATTCCTTGCTGTTCTAGCTCCCTGGTCACTAGGAGTGCAGAGGTTATCCCCCACAACTGCTCAGGGGCCTGAGAACATTAAGGGCTGAGGGCAAGCCTACCCCCAACACCTTGGGTAAATACAGATTCCAACCTCACTCTGCGCCCACAGCCTGCTCCTCTCCACCTCCCCAGCCAGACCATGGTCCTACTACCTTGCCCCGCCCAGGGGGTACCACAGGGACCCCTTCAAGACACCTGAGCAGGTGGACAAGTCTCTCCTGGATCACAGGGCCCCCAGCCACCCAGGTCAAGCCCACCCTGAGGTTGAATGCAGGATTGGGAAGCTGCCTGGCCTCCCCTGCCTCCCTGTGTGAACACAGACCACTTCCTCTCTCTGATCCTCCCTTCCTCCTCCGAAGCAAAAGGCTTCGATGAAACTCAGTCTCCTTTGTTCCCCTCCACCACCAACTGCCATGTGAGTCAAAATGGGTAAGGCAGGCTCTTCCTAGGCACATTTTTGTTTTAACAGGTGGCTCTGGTAACTGAAAGCCACTGCAAGCCCAAAGAAGTGCAGCTGGTGGGTTTTGTGTCAGGAAGACAAAAAGGAGGAGGGAGGAGGGAGATTCTGCAGCTGGAGAGGCCTCCCAGAGTCTACTGCAACCCCCACTCCAGTCCCCAGGAAGGAAACGCACCCCTGAGGGCCTGCTGTTACCAGCCAGGCACTGTACAAAAACCAATCAGCATGTCAGTTTCCCCAAGAACCCCAAGATCTTGGTTTTGTCACCCCATTTTACAGAGGGGGAAGCTGAGTTTCAGGAGTTGTCTACCTGAGGTACCCTAGCTGCTAAGCGGCAGGCAGGAATTCAACCCCAGTACATCTGAAGCCCAGGGCTAATGCACTGGCCCACCCTGCCACCCTGATTCTAACTCTTCCCCCCACACTGCCCCCTCTTTTTTGCATAAAGTGGAATTGGCAAATGAGCAAAGCAATGAACAGCTTCTACTTGAGTGCAGGCAGATCTGGGTTCAAGTCTAGCTCTGCTGCTTGCCAGCTGTGTGACTTTGGCAAGTTCCCTCCCCTCTCTGAGTCTCAGTTTCCTCATAATAAGTGAAGTTAATAATAGGACAGGACCTTCCTCAGAGGGCTGACATAAACATTAAATGAGTCACTGTATGCAAAGCAGGACAGCGCCTCGTGCAGAGTAGACATTCAAATATGAGACAGAAAAGGCACATTGCTGATTTAGCCCCAATTCCCCTCTGCATCAGAGAGGGACAGTTTAGAAACTGCTGTCCCTTGGCCGGGTGCGGTGGCTCATGCCTGTAATCCCGGCACTTTGGGAGGCTGAGGTGGGCGGATCACCTGAAGTCGGGAGTTCGAGACCAGCCTGGCCAACAAGGAGAAACCCCGTCTCTACTAAAAATACAAAAAATTAGCCGGGCATGGTGGTGCATGCCTTAAGTATCGATACACGTGGTTTAAACGTCGACCATAGATAAAATAAAAGATTTTATTTAGTCTCCAGAAAAAGGGGGGAATGAAAGACCCCACCTGTAGGTTTGGCAAGCTAGCGATATCAGTTCGCTTCTCGCTTCTGTTCGCGCGCTTCTGCTCCCCGAGCTCAATAAAAGAGCCCACAACCCCTCACTCGGGGCGCCAGTCCTCCGATTGACTGAGTCGCCCGGGTACCCGTGTATCCAATAAACCCTCTTGCAGTTGCATCCGACTTGTGGTCTCGCTGTTCCTTGGGAGGGTCTCCTCTGAGTGATTGACTACCCGTCAGCGGGGGTCTTTCATTTGGGGGCTCGTCCGGGATCGCGGCCGCCACCGCGGTGGAGCTCCAATTCGCCCTATAGTGAGTCGTATTACGCGCGCTCACTGGCCGTCGTTTTACAACGTCGTGACTGGGAAAACCCTGGCGTTACCCAACTTAATCGCCTTGCAGCACATCCCCCTTTCGCCAGCTGGCGTAATAGCGAAGAGGCCCGCACCGATCGCCCTTCCCAACAGTTGCGCAGCCTGAATGGCGAATGGGACGCGCCCTGTAGCGGCGCATTAAGCGCGGCGGGTGTGGTGGTTACGCGCAGCGTGACCGCTACACTTGCCAGCGCCCTAGCGCCCGCTCCTTTCGCTTTCTTCCCTTCCTTTCTCGCCACGTTCGCCGGCTTTCCCCGTCAAGCTCTAAATCGGGGGCTCCCTTTAGGGTTCCGATTTAGTGCTTTACGGCACCTCGACCCCAAAAAACTTGATTAGGGTGATGGTTCACGTAGTGGGCCATCGCCCTGATAGACGGTTTTTCGCCCTTTGACGTTGGAGTCCACGTTCTTTAATAGTGGACTCTTGTTCCAAACTGGAACAACACTCAACCCTATCTCGGTCTATTCTTTTGATTTATAAGGGATTTTGCCGATTTCGGCCTATTGGTTAAAAAATGAGCTGATTTAACAAAAATTTAACGCGAATTTTAACAAAATATTAACGCTTACAATTTAGGTGGCACTTTTCGGGGAAATGTGCGCGGAACCCCTATTTGTTTATTTTTCTAAATACATTCAAATATGTATCCGCTCATGAGACAATAACCCTGATAAATGCTTCAATAATATTGAAAAAGGAAGAGTATGAGTATTCAACATTTCCGTGTCGCCCTTATTCCCTTTTTTGCGGCATTTTGCCTTCCTGTTTTTGCTCACCCAGAAACGCTGGTGAAAGTAAAAGATGCTGAAGATCAGTTGGGTGCACGAGTGGGTTACATCGAACTGGATCTCAACAGCGGTAAGATCCTTGAGAGTTTTCGCCCCGAAGAACGTTTTCCAATGATGAGCACTTTTAAAGTTCTGCTATGTGGCGCGGTATTATCCCGTATTGACGCCGGGCAAGAGCAACTCGGTCGCCGCATACACTATTCTCAGAATGACTTGGTTGAGTACTCACCAGTCACAGAAAAGCATCTTACGGATGGCATGACAGTAAGAGAATTATGCAGTGCTGCCATAACCATGAGTGATAACACTGCGGCCAACTTACTTCTGACAACGATCGGAGGACCGAAGGAGCTAACCGCTTTTTTGCACAACATGGGGGATCATGTAACTCGCCTTGATCGTTGGGAACCGGAGCTGAATGAAGCCATACCAAACGACGAGCGTGACACCACGATGCCTGTAGCAATGGCAACAACGTTGCGCAAACTATTAACTGGCGAACTACTTACTCTAGCTTCCCGGCAACAATTAATAGACTGGATGGAGGCGGATAAAGTTGCAGGACCACTTCTGCGCTCGGCCCTTCCGGCTGGCTGGTTTATTGCTGATAAATCTGGAGCCGGTGAGCGTGGGTCTCGCGGTATCATTGCAGCACTGGGGCCAGATGGTAAGCCCTCCCGTATCGTAGTTATCTACACGACGGGGAGTCAGGCAACTATGGATGAACGAAATAGACAGATCGCTGAGATAGGTGCCTCACTGATTAAGCATTGGTAACTGTCAGACCAAGTTTACTCATATATACTTTAGATTGATTTAAAACTTCATTTTTAATTTAAAAGGATCTAGGTGAAGATCCTTTTTGATAATCTCATGACCAAAATCCCTTAACGTGAGTTTTCGTTCCACTGAGCGTCAGACCCCGTAGAAAAGATCAAAGGATCTTCTTGAGATCCTTTTTTTCTGCGCGTAATCTGCTGCTTGCAAACAAAAAAACCACCGCTACCAGCGGTGGTTTGTTTGCCGGATCAAGAGCTACCAACTCTTTTTCCGAAGGTAACTGGCTTCAGCAGAGCGCAGATACCAAATACTGTCCTTCTAGTGTAGCCGTAGTTAGGCCACCACTTCAAGAACTCTGTAGCACCGCCTACATACCTCGCTCTGCTAATCCTGTTACCAGTGGCTGCTGCCAGTGGCGATAAGTCGTGTCTTACCGGGTTGGACTCAAGACGATAGTTACCGGATAAGGCGCAGCGGTCGGGCTGAACGGGGGGTTCGTGCACACAGCCCAGCTTGGAGCGAACGACCTACACCGAACTGAGATACCTACAGCGTGAGCTATGAGAAAGCGCCACGCTTCCCGAAGGGAGAAAGGCGGACAGGTATCCGGTAAGCGGCAGGGTCGGAACAGGAGAGCGCACGAGGGAGCTTCCAGGGGGAAACGCCTGGTATCTTTATAGTCCTGTCGGGTTTCGCCACCTCTGACTTGAGCGTCGATTTTTGTGATGCTCGTCAGGGGGGCGGAGCCTATGGAAAAACGCCAGCAACGCGGCCTTTTTACGGTTCCTGGCCTTTTGCTGGCCTTTTGCTCACATGTTCTTTCCTGCGTTATCCCCTGATTCTGTGGATAACCGTATTACCGCCTTTGAGTGAGCTGATACCGCTCGCCGCAGCCGAACGACCGAGCGCAGCGAGTCAGTGAGCGAGGAAGCGGAAGAGCGCCCAATACGCAAACCGCCTCTCCCCGCGCGTTGGCCGATTCATTAATGCAGCTGGCACGACAGGTTTCCCGACTGGAAAGCGGGCAGTGAGCGCAACGCAATTAATGTGAGTTAGCTCACTCATTAGGCACCCCAGGCTTTACACTTTATGCTTCCGGCTCGTATGTTGTGTGGAATTGTGAGCGGATAACAATTTCACACAGGAAACAGCTATGACCATGATTACGCCAAGCGCGCAATTAACCCTCACTAAAGGGAACAAAAGCTGGGTACCGGGCCCCCCCTCGAGAGTC

**SLHGA**

8403 bp

5’LTR CMV promoter 1-531

R region 532-590

U5 region 596-666

Packaging signal 667-1023

ADA LCR 1069-3325

hGH pA 3945-3361 (reverse orientation)

eGFP gene 4682-3963 (reverse orientation)

ADA promoter 5138-4696 (reverse orientation)

3’LTR DU3 region 5221-5338

R region 5339-5405

U5 region 5406-5482

CGTTACATAACTTACGGTAAATGGCCCGCCTGGCTGACCGCCCAACGACCCCCGCCCATTGACGTCAATAATGACGTATGTTCCCATAGTAACGCCAATAGGGACTTTCCATTGACGTCAATGGGTGGAGTATTTACGGTAAACTGCCCACTTGGCAGTACATCAAGTGTATCATATGCCAAGTACGCCCCCTATTGACGTCAATGACGGTAAATGGCCCGCCTGGCATTATGCCCAGTACATGACCTTATGGGACTTTCCTACTTGGCAGTACATCTACGTATTAGTCATCGCTATTACCATGGTGATGCGGTTTTGGCAGTACATCAATGGGCGTGGATAGCGGTTTGACTCACGGGGATTTCCAAGTCTCCACCCCATTGACGTCAATGGGAGTTTGTTTTGGCACCAAAATCAACGGGACTTTCCAAAATGTCGTAACAACTCCGCCCCATTGACGCAAATGGGCGGTAGGCGTGTACGGTGGGAGGTCTATATAAGCAGAGCTCGTTTAGTGAACCGCGCCAGTCTTCCGATAGACTGCGTCGCCCGGGTACCCGTATTCCCAATAAAGCCTCTTGCTGTTTGCATCCGAATCGTGGTCTCGCTGTTCCTTGGGAGGGTCTCCTCTGAGTGATTGACTACCCACGACGGGGGTCTTTCATTTGGGGGCTCGTCCGGGATTTGGAGACCCCTGCCCAGGGACCACCGACCCACCACCGGGAGGTAAGCTGGCCAGCAACTTATCTGTGTCTGTCCGATTGTCTAGTGTCTATGTTTGATGTTATGCGCCTGCGTCTGTACTAGTTAGCTAACTAGCTCTGTATCTGGCGGACCCGTGGTGGAACTGACGAGTTCTGAACACCCGGCCGCAACCCTGGGAGACGTCCCAGGGACTTTGGGGGCCGTTTTTGTGGCCCGACCTGAGGAAGGGAGTCGATGTGGAATCCGACCCCGTCAGGATATGTGGTTCTGGTAGGAGACGAGAACCTAAAACAGTTCCCGCCTCCGTCTGAATTTTTGCTAAGCTCTAGAGTCGACACGCGCCCACGTGTATCGATACTTAAGGCATGCACCACCATGCCCGGCTAATTTTTTGTATTTTTAGTAGAGACGGGGTTTCTCCTTGTTGGCCAGGCTGGTCTCGAACTCCCGACTTCAGGTGATCCGCCCACCTCAGCCTCCCAAAGTGCCGGGATTACAGGCATGAGCCACCGCACCCGGCCAAGGGACAGCAGTTTCTAAACTGTCCCTCTCTGATGCAGAGGGGAATTGGGGCTAAATCAGCAATGTGCCTTTTCTGTCTCATATTTGAATGTCTACTCTGCACGAGGCGCTGTCCTGCTTTGCATACAGTGACTCATTTAATGTTTATGTCAGCCCTCTGAGGAAGGTCCTGTCCTATTATTAACTTCACTTATTATGAGGAAACTGAGACTCAGAGAGGGGAGGGAACTTGCCAAAGTCACACAGCTGGCAAGCAGCAGAGCTAGACTTGAACCCAGATCTGCCTGCACTCAAGTAGAAGCTGTTCATTGCTTTGCTCATTTGCCAATTCCACTTTATGCAAAAAAGAGGGGGCAGTGTGGGGGGAAGAGTTAGAATCAGGGTGGCAGGGTGGGCCAGTGCATTAGCCCTGGGCTTCAGATGTACTGGGGTTGAATTCCTGCCTGCCGCTTAGCAGCTAGGGTACCTCAGGTAGACAACTCCTGAAACTCAGCTTCCCCCTCTGTAAAATGGGGTGACAAAACCAAGATCTTGGGGTTCTTGGGGAAACTGACATGCTGATTGGTTTTTGTACAGTGCCTGGCTGGTAACAGCAGGCCCTCAGGGGTGCGTTTCCTTCCTGGGGACTGGAGTGGGGGTTGCAGTAGACTCTGGGAGGCCTCTCCAGCTGCAGAATCTCCCTCCTCCCTCCTCCTTTTTGTCTTCCTGACACAAAACCCACCAGCTGCACTTCTTTGGGCTTGCAGTGGCTTTCAGTTACCAGAGCCACCTGTTAAAACAAAAATGTGCCTAGGAAGAGCCTGCCTTACCCATTTTGACTCACATGGCAGTTGGTGGTGGAGGGGAACAAAGGAGACTGAGTTTCATCGAAGCCTTTTGCTTCGGAGGAGGAAGGGAGGATCAGAGAGAGGAAGTGGTCTGTGTTCACACAGGGAGGCAGGGGAGGCCAGGCAGCTTCCCAATCCTGCATTCAACCTCAGGGTGGGCTTGACCTGGGTGGCTGGGGGCCCTGTGATCCAGGAGAGACTTGTCCACCTGCTCAGGTGTCTTGAAGGGGTCCCTGTGGTACCCCCTGGGCGGGGCAAGGTAGTAGGACCATGGTCTGGCTGGGGAGGTGGAGAGGAGCAGGCTGTGGGCGCAGAGTGAGGTTGGAATCTGTATTTACCCAAGGTGTTGGGGGTAGGCTTGCCCTCAGCCCTTAATGTTCTCAGGCCCCTGAGCAGTTGTGGGGGATAACCTCTGCACTCCTAGTGACCAGGGAGCTAGAACAGCAAGGAATTTGAACTTGGACACCAGCTGGGGTCAGGCTCTCTGGGTCTGAGTCCTGATTTCCCACTTTCCAGCTAGAGGAGCTTGAATGAGTCATTTAACTTCACGGTGCCTCAGTTTCCCCTCTCTAAAATGAGAATTATACCCATACCCACCTCTCAAACACCAAGTGCAGGCCTGGCTCAGAGCAGGTGCTGCAGCAATAGCTGCCATTGGTCAGCATCATCATCATGGTGGTAATGGTCCTACTTTGACTTTTGAGACAGAGTCTCACTCTGTCGCCCAGGCTGGAGTGCAGTGGTGCAATCTCGGCTCACTACAACCTCTGCCTCCCGGGTTCAAGTGATTCTTCTGCCTCAGTCTCCCAAGTAGTTGGGATTACAGGTGTGCGCCACCATGCCTGGCTAATTTTTGTGTTTTTAGTAGAGACAGGGTTTCACCATGTTGGCCATAACAATGGCTGTCCTTGAAGTAGTCCTGGGTACCAGGTGCCTTCAGTGACTTTTTTTTTTTTTTTTTTTTTGAGCTGGAGTCTTCCTCTGTCACCCAAGCTGGAGTGCAGTGGCACGATTTTGGCTCACTGCAACCTCTGCCTCCTGGGTTCATGCGATCCTCCTGCCTCAGCCTCCCAAGTAGCTGGGACTTGGGATACACTTGCCCCCGCTGGTCCTCCCTTCCACCTCTGTGAAGAGGAGGTCTCAAACTCCTGGCCTCAAGTGATCCACCCACCTCAGCCTCCCAAAGTGCTGGGATTTCAAGAGTGAGCCACCGCACCTGGCCCCTGTTTAGATGTTAGCATCAGTGACCCAGCACCTTGCTATGTGGCATGGATCCAAGCTTCGCCGGTGTTAATTAAGTTTCGATAGCTTGCCAAGCAAACAACTCAAATGTCCAACTGGCTGGGCATGGCCAGGTAGCCCATGCTGTGTCTGGACGTCCTTCTGCTGGTATAATTATTTTAAAATCACAGGACAGGGAAGGGCCCAGTGGCTCATACCTGTAATCCCAGCAATTTGGGAGGCCGAGGCGGGCGGATTACCTGAGGTCAGGAGATGGAGACCAGACTGGCCAATATGGTGAAACCCCGTCTCTACCAAAAATACAAAAATTAGCTGAGCCTGGTCGTGCATGCCTGGAATCCCAACTATTCGGGAGACTGAGGCAGGAGAATCGCTTGAACCCAGGAGGCGGAGATTGCAGCGAGCCAAGATCGTGCCACTGCACTCCAGCTTGGTTCCCAATAGACCCCGCAGGCCCTACAGGTTGCCTTCCCAACTTGCCCCTTGCTCCATACCACCCCCCTCCACCCCATAATATTATAGAAGGACACCTAGTCAGACAAAATGATGCAACTTAATTTTATTAGGACAAGGCTGGTGGGCACTGGAGTGGCAACTTCCAGGGCCAGGAGAGGCACTGGGGAGGGGTCACAGGGATGCCACGTTAACTCTAGAGTCGCGGCCGCTTTACTTGTACAGCTCGTCCATGCCGAGAGTGATCCCGGCGGCGGTCACGAACTCCAGCAGGACCATGTGATCGCGCTTCTCGTTGGGGTCTTTGCTCAGGGCGGACTGGGTGCTCAGGTAGTGGTTGTCGGGCAGCAGCACGGGGCCGTCGCCGATGGGGGTGTTCTGCTGGTAGTGGTCGGCGAGCTGCACGCTGCCGTCCTCGATGTTGTGGCGGATCTTGAAGTTCACCTTGATGCCGTTCTTCTGCTTGTCGGCCATGATATAGACGTTGTGGCTGTTGTAGTTGTACTCCAGCTTGTGCCCCAGGATGTTGCCGTCCTCCTTGAAGTCGATGCCCTTCAGCTCGATGCGGTTCACCAGGGTGTCGCCCTCGAACTTCACCTCGGCGCGGGTCTTGTAGTTGCCGTCGTCCTTGAAGAAGATGGTGCGCTCCTGGACGTAGCCTTCGGGCATGGCGGACTTGAAGAAGTCGTGCTGCTTCATGTGGTCGGGGTAGCGGCTGAAGCACTGCACGCCGTAGGTCAGGGTGGTCACGAGGGTGGGCCAGGGCACGGGCAGCTTGCCGGTGGTGCAGATGAACTTCAGGGTCAGCTTGCCGTAGGTGGCATCGCCCTCGCCCTCGCCGGACACGCTGAACTTGTGGCCGTTTACGTCGCCGTCCAGCTCGACCAGGATGGGCACCACCCCGGTGAACAGCTCCTCGCCCTTGCTCACCATGGTGGCGACCGGTGGTGCCCTCGTGCGCCCCGGCGCTGCTCCCTCCGCCGCCGCTCGGTGGGTCTCTGCCGGCTCGGTGGCCGCTCGGCTTTCCCTGGGGCCAGCGGTGGCCGCGGCCGGCCACGCTCTTCTTAACGGGCCGGGCCCCGCCTCCCGCCCCGCCCGCCGCCGTTCCCGGGGCCCGCCCTCTCGCCGGAGCCCCCGCCCCGCCCGCCGGCCTGGATCGCGCATTTCCTGGAATTCTGGACCCGGCGTCCTCGCCCGCCGACCCGAAAACTACGGCCCCGCGGCGCCCGACCGGACCCACCTGTGGGCGCGCGCTCCAGCCGCCTCGCTGGGCGCTGCCTTCCCCCCGGCTCCTCCAGCAACTTTCAAGCGTCCCCCTGCAGTCTGGGCGCTGCTGTGCGCCAGGCCCAAGCTAGAAGCGGGGACCCCGGGAGCATCGCAAGCCTCGAGGGCGCGCCTTAATTAACACGTGGTTTAAACGTCGACCATAGATAAAATAAAAGATTTTATTTAGTCTCCAGAAAAAGGGGGGAATGAAAGACCCCACCTGTAGGTTTGGCAAGCTAGCGATATCAGTTCGCTTCTCGCTTCTGTTCGCGCGCTTCTGCTCCCCGAGCTCAATAAAAGAGCCCACAACCCCTCACTCGGGGCGCCAGTCCTCCGATTGACTGAGTCGCCCGGGTACCCGTGTATCCAATAAACCCTCTTGCAGTTGCATCCGACTTGTGGTCTCGCTGTTCCTTGGGAGGGTCTCCTCTGAGTGATTGACTACCCGTCAGCGGGGGTCTTTCATTTGGGGGCTCGTCCGGGATCGCGGCCGCCACCGCGGTGGAGCTCCAATTCGCCCTATAGTGAGTCGTATTACGCGCGCTCACTGGCCGTCGTTTTACAACGTCGTGACTGGGAAAACCCTGGCGTTACCCAACTTAATCGCCTTGCAGCACATCCCCCTTTCGCCAGCTGGCGTAATAGCGAAGAGGCCCGCACCGATCGCCCTTCCCAACAGTTGCGCAGCCTGAATGGCGAATGGGACGCGCCCTGTAGCGGCGCATTAAGCGCGGCGGGTGTGGTGGTTACGCGCAGCGTGACCGCTACACTTGCCAGCGCCCTAGCGCCCGCTCCTTTCGCTTTCTTCCCTTCCTTTCTCGCCACGTTCGCCGGCTTTCCCCGTCAAGCTCTAAATCGGGGGCTCCCTTTAGGGTTCCGATTTAGTGCTTTACGGCACCTCGACCCCAAAAAACTTGATTAGGGTGATGGTTCACGTAGTGGGCCATCGCCCTGATAGACGGTTTTTCGCCCTTTGACGTTGGAGTCCACGTTCTTTAATAGTGGACTCTTGTTCCAAACTGGAACAACACTCAACCCTATCTCGGTCTATTCTTTTGATTTATAAGGGATTTTGCCGATTTCGGCCTATTGGTTAAAAAATGAGCTGATTTAACAAAAATTTAACGCGAATTTTAACAAAATATTAACGCTTACAATTTAGGTGGCACTTTTCGGGGAAATGTGCGCGGAACCCCTATTTGTTTATTTTTCTAAATACATTCAAATATGTATCCGCTCATGAGACAATAACCCTGATAAATGCTTCAATAATATTGAAAAAGGAAGAGTATGAGTATTCAACATTTCCGTGTCGCCCTTATTCCCTTTTTTGCGGCATTTTGCCTTCCTGTTTTTGCTCACCCAGAAACGCTGGTGAAAGTAAAAGATGCTGAAGATCAGTTGGGTGCACGAGTGGGTTACATCGAACTGGATCTCAACAGCGGTAAGATCCTTGAGAGTTTTCGCCCCGAAGAACGTTTTCCAATGATGAGCACTTTTAAAGTTCTGCTATGTGGCGCGGTATTATCCCGTATTGACGCCGGGCAAGAGCAACTCGGTCGCCGCATACACTATTCTCAGAATGACTTGGTTGAGTACTCACCAGTCACAGAAAAGCATCTTACGGATGGCATGACAGTAAGAGAATTATGCAGTGCTGCCATAACCATGAGTGATAACACTGCGGCCAACTTACTTCTGACAACGATCGGAGGACCGAAGGAGCTAACCGCTTTTTTGCACAACATGGGGGATCATGTAACTCGCCTTGATCGTTGGGAACCGGAGCTGAATGAAGCCATACCAAACGACGAGCGTGACACCACGATGCCTGTAGCAATGGCAACAACGTTGCGCAAACTATTAACTGGCGAACTACTTACTCTAGCTTCCCGGCAACAATTAATAGACTGGATGGAGGCGGATAAAGTTGCAGGACCACTTCTGCGCTCGGCCCTTCCGGCTGGCTGGTTTATTGCTGATAAATCTGGAGCCGGTGAGCGTGGGTCTCGCGGTATCATTGCAGCACTGGGGCCAGATGGTAAGCCCTCCCGTATCGTAGTTATCTACACGACGGGGAGTCAGGCAACTATGGATGAACGAAATAGACAGATCGCTGAGATAGGTGCCTCACTGATTAAGCATTGGTAACTGTCAGACCAAGTTTACTCATATATACTTTAGATTGATTTAAAACTTCATTTTTAATTTAAAAGGATCTAGGTGAAGATCCTTTTTGATAATCTCATGACCAAAATCCCTTAACGTGAGTTTTCGTTCCACTGAGCGTCAGACCCCGTAGAAAAGATCAAAGGATCTTCTTGAGATCCTTTTTTTCTGCGCGTAATCTGCTGCTTGCAAACAAAAAAACCACCGCTACCAGCGGTGGTTTGTTTGCCGGATCAAGAGCTACCAACTCTTTTTCCGAAGGTAACTGGCTTCAGCAGAGCGCAGATACCAAATACTGTCCTTCTAGTGTAGCCGTAGTTAGGCCACCACTTCAAGAACTCTGTAGCACCGCCTACATACCTCGCTCTGCTAATCCTGTTACCAGTGGCTGCTGCCAGTGGCGATAAGTCGTGTCTTACCGGGTTGGACTCAAGACGATAGTTACCGGATAAGGCGCAGCGGTCGGGCTGAACGGGGGGTTCGTGCACACAGCCCAGCTTGGAGCGAACGACCTACACCGAACTGAGATACCTACAGCGTGAGCTATGAGAAAGCGCCACGCTTCCCGAAGGGAGAAAGGCGGACAGGTATCCGGTAAGCGGCAGGGTCGGAACAGGAGAGCGCACGAGGGAGCTTCCAGGGGGAAACGCCTGGTATCTTTATAGTCCTGTCGGGTTTCGCCACCTCTGACTTGAGCGTCGATTTTTGTGATGCTCGTCAGGGGGGCGGAGCCTATGGAAAAACGCCAGCAACGCGGCCTTTTTACGGTTCCTGGCCTTTTGCTGGCCTTTTGCTCACATGTTCTTTCCTGCGTTATCCCCTGATTCTGTGGATAACCGTATTACCGCCTTTGAGTGAGCTGATACCGCTCGCCGCAGCCGAACGACCGAGCGCAGCGAGTCAGTGAGCGAGGAAGCGGAAGAGCGCCCAATACGCAAACCGCCTCTCCCCGCGCGTTGGCCGATTCATTAATGCAGCTGGCACGACAGGTTTCCCGACTGGAAAGCGGGCAGTGAGCGCAACGCAATTAATGTGAGTTAGCTCACTCATTAGGCACCCCAGGCTTTACACTTTATGCTTCCGGCTCGTATGTTGTGTGGAATTGTGAGCGGATAACAATTTCACACAGGAAACAGCTATGACCATGATTACGCCAAGCGCGCAATTAACCCTCACTAAAGGGAACAAAAGCTGGGTACCGGGCCCCCCCTCGAGAGTC

**SLHGI7A**

8507 bp

5’LTR CMV promoter 1-531

R region 532-590

U5 region 591-666

Packaging signal 667-1023

ADA LCR 1069-3325

hGH pA 3945-3361 (reverse orientation)

eGFP gene 4682-3963 (reverse orientation)

Intron 7 4781-4706 (reverse orientation)

ADA promoter 5242-4800 (reverse orientation)

3’LTR DU3 region 5325-5442

R region 5443-5509

U5 region 5510-5586

CGTTACATAACTTACGGTAAATGGCCCGCCTGGCTGACCGCCCAACGACCCCCGCCCATTGACGTCAATAATGACGTATGTTCCCATAGTAACGCCAATAGGGACTTTCCATTGACGTCAATGGGTGGAGTATTTACGGTAAACTGCCCACTTGGCAGTACATCAAGTGTATCATATGCCAAGTACGCCCCCTATTGACGTCAATGACGGTAAATGGCCCGCCTGGCATTATGCCCAGTACATGACCTTATGGGACTTTCCTACTTGGCAGTACATCTACGTATTAGTCATCGCTATTACCATGGTGATGCGGTTTTGGCAGTACATCAATGGGCGTGGATAGCGGTTTGACTCACGGGGATTTCCAAGTCTCCACCCCATTGACGTCAATGGGAGTTTGTTTTGGCACCAAAATCAACGGGACTTTCCAAAATGTCGTAACAACTCCGCCCCATTGACGCAAATGGGCGGTAGGCGTGTACGGTGGGAGGTCTATATAAGCAGAGCTCGTTTAGTGAACCGCGCCAGTCTTCCGATAGACTGCGTCGCCCGGGTACCCGTATTCCCAATAAAGCCTCTTGCTGTTTGCATCCGAATCGTGGTCTCGCTGTTCCTTGGGAGGGTCTCCTCTGAGTGATTGACTACCCACGACGGGGGTCTTTCATTTGGGGGCTCGTCCGGGATTTGGAGACCCCTGCCCAGGGACCACCGACCCACCACCGGGAGGTAAGCTGGCCAGCAACTTATCTGTGTCTGTCCGATTGTCTAGTGTCTATGTTTGATGTTATGCGCCTGCGTCTGTACTAGTTAGCTAACTAGCTCTGTATCTGGCGGACCCGTGGTGGAACTGACGAGTTCTGAACACCCGGCCGCAACCCTGGGAGACGTCCCAGGGACTTTGGGGGCCGTTTTTGTGGCCCGACCTGAGGAAGGGAGTCGATGTGGAATCCGACCCCGTCAGGATATGTGGTTCTGGTAGGAGACGAGAACCTAAAACAGTTCCCGCCTCCGTCTGAATTTTTGCTAAGCTCTAGAGTCGACACGCGCCCACGTGTATCGATACTTAAGGCATGCACCACCATGCCCGGCTAATTTTTTGTATTTTTAGTAGAGACGGGGTTTCTCCTTGTTGGCCAGGCTGGTCTCGAACTCCCGACTTCAGGTGATCCGCCCACCTCAGCCTCCCAAAGTGCCGGGATTACAGGCATGAGCCACCGCACCCGGCCAAGGGACAGCAGTTTCTAAACTGTCCCTCTCTGATGCAGAGGGGAATTGGGGCTAAATCAGCAATGTGCCTTTTCTGTCTCATATTTGAATGTCTACTCTGCACGAGGCGCTGTCCTGCTTTGCATACAGTGACTCATTTAATGTTTATGTCAGCCCTCTGAGGAAGGTCCTGTCCTATTATTAACTTCACTTATTATGAGGAAACTGAGACTCAGAGAGGGGAGGGAACTTGCCAAAGTCACACAGCTGGCAAGCAGCAGAGCTAGACTTGAACCCAGATCTGCCTGCACTCAAGTAGAAGCTGTTCATTGCTTTGCTCATTTGCCAATTCCACTTTATGCAAAAAAGAGGGGGCAGTGTGGGGGGAAGAGTTAGAATCAGGGTGGCAGGGTGGGCCAGTGCATTAGCCCTGGGCTTCAGATGTACTGGGGTTGAATTCCTGCCTGCCGCTTAGCAGCTAGGGTACCTCAGGTAGACAACTCCTGAAACTCAGCTTCCCCCTCTGTAAAATGGGGTGACAAAACCAAGATCTTGGGGTTCTTGGGGAAACTGACATGCTGATTGGTTTTTGTACAGTGCCTGGCTGGTAACAGCAGGCCCTCAGGGGTGCGTTTCCTTCCTGGGGACTGGAGTGGGGGTTGCAGTAGACTCTGGGAGGCCTCTCCAGCTGCAGAATCTCCCTCCTCCCTCCTCCTTTTTGTCTTCCTGACACAAAACCCACCAGCTGCACTTCTTTGGGCTTGCAGTGGCTTTCAGTTACCAGAGCCACCTGTTAAAACAAAAATGTGCCTAGGAAGAGCCTGCCTTACCCATTTTGACTCACATGGCAGTTGGTGGTGGAGGGGAACAAAGGAGACTGAGTTTCATCGAAGCCTTTTGCTTCGGAGGAGGAAGGGAGGATCAGAGAGAGGAAGTGGTCTGTGTTCACACAGGGAGGCAGGGGAGGCCAGGCAGCTTCCCAATCCTGCATTCAACCTCAGGGTGGGCTTGACCTGGGTGGCTGGGGGCCCTGTGATCCAGGAGAGACTTGTCCACCTGCTCAGGTGTCTTGAAGGGGTCCCTGTGGTACCCCCTGGGCGGGGCAAGGTAGTAGGACCATGGTCTGGCTGGGGAGGTGGAGAGGAGCAGGCTGTGGGCGCAGAGTGAGGTTGGAATCTGTATTTACCCAAGGTGTTGGGGGTAGGCTTGCCCTCAGCCCTTAATGTTCTCAGGCCCCTGAGCAGTTGTGGGGGATAACCTCTGCACTCCTAGTGACCAGGGAGCTAGAACAGCAAGGAATTTGAACTTGGACACCAGCTGGGGTCAGGCTCTCTGGGTCTGAGTCCTGATTTCCCACTTTCCAGCTAGAGGAGCTTGAATGAGTCATTTAACTTCACGGTGCCTCAGTTTCCCCTCTCTAAAATGAGAATTATACCCATACCCACCTCTCAAACACCAAGTGCAGGCCTGGCTCAGAGCAGGTGCTGCAGCAATAGCTGCCATTGGTCAGCATCATCATCATGGTGGTAATGGTCCTACTTTGACTTTTGAGACAGAGTCTCACTCTGTCGCCCAGGCTGGAGTGCAGTGGTGCAATCTCGGCTCACTACAACCTCTGCCTCCCGGGTTCAAGTGATTCTTCTGCCTCAGTCTCCCAAGTAGTTGGGATTACAGGTGTGCGCCACCATGCCTGGCTAATTTTTGTGTTTTTAGTAGAGACAGGGTTTCACCATGTTGGCCATAACAATGGCTGTCCTTGAAGTAGTCCTGGGTACCAGGTGCCTTCAGTGACTTTTTTTTTTTTTTTTTTTTTGAGCTGGAGTCTTCCTCTGTCACCCAAGCTGGAGTGCAGTGGCACGATTTTGGCTCACTGCAACCTCTGCCTCCTGGGTTCATGCGATCCTCCTGCCTCAGCCTCCCAAGTAGCTGGGACTTGGGATACACTTGCCCCCGCTGGTCCTCCCTTCCACCTCTGTGAAGAGGAGGTCTCAAACTCCTGGCCTCAAGTGATCCACCCACCTCAGCCTCCCAAAGTGCTGGGATTTCAAGAGTGAGCCACCGCACCTGGCCCCTGTTTAGATGTTAGCATCAGTGACCCAGCACCTTGCTATGTGGCATGGATCCAAGCTTCGCCGGTGTTAATTAAGTTTCGATAGCTTGCCAAGCAAACAACTCAAATGTCCAACTGGCTGGGCATGGCCAGGTAGCCCATGCTGTGTCTGGACGTCCTTCTGCTGGTATAATTATTTTAAAATCACAGGACAGGGAAGGGCCCAGTGGCTCATACCTGTAATCCCAGCAATTTGGGAGGCCGAGGCGGGCGGATTACCTGAGGTCAGGAGATGGAGACCAGACTGGCCAATATGGTGAAACCCCGTCTCTACCAAAAATACAAAAATTAGCTGAGCCTGGTCGTGCATGCCTGGAATCCCAACTATTCGGGAGACTGAGGCAGGAGAATCGCTTGAACCCAGGAGGCGGAGATTGCAGCGAGCCAAGATCGTGCCACTGCACTCCAGCCTGGTTCCCAATAGACCCTGAAGGCCCTACAGGTTGTCTTCCCAACCTGCCCCTTGCTCCATACCACCCGCCTCCACCCCATAATATTATACAAGGACACCTAGTCAAACAAAATGATGCAACTTAATTTTATTAGGACAAGGCTGGTGGGCACTGGAGTGGCAACTTCCAGGGCCAGGAGAGGCACTGGGGAGGGGTCACAGGGATGCCACGTTAACTCTAGAGTCGCGGCCGCTTTACTTGTACAGCTCGTCCATGCCGAGAGTGATCCCGGCGGCGGTCACGAACTCCAGCAGGACCATGTGATCGCGCTTCTCGTTGGGGTCTTTGCTCAGGGCGGACTGGGTGCTCAGGTAGTGGTTGTCGGGCAGCAGCACGGGGCCGTCGCCGATGGGGGTGTTCTGCTGGTAGTGGTCGGCGAGCTGCACGCTGCCGTCCTCGATGTTGTGGCGGATCTTGAAGTTCACCTTGATGCCGTTCTTCTGCTTGTCGGCCATGATATAGACGTTGTGGCTGTTGTAGTTGTACTCCAGCTTGTGCCCCAGGATGTTGCCGTCCTCCTTGAAGTCGATGCCCTTCAGCTCGATGCGGTTCACCAGGGTGTCGCCCTCGAACTTCACCTCGGCGCGGGTCTTGTAGTTGCCGTCGTCCTTGAAGAAGATGGTGCGCTCCTGGACGTAGCCTTCGGGCATGGCGGACTTGAAGAAGTCGTGCTGCTTCATGTGGTCGGGGTAGCGGCTGAAGCACTGCACGCCGTAGGTCAGGGTGGTCACGAGGGTGGGCCAGGGCACGGGCAGCTTGCCGGTGGTGCAGATGAACTTCAGGGTCAGCTTGCCGTAGGTGGCATCGCCCTCGCCCTCGCCGGACACGCTGAACTTGTGGCCGTTTACGTCGCCGTCCAGCTCGACCAGGATGGGCACCACCCCGGTGAACAGCTCCTCGCCCTTGCTCACCATGGTGGCGACCGGATCTCCACAGCCTGTAGAGAAGCAGAATAGAGCCAAGTATGGGAGGAGGCAGTGAGGAGGGACCCCATGGCCAGCCCAGGCCCTCACCTCTTTTACTGATCCGGTGGTGCCCTCGTGCGCCCCGGCGCTGCTCCCTCCGCCGCCGCTCGGTGGGTCTCTGCCGGCTCGGTGGCCGCTCGGCTTTCCCTGGGGCCAGCGGTGGCCGCGGCCGGCCACGCTCTTCTTAACGGGCCGGGCCCCGCCTCCCGCCCCGCCCGCCGCCGTTCCCGGGGCCCGCCCTCTCGCCGGAGCCCCCGCCCCGCCCGCCGGCCTGGATCGCGCATTTCCTGGAATTCTGGACCCGGCGTCCTCGCCCGCCGACCCGAAAACTACGGCCCCGCGGCGCCCGACCGGACCCACCTGTGGGCGCGCGCTCCAGCCGCCTCGCTGGGCGCTGCCTTCCCCCCGGCTCCTCCAGCAACTTTCAAGCGTCCCCCTGCAGTCTGGGCGCTGCTGTGCGCCAGGCCCAAGCTAGAAGCGGGGACCCCGGGAGCATCGCAAGCCTCGAGGGCGCGCCTTAATTAACACGTGGTTTAAACGTCGACCATAGATAAAATAAAAGATTTTATTTAGTCTCCAGAAAAAGGGGGGAATGAAAGACCCCACCTGTAGGTTTGGCAAGCTAGCGATATCAGTTCGCTTCTCGCTTCTGTTCGCGCGCTTCTGCTCCCCGAGCTCAATAAAAGAGCCCACAACCCCTCACTCGGGGCGCCAGTCCTCCGATTGACTGAGTCGCCCGGGTACCCGTGTATCCAATAAACCCTCTTGCAGTTGCATCCGACTTGTGGTCTCGCTGTTCCTTGGGAGGGTCTCCTCTGAGTGATTGACTACCCGTCAGCGGGGGTCTTTCATTTGGGGGCTCGTCCGGGATCGCGGCCGCCACCGCGGTGGAGCTCCAATTCGCCCTATAGTGAGTCGTATTACGCGCGCTCACTGGCCGTCGTTTTACAACGTCGTGACTGGGAAAACCCTGGCGTTACCCAACTTAATCGCCTTGCAGCACATCCCCCTTTCGCCAGCTGGCGTAATAGCGAAGAGGCCCGCACCGATCGCCCTTCCCAACAGTTGCGCAGCCTGAATGGCGAATGGGACGCGCCCTGTAGCGGCGCATTAAGCGCGGCGGGTGTGGTGGTTACGCGCAGCGTGACCGCTACACTTGCCAGCGCCCTAGCGCCCGCTCCTTTCGCTTTCTTCCCTTCCTTTCTCGCCACGTTCGCCGGCTTTCCCCGTCAAGCTCTAAATCGGGGGCTCCCTTTAGGGTTCCGATTTAGTGCTTTACGGCACCTCGACCCCAAAAAACTTGATTAGGGTGATGGTTCACGTAGTGGGCCATCGCCCTGATAGACGGTTTTTCGCCCTTTGACGTTGGAGTCCACGTTCTTTAATAGTGGACTCTTGTTCCAAACTGGAACAACACTCAACCCTATCTCGGTCTATTCTTTTGATTTATAAGGGATTTTGCCGATTTCGGCCTATTGGTTAAAAAATGAGCTGATTTAACAAAAATTTAACGCGAATTTTAACAAAATATTAACGCTTACAATTTAGGTGGCACTTTTCGGGGAAATGTGCGCGGAACCCCTATTTGTTTATTTTTCTAAATACATTCAAATATGTATCCGCTCATGAGACAATAACCCTGATAAATGCTTCAATAATATTGAAAAAGGAAGAGTATGAGTATTCAACATTTCCGTGTCGCCCTTATTCCCTTTTTTGCGGCATTTTGCCTTCCTGTTTTTGCTCACCCAGAAACGCTGGTGAAAGTAAAAGATGCTGAAGATCAGTTGGGTGCACGAGTGGGTTACATCGAACTGGATCTCAACAGCGGTAAGATCCTTGAGAGTTTTCGCCCCGAAGAACGTTTTCCAATGATGAGCACTTTTAAAGTTCTGCTATGTGGCGCGGTATTATCCCGTATTGACGCCGGGCAAGAGCAACTCGGTCGCCGCATACACTATTCTCAGAATGACTTGGTTGAGTACTCACCAGTCACAGAAAAGCATCTTACGGATGGCATGACAGTAAGAGAATTATGCAGTGCTGCCATAACCATGAGTGATAACACTGCGGCCAACTTACTTCTGACAACGATCGGAGGACCGAAGGAGCTAACCGCTTTTTTGCACAACATGGGGGATCATGTAACTCGCCTTGATCGTTGGGAACCGGAGCTGAATGAAGCCATACCAAACGACGAGCGTGACACCACGATGCCTGTAGCAATGGCAACAACGTTGCGCAAACTATTAACTGGCGAACTACTTACTCTAGCTTCCCGGCAACAATTAATAGACTGGATGGAGGCGGATAAAGTTGCAGGACCACTTCTGCGCTCGGCCCTTCCGGCTGGCTGGTTTATTGCTGATAAATCTGGAGCCGGTGAGCGTGGGTCTCGCGGTATCATTGCAGCACTGGGGCCAGATGGTAAGCCCTCCCGTATCGTAGTTATCTACACGACGGGGAGTCAGGCAACTATGGATGAACGAAATAGACAGATCGCTGAGATAGGTGCCTCACTGATTAAGCATTGGTAACTGTCAGACCAAGTTTACTCATATATACTTTAGATTGATTTAAAACTTCATTTTTAATTTAAAAGGATCTAGGTGAAGATCCTTTTTGATAATCTCATGACCAAAATCCCTTAACGTGAGTTTTCGTTCCACTGAGCGTCAGACCCCGTAGAAAAGATCAAAGGATCTTCTTGAGATCCTTTTTTTCTGCGCGTAATCTGCTGCTTGCAAACAAAAAAACCACCGCTACCAGCGGTGGTTTGTTTGCCGGATCAAGAGCTACCAACTCTTTTTCCGAAGGTAACTGGCTTCAGCAGAGCGCAGATACCAAATACTGTCCTTCTAGTGTAGCCGTAGTTAGGCCACCACTTCAAGAACTCTGTAGCACCGCCTACATACCTCGCTCTGCTAATCCTGTTACCAGTGGCTGCTGCCAGTGGCGATAAGTCGTGTCTTACCGGGTTGGACTCAAGACGATAGTTACCGGATAAGGCGCAGCGGTCGGGCTGAACGGGGGGTTCGTGCACACAGCCCAGCTTGGAGCGAACGACCTACACCGAACTGAGATACCTACAGCGTGAGCTATGAGAAAGCGCCACGCTTCCCGAAGGGAGAAAGGCGGACAGGTATCCGGTAAGCGGCAGGGTCGGAACAGGAGAGCGCACGAGGGAGCTTCCAGGGGGAAACGCCTGGTATCTTTATAGTCCTGTCGGGTTTCGCCACCTCTGACTTGAGCGTCGATTTTTGTGATGCTCGTCAGGGGGGCGGAGCCTATGGAAAAACGCCAGCAACGCGGCCTTTTTACGGTTCCTGGCCTTTTGCTGGCCTTTTGCTCACATGTTCTTTCCTGCGTTATCCCCTGATTCTGTGGATAACCGTATTACCGCCTTTGAGTGAGCTGATACCGCTCGCCGCAGCCGAACGACCGAGCGCAGCGAGTCAGTGAGCGAGGAAGCGGAAGAGCGCCCAATACGCAAACCGCCTCTCCCCGCGCGTTGGCCGATTCATTAATGCAGCTGGCACGACAGGTTTCCCGACTGGAAAGCGGGCAGTGAGCGCAACGCAATTAATGTGAGTTAGCTCACTCATTAGGCACCCCAGGCTTTACACTTTATGCTTCCGGCTCGTATGTTGTGTGGAATTGTGAGCGGATAACAATTTCACACAGGAAACAGCTATGACCATGATTACGCCAAGCGCGCAATTAACCCTCACTAAAGGGAACAAAAGCTGGGTACCGGGCCCCCCCTCGAGAGTC

**SiLHGA**

8395 bp

5’LTR CMV promoter 1-531

R region 532-590

U5 region 596-666

Packaging signal 667-1023

ADA LCR 3331-1075 (reverse orientation)

hGH pA 3937-3353 (reverse orientation)

eGFP gene 4674-3955 (reverse orientation)

ADA promoter 5130-4688 (reverse orientation)

3’LTR DU3 region 5213-5330

R region 5331-5397

U5 region 5398-5474

CGTTACATAACTTACGGTAAATGGCCCGCCTGGCTGACCGCCCAACGACCCCCGCCCATTGACGTCAATAATGACGTATGTTCCCATAGTAACGCCAATAGGGACTTTCCATTGACGTCAATGGGTGGAGTATTTACGGTAAACTGCCCACTTGGCAGTACATCAAGTGTATCATATGCCAAGTACGCCCCCTATTGACGTCAATGACGGTAAATGGCCCGCCTGGCATTATGCCCAGTACATGACCTTATGGGACTTTCCTACTTGGCAGTACATCTACGTATTAGTCATCGCTATTACCATGGTGATGCGGTTTTGGCAGTACATCAATGGGCGTGGATAGCGGTTTGACTCACGGGGATTTCCAAGTCTCCACCCCATTGACGTCAATGGGAGTTTGTTTTGGCACCAAAATCAACGGGACTTTCCAAAATGTCGTAACAACTCCGCCCCATTGACGCAAATGGGCGGTAGGCGTGTACGGTGGGAGGTCTATATAAGCAGAGCTCGTTTAGTGAACCGCGCCAGTCTTCCGATAGACTGCGTCGCCCGGGTACCCGTATTCCCAATAAAGCCTCTTGCTGTTTGCATCCGAATCGTGGTCTCGCTGTTCCTTGGGAGGGTCTCCTCTGAGTGATTGACTACCCACGACGGGGGTCTTTCATTTGGGGGCTCGTCCGGGATTTGGAGACCCCTGCCCAGGGACCACCGACCCACCACCGGGAGGTAAGCTGGCCAGCAACTTATCTGTGTCTGTCCGATTGTCTAGTGTCTATGTTTGATGTTATGCGCCTGCGTCTGTACTAGTTAGCTAACTAGCTCTGTATCTGGCGGACCCGTGGTGGAACTGACGAGTTCTGAACACCCGGCCGCAACCCTGGGAGACGTCCCAGGGACTTTGGGGGCCGTTTTTGTGGCCCGACCTGAGGAAGGGAGTCGATGTGGAATCCGACCCCGTCAGGATATGTGGTTCTGGTAGGAGACGAGAACCTAAAACAGTTCCCGCCTCCGTCTGAATTTTTGCTAAGCTCTAGAGTCGACACGCGTGCGATTAACACCGGCGAAGCTTGGATCCATGCCACATAGCAAGGTGCTGGGTCACTGATGCTAACATCTAAACAGGGGCCAGGTGCGGTGGCTCACTCTTGAAATCCCAGCACTTTGGGAGGCTGAGGTGGGTGGATCACTTGAGGCCAGGAGTTTGAGACCTCCTCTTCACAGAGGTGGAAGGGAGGACCAGCGGGGGCAAGTGTATCCCAAGTCCCAGCTACTTGGGAGGCTGAGGCAGGAGGATCGCATGAACCCAGGAGGCAGAGGTTGCAGTGAGCCAAAATCGTGCCACTGCACTCCAGCTTGGGTGACAGAGGAAGACTCCAGCTCAAAAAAAAAAAAAAAAAAAAAGTCACTGAAGGCACCTGGTACCCAGGACTACTTCAAGGACAGCCATTGTTATGGCCAACATGGTGAAACCCTGTCTCTACTAAAAACACAAAAATTAGCCAGGCATGGTGGCGCACACCTGTAATCCCAACTACTTGGGAGACTGAGGCAGAAGAATCACTTGAACCCGGGAGGCAGAGGTTGTAGTGAGCCGAGATTGCACCACTGCACTCCAGCCTGGGCGACAGAGTGAGACTCTGTCTCAAAAGTCAAAGTAGGACCATTACCACCATGATGATGATGCTGACCAATGGCAGCTATTGCTGCAGCACCTGCTCTGAGCCAGGCCTGCACTTGGTGTTTGAGAGGTGGGTATGGGTATAATTCTCATTTTAGAGAGGGGAAACTGAGGCACCGTGAAGTTAAATGACTCATTCAAGCTCCTCTAGCTGGAAAGTGGGAAATCAGGACTCAGACCCAGAGAGCCTGACCCCAGCTGGTGTCCAAGTTCAAATTCCTTGCTGTTCTAGCTCCCTGGTCACTAGGAGTGCAGAGGTTATCCCCCACAACTGCTCAGGGGCCTGAGAACATTAAGGGCTGAGGGCAAGCCTACCCCCAACACCTTGGGTAAATACAGATTCCAACCTCACTCTGCGCCCACAGCCTGCTCCTCTCCACCTCCCCAGCCAGACCATGGTCCTACTACCTTGCCCCGCCCAGGGGGTACCACAGGGACCCCTTCAAGACACCTGAGCAGGTGGACAAGTCTCTCCTGGATCACAGGGCCCCCAGCCACCCAGGTCAAGCCCACCCTGAGGTTGAATGCAGGATTGGGAAGCTGCCTGGCCTCCCCTGCCTCCCTGTGTGAACACAGACCACTTCCTCTCTCTGATCCTCCCTTCCTCCTCCGAAGCAAAAGGCTTCGATGAAACTCAGTCTCCTTTGTTCCCCTCCACCACCAACTGCCATGTGAGTCAAAATGGGTAAGGCAGGCTCTTCCTAGGCACATTTTTGTTTTAACAGGTGGCTCTGGTAACTGAAAGCCACTGCAAGCCCAAAGAAGTGCAGCTGGTGGGTTTTGTGTCAGGAAGACAAAAAGGAGGAGGGAGGAGGGAGATTCTGCAGCTGGAGAGGCCTCCCAGAGTCTACTGCAACCCCCACTCCAGTCCCCAGGAAGGAAACGCACCCCTGAGGGCCTGCTGTTACCAGCCAGGCACTGTACAAAAACCAATCAGCATGTCAGTTTCCCCAAGAACCCCAAGATCTTGGTTTTGTCACCCCATTTTACAGAGGGGGAAGCTGAGTTTCAGGAGTTGTCTACCTGAGGTACCCTAGCTGCTAAGCGGCAGGCAGGAATTCAACCCCAGTACATCTGAAGCCCAGGGCTAATGCACTGGCCCACCCTGCCACCCTGATTCTAACTCTTCCCCCCACACTGCCCCCTCTTTTTTGCATAAAGTGGAATTGGCAAATGAGCAAAGCAATGAACAGCTTCTACTTGAGTGCAGGCAGATCTGGGTTCAAGTCTAGCTCTGCTGCTTGCCAGCTGTGTGACTTTGGCAAGTTCCCTCCCCTCTCTGAGTCTCAGTTTCCTCATAATAAGTGAAGTTAATAATAGGACAGGACCTTCCTCAGAGGGCTGACATAAACATTAAATGAGTCACTGTATGCAAAGCAGGACAGCGCCTCGTGCAGAGTAGACATTCAAATATGAGACAGAAAAGGCACATTGCTGATTTAGCCCCAATTCCCCTCTGCATCAGAGAGGGACAGTTTAGAAACTGCTGTCCCTTGGCCGGGTGCGGTGGCTCATGCCTGTAATCCCGGCACTTTGGGAGGCTGAGGTGGGCGGATCACCTGAAGTCGGGAGTTCGAGACCAGCCTGGCCAACAAGGAGAAACCCCGTCTCTACTAAAAATACAAAAAATTAGCCGGGCATGGTGGTGCATGCCTTAAGTATCGATACACCGATAGCTTGCCAAGCAAACAACTCAAATGTCCAACTGGCTGGGCATGGCCAGGTAGCCCATGCTGTGTCTGGACGTCCTTCTGCTGGTATAATTATTTTAAAATCACAGGACAGGGAAGGGCCCAGTGGCTCATACCTGTAATCCCAGCAATTTGGGAGGCCGAGGCGGGCGGATTACCTGAGGTCAGGAGATGGAGACCAGACTGGCCAATATGGTGAAACCCCGTCTCTACCAAAAATACAAAAATTAGCTGAGCCTGGTCGTGCATGCCTGGAATCCCAACTATTCGGGAGACTGAGGCAGGAGAATCGCTTGAACCCAGGAGGCGGAGATTGCAGCGAGCCAAGATCGTGCCACTGCACTCCAGCTTGGTTCCCAATAGACCCCGCAGGCCCTACAGGTTGCCTTCCCAACTTGCCCCTTGCTCCATACCACCCCCCTCCACCCCATAATATTATAGAAGGACACCTAGTCAGACAAAATGATGCAACTTAATTTTATTAGGACAAGGCTGGTGGGCACTGGAGTGGCAACTTCCAGGGCCAGGAGAGGCACTGGGGAGGGGTCACAGGGATGCCACGTTAACTCTAGAGTCGCGGCCGCTTTACTTGTACAGCTCGTCCATGCCGAGAGTGATCCCGGCGGCGGTCACGAACTCCAGCAGGACCATGTGATCGCGCTTCTCGTTGGGGTCTTTGCTCAGGGCGGACTGGGTGCTCAGGTAGTGGTTGTCGGGCAGCAGCACGGGGCCGTCGCCGATGGGGGTGTTCTGCTGGTAGTGGTCGGCGAGCTGCACGCTGCCGTCCTCGATGTTGTGGCGGATCTTGAAGTTCACCTTGATGCCGTTCTTCTGCTTGTCGGCCATGATATAGACGTTGTGGCTGTTGTAGTTGTACTCCAGCTTGTGCCCCAGGATGTTGCCGTCCTCCTTGAAGTCGATGCCCTTCAGCTCGATGCGGTTCACCAGGGTGTCGCCCTCGAACTTCACCTCGGCGCGGGTCTTGTAGTTGCCGTCGTCCTTGAAGAAGATGGTGCGCTCCTGGACGTAGCCTTCGGGCATGGCGGACTTGAAGAAGTCGTGCTGCTTCATGTGGTCGGGGTAGCGGCTGAAGCACTGCACGCCGTAGGTCAGGGTGGTCACGAGGGTGGGCCAGGGCACGGGCAGCTTGCCGGTGGTGCAGATGAACTTCAGGGTCAGCTTGCCGTAGGTGGCATCGCCCTCGCCCTCGCCGGACACGCTGAACTTGTGGCCGTTTACGTCGCCGTCCAGCTCGACCAGGATGGGCACCACCCCGGTGAACAGCTCCTCGCCCTTGCTCACCATGGTGGCGACCGGTGGTGCCCTCGTGCGCCCCGGCGCTGCTCCCTCCGCCGCCGCTCGGTGGGTCTCTGCCGGCTCGGTGGCCGCTCGGCTTTCCCTGGGGCCAGCGGTGGCCGCGGCCGGCCACGCTCTTCTTAACGGGCCGGGCCCCGCCTCCCGCCCCGCCCGCCGCCGTTCCCGGGGCCCGCCCTCTCGCCGGAGCCCCCGCCCCGCCCGCCGGCCTGGATCGCGCATTTCCTGGAATTCTGGACCCGGCGTCCTCGCCCGCCGACCCGAAAACTACGGCCCCGCGGCGCCCGACCGGACCCACCTGTGGGCGCGCGCTCCAGCCGCCTCGCTGGGCGCTGCCTTCCCCCCGGCTCCTCCAGCAACTTTCAAGCGTCCCCCTGCAGTCTGGGCGCTGCTGTGCGCCAGGCCCAAGCTAGAAGCGGGGACCCCGGGAGCATCGCAAGCCTCGAGGGCGCGCCTTAATTAACACGTGGTTTAAACGTCGACCATAGATAAAATAAAAGATTTTATTTAGTCTCCAGAAAAAGGGGGGAATGAAAGACCCCACCTGTAGGTTTGGCAAGCTAGCGATATCAGTTCGCTTCTCGCTTCTGTTCGCGCGCTTCTGCTCCCCGAGCTCAATAAAAGAGCCCACAACCCCTCACTCGGGGCGCCAGTCCTCCGATTGACTGAGTCGCCCGGGTACCCGTGTATCCAATAAACCCTCTTGCAGTTGCATCCGACTTGTGGTCTCGCTGTTCCTTGGGAGGGTCTCCTCTGAGTGATTGACTACCCGTCAGCGGGGGTCTTTCATTTGGGGGCTCGTCCGGGATCGCGGCCGCCACCGCGGTGGAGCTCCAATTCGCCCTATAGTGAGTCGTATTACGCGCGCTCACTGGCCGTCGTTTTACAACGTCGTGACTGGGAAAACCCTGGCGTTACCCAACTTAATCGCCTTGCAGCACATCCCCCTTTCGCCAGCTGGCGTAATAGCGAAGAGGCCCGCACCGATCGCCCTTCCCAACAGTTGCGCAGCCTGAATGGCGAATGGGACGCGCCCTGTAGCGGCGCATTAAGCGCGGCGGGTGTGGTGGTTACGCGCAGCGTGACCGCTACACTTGCCAGCGCCCTAGCGCCCGCTCCTTTCGCTTTCTTCCCTTCCTTTCTCGCCACGTTCGCCGGCTTTCCCCGTCAAGCTCTAAATCGGGGGCTCCCTTTAGGGTTCCGATTTAGTGCTTTACGGCACCTCGACCCCAAAAAACTTGATTAGGGTGATGGTTCACGTAGTGGGCCATCGCCCTGATAGACGGTTTTTCGCCCTTTGACGTTGGAGTCCACGTTCTTTAATAGTGGACTCTTGTTCCAAACTGGAACAACACTCAACCCTATCTCGGTCTATTCTTTTGATTTATAAGGGATTTTGCCGATTTCGGCCTATTGGTTAAAAAATGAGCTGATTTAACAAAAATTTAACGCGAATTTTAACAAAATATTAACGCTTACAATTTAGGTGGCACTTTTCGGGGAAATGTGCGCGGAACCCCTATTTGTTTATTTTTCTAAATACATTCAAATATGTATCCGCTCATGAGACAATAACCCTGATAAATGCTTCAATAATATTGAAAAAGGAAGAGTATGAGTATTCAACATTTCCGTGTCGCCCTTATTCCCTTTTTTGCGGCATTTTGCCTTCCTGTTTTTGCTCACCCAGAAACGCTGGTGAAAGTAAAAGATGCTGAAGATCAGTTGGGTGCACGAGTGGGTTACATCGAACTGGATCTCAACAGCGGTAAGATCCTTGAGAGTTTTCGCCCCGAAGAACGTTTTCCAATGATGAGCACTTTTAAAGTTCTGCTATGTGGCGCGGTATTATCCCGTATTGACGCCGGGCAAGAGCAACTCGGTCGCCGCATACACTATTCTCAGAATGACTTGGTTGAGTACTCACCAGTCACAGAAAAGCATCTTACGGATGGCATGACAGTAAGAGAATTATGCAGTGCTGCCATAACCATGAGTGATAACACTGCGGCCAACTTACTTCTGACAACGATCGGAGGACCGAAGGAGCTAACCGCTTTTTTGCACAACATGGGGGATCATGTAACTCGCCTTGATCGTTGGGAACCGGAGCTGAATGAAGCCATACCAAACGACGAGCGTGACACCACGATGCCTGTAGCAATGGCAACAACGTTGCGCAAACTATTAACTGGCGAACTACTTACTCTAGCTTCCCGGCAACAATTAATAGACTGGATGGAGGCGGATAAAGTTGCAGGACCACTTCTGCGCTCGGCCCTTCCGGCTGGCTGGTTTATTGCTGATAAATCTGGAGCCGGTGAGCGTGGGTCTCGCGGTATCATTGCAGCACTGGGGCCAGATGGTAAGCCCTCCCGTATCGTAGTTATCTACACGACGGGGAGTCAGGCAACTATGGATGAACGAAATAGACAGATCGCTGAGATAGGTGCCTCACTGATTAAGCATTGGTAACTGTCAGACCAAGTTTACTCATATATACTTTAGATTGATTTAAAACTTCATTTTTAATTTAAAAGGATCTAGGTGAAGATCCTTTTTGATAATCTCATGACCAAAATCCCTTAACGTGAGTTTTCGTTCCACTGAGCGTCAGACCCCGTAGAAAAGATCAAAGGATCTTCTTGAGATCCTTTTTTTCTGCGCGTAATCTGCTGCTTGCAAACAAAAAAACCACCGCTACCAGCGGTGGTTTGTTTGCCGGATCAAGAGCTACCAACTCTTTTTCCGAAGGTAACTGGCTTCAGCAGAGCGCAGATACCAAATACTGTCCTTCTAGTGTAGCCGTAGTTAGGCCACCACTTCAAGAACTCTGTAGCACCGCCTACATACCTCGCTCTGCTAATCCTGTTACCAGTGGCTGCTGCCAGTGGCGATAAGTCGTGTCTTACCGGGTTGGACTCAAGACGATAGTTACCGGATAAGGCGCAGCGGTCGGGCTGAACGGGGGGTTCGTGCACACAGCCCAGCTTGGAGCGAACGACCTACACCGAACTGAGATACCTACAGCGTGAGCTATGAGAAAGCGCCACGCTTCCCGAAGGGAGAAAGGCGGACAGGTATCCGGTAAGCGGCAGGGTCGGAACAGGAGAGCGCACGAGGGAGCTTCCAGGGGGAAACGCCTGGTATCTTTATAGTCCTGTCGGGTTTCGCCACCTCTGACTTGAGCGTCGATTTTTGTGATGCTCGTCAGGGGGGCGGAGCCTATGGAAAAACGCCAGCAACGCGGCCTTTTTACGGTTCCTGGCCTTTTGCTGGCCTTTTGCTCACATGTTCTTTCCTGCGTTATCCCCTGATTCTGTGGATAACCGTATTACCGCCTTTGAGTGAGCTGATACCGCTCGCCGCAGCCGAACGACCGAGCGCAGCGAGTCAGTGAGCGAGGAAGCGGAAGAGCGCCCAATACGCAAACCGCCTCTCCCCGCGCGTTGGCCGATTCATTAATGCAGCTGGCACGACAGGTTTCCCGACTGGAAAGCGGGCAGTGAGCGCAACGCAATTAATGTGAGTTAGCTCACTCATTAGGCACCCCAGGCTTTACACTTTATGCTTCCGGCTCGTATGTTGTGTGGAATTGTGAGCGGATAACAATTTCACACAGGAAACAGCTATGACCATGATTACGCCAAGCGCGCAATTAACCCTCACTAAAGGGAACAAAAGCTGGGTACCGGGCCCCCCCTCGAGAGTC

**SiLHGI7A**

8499 bp

5’LTR CMV promoter 1-531

R region 532-590

U5 region 596-666

Packaging signal 667-1023

ADA LCR 3331-1075 (reverse orientation)

hGH pA 3937-3353 (reverse orientation)

eGFP gene 4674-3955 (reverse orientation)

Intron 7 4773-4698 (reverse orientation)

ADA promoter 5234-4792 (reverse orientation)

3’LTR DU3 region 5317-5434

R region 5435-5501

U5 region 5502-5578

CGTTACATAACTTACGGTAAATGGCCCGCCTGGCTGACCGCCCAACGACCCCCGCCCATTGACGTCAATAATGACGTATGTTCCCATAGTAACGCCAATAGGGACTTTCCATTGACGTCAATGGGTGGAGTATTTACGGTAAACTGCCCACTTGGCAGTACATCAAGTGTATCATATGCCAAGTACGCCCCCTATTGACGTCAATGACGGTAAATGGCCCGCCTGGCATTATGCCCAGTACATGACCTTATGGGACTTTCCTACTTGGCAGTACATCTACGTATTAGTCATCGCTATTACCATGGTGATGCGGTTTTGGCAGTACATCAATGGGCGTGGATAGCGGTTTGACTCACGGGGATTTCCAAGTCTCCACCCCATTGACGTCAATGGGAGTTTGTTTTGGCACCAAAATCAACGGGACTTTCCAAAATGTCGTAACAACTCCGCCCCATTGACGCAAATGGGCGGTAGGCGTGTACGGTGGGAGGTCTATATAAGCAGAGCTCGTTTAGTGAACCGCGCCAGTCTTCCGATAGACTGCGTCGCCCGGGTACCCGTATTCCCAATAAAGCCTCTTGCTGTTTGCATCCGAATCGTGGTCTCGCTGTTCCTTGGGAGGGTCTCCTCTGAGTGATTGACTACCCACGACGGGGGTCTTTCATTTGGGGGCTCGTCCGGGATTTGGAGACCCCTGCCCAGGGACCACCGACCCACCACCGGGAGGTAAGCTGGCCAGCAACTTATCTGTGTCTGTCCGATTGTCTAGTGTCTATGTTTGATGTTATGCGCCTGCGTCTGTACTAGTTAGCTAACTAGCTCTGTATCTGGCGGACCCGTGGTGGAACTGACGAGTTCTGAACACCCGGCCGCAACCCTGGGAGACGTCCCAGGGACTTTGGGGGCCGTTTTTGTGGCCCGACCTGAGGAAGGGAGTCGATGTGGAATCCGACCCCGTCAGGATATGTGGTTCTGGTAGGAGACGAGAACCTAAAACAGTTCCCGCCTCCGTCTGAATTTTTGCTAAGCTCTAGAGTCGACACGCGTGCGATTAACACCGGCGAAGCTTGGATCCATGCCACATAGCAAGGTGCTGGGTCACTGATGCTAACATCTAAACAGGGGCCAGGTGCGGTGGCTCACTCTTGAAATCCCAGCACTTTGGGAGGCTGAGGTGGGTGGATCACTTGAGGCCAGGAGTTTGAGACCTCCTCTTCACAGAGGTGGAAGGGAGGACCAGCGGGGGCAAGTGTATCCCAAGTCCCAGCTACTTGGGAGGCTGAGGCAGGAGGATCGCATGAACCCAGGAGGCAGAGGTTGCAGTGAGCCAAAATCGTGCCACTGCACTCCAGCTTGGGTGACAGAGGAAGACTCCAGCTCAAAAAAAAAAAAAAAAAAAAAGTCACTGAAGGCACCTGGTACCCAGGACTACTTCAAGGACAGCCATTGTTATGGCCAACATGGTGAAACCCTGTCTCTACTAAAAACACAAAAATTAGCCAGGCATGGTGGCGCACACCTGTAATCCCAACTACTTGGGAGACTGAGGCAGAAGAATCACTTGAACCCGGGAGGCAGAGGTTGTAGTGAGCCGAGATTGCACCACTGCACTCCAGCCTGGGCGACAGAGTGAGACTCTGTCTCAAAAGTCAAAGTAGGACCATTACCACCATGATGATGATGCTGACCAATGGCAGCTATTGCTGCAGCACCTGCTCTGAGCCAGGCCTGCACTTGGTGTTTGAGAGGTGGGTATGGGTATAATTCTCATTTTAGAGAGGGGAAACTGAGGCACCGTGAAGTTAAATGACTCATTCAAGCTCCTCTAGCTGGAAAGTGGGAAATCAGGACTCAGACCCAGAGAGCCTGACCCCAGCTGGTGTCCAAGTTCAAATTCCTTGCTGTTCTAGCTCCCTGGTCACTAGGAGTGCAGAGGTTATCCCCCACAACTGCTCAGGGGCCTGAGAACATTAAGGGCTGAGGGCAAGCCTACCCCCAACACCTTGGGTAAATACAGATTCCAACCTCACTCTGCGCCCACAGCCTGCTCCTCTCCACCTCCCCAGCCAGACCATGGTCCTACTACCTTGCCCCGCCCAGGGGGTACCACAGGGACCCCTTCAAGACACCTGAGCAGGTGGACAAGTCTCTCCTGGATCACAGGGCCCCCAGCCACCCAGGTCAAGCCCACCCTGAGGTTGAATGCAGGATTGGGAAGCTGCCTGGCCTCCCCTGCCTCCCTGTGTGAACACAGACCACTTCCTCTCTCTGATCCTCCCTTCCTCCTCCGAAGCAAAAGGCTTCGATGAAACTCAGTCTCCTTTGTTCCCCTCCACCACCAACTGCCATGTGAGTCAAAATGGGTAAGGCAGGCTCTTCCTAGGCACATTTTTGTTTTAACAGGTGGCTCTGGTAACTGAAAGCCACTGCAAGCCCAAAGAAGTGCAGCTGGTGGGTTTTGTGTCAGGAAGACAAAAAGGAGGAGGGAGGAGGGAGATTCTGCAGCTGGAGAGGCCTCCCAGAGTCTACTGCAACCCCCACTCCAGTCCCCAGGAAGGAAACGCACCCCTGAGGGCCTGCTGTTACCAGCCAGGCACTGTACAAAAACCAATCAGCATGTCAGTTTCCCCAAGAACCCCAAGATCTTGGTTTTGTCACCCCATTTTACAGAGGGGGAAGCTGAGTTTCAGGAGTTGTCTACCTGAGGTACCCTAGCTGCTAAGCGGCAGGCAGGAATTCAACCCCAGTACATCTGAAGCCCAGGGCTAATGCACTGGCCCACCCTGCCACCCTGATTCTAACTCTTCCCCCCACACTGCCCCCTCTTTTTTGCATAAAGTGGAATTGGCAAATGAGCAAAGCAATGAACAGCTTCTACTTGAGTGCAGGCAGATCTGGGTTCAAGTCTAGCTCTGCTGCTTGCCAGCTGTGTGACTTTGGCAAGTTCCCTCCCCTCTCTGAGTCTCAGTTTCCTCATAATAAGTGAAGTTAATAATAGGACAGGACCTTCCTCAGAGGGCTGACATAAACATTAAATGAGTCACTGTATGCAAAGCAGGACAGCGCCTCGTGCAGAGTAGACATTCAAATATGAGACAGAAAAGGCACATTGCTGATTTAGCCCCAATTCCCCTCTGCATCAGAGAGGGACAGTTTAGAAACTGCTGTCCCTTGGCCGGGTGCGGTGGCTCATGCCTGTAATCCCGGCACTTTGGGAGGCTGAGGTGGGCGGATCACCTGAAGTCGGGAGTTCGAGACCAGCCTGGCCAACAAGGAGAAACCCCGTCTCTACTAAAAATACAAAAAATTAGCCGGGCATGGTGGTGCATGCCTTAAGTATCGATACACCGATAGCTTGCCAAGCAAACAACTCAAATGTCCAACTGGCTGGGCATGGCCAGGTAGCCCATGCTGTGTCTGGACGTCCTTCTGCTGGTATAATTATTTTAAAATCACAGGACAGGGAAGGGCCCAGTGGCTCATACCTGTAATCCCAGCAATTTGGGAGGCCGAGGCGGGCGGATTACCTGAGGTCAGGAGATGGAGACCAGACTGGCCAATATGGTGAAACCCCGTCTCTACCAAAAATACAAAAATTAGCTGAGCCTGGTCGTGCATGCCTGGAATCCCAACTATTCGGGAGACTGAGGCAGGAGAATCGCTTGAACCCAGGAGGCGGAGATTGCAGCGAGCCAAGATCGTGCCACTGCACTCCAGCCTGGTTCCCAATAGACCCTGAAGGCCCTACAGGTTGTCTTCCCAACCTGCCCCTTGCTCCATACCACCCGCCTCCACCCCATAATATTATACAAGGACACCTAGTCAAACAAAATGATGCAACTTAATTTTATTAGGACAAGGCTGGTGGGCACTGGAGTGGCAACTTCCAGGGCCAGGAGAGGCACTGGGGAGGGGTCACAGGGATGCCACGTTAACTCTAGAGTCGCGGCCGCTTTACTTGTACAGCTCGTCCATGCCGAGAGTGATCCCGGCGGCGGTCACGAACTCCAGCAGGACCATGTGATCGCGCTTCTCGTTGGGGTCTTTGCTCAGGGCGGACTGGGTGCTCAGGTAGTGGTTGTCGGGCAGCAGCACGGGGCCGTCGCCGATGGGGGTGTTCTGCTGGTAGTGGTCGGCGAGCTGCACGCTGCCGTCCTCGATGTTGTGGCGGATCTTGAAGTTCACCTTGATGCCGTTCTTCTGCTTGTCGGCCATGATATAGACGTTGTGGCTGTTGTAGTTGTACTCCAGCTTGTGCCCCAGGATGTTGCCGTCCTCCTTGAAGTCGATGCCCTTCAGCTCGATGCGGTTCACCAGGGTGTCGCCCTCGAACTTCACCTCGGCGCGGGTCTTGTAGTTGCCGTCGTCCTTGAAGAAGATGGTGCGCTCCTGGACGTAGCCTTCGGGCATGGCGGACTTGAAGAAGTCGTGCTGCTTCATGTGGTCGGGGTAGCGGCTGAAGCACTGCACGCCGTAGGTCAGGGTGGTCACGAGGGTGGGCCAGGGCACGGGCAGCTTGCCGGTGGTGCAGATGAACTTCAGGGTCAGCTTGCCGTAGGTGGCATCGCCCTCGCCCTCGCCGGACACGCTGAACTTGTGGCCGTTTACGTCGCCGTCCAGCTCGACCAGGATGGGCACCACCCCGGTGAACAGCTCCTCGCCCTTGCTCACCATGGTGGCGACCGGATCTCCACAGCCTGTAGAGAAGCAGAATAGAGCCAAGTATGGGAGGAGGCAGTGAGGAGGGACCCCATGGCCAGCCCAGGCCCTCACCTCTTTTACTGATCCGGTGGTGCCCTCGTGCGCCCCGGCGCTGCTCCCTCCGCCGCCGCTCGGTGGGTCTCTGCCGGCTCGGTGGCCGCTCGGCTTTCCCTGGGGCCAGCGGTGGCCGCGGCCGGCCACGCTCTTCTTAACGGGCCGGGCCCCGCCTCCCGCCCCGCCCGCCGCCGTTCCCGGGGCCCGCCCTCTCGCCGGAGCCCCCGCCCCGCCCGCCGGCCTGGATCGCGCATTTCCTGGAATTCTGGACCCGGCGTCCTCGCCCGCCGACCCGAAAACTACGGCCCCGCGGCGCCCGACCGGACCCACCTGTGGGCGCGCGCTCCAGCCGCCTCGCTGGGCGCTGCCTTCCCCCCGGCTCCTCCAGCAACTTTCAAGCGTCCCCCTGCAGTCTGGGCGCTGCTGTGCGCCAGGCCCAAGCTAGAAGCGGGGACCCCGGGAGCATCGCAAGCCTCGAGGGCGCGCCTTAATTAACACGTGGTTTAAACGTCGACCATAGATAAAATAAAAGATTTTATTTAGTCTCCAGAAAAAGGGGGGAATGAAAGACCCCACCTGTAGGTTTGGCAAGCTAGCGATATCAGTTCGCTTCTCGCTTCTGTTCGCGCGCTTCTGCTCCCCGAGCTCAATAAAAGAGCCCACAACCCCTCACTCGGGGCGCCAGTCCTCCGATTGACTGAGTCGCCCGGGTACCCGTGTATCCAATAAACCCTCTTGCAGTTGCATCCGACTTGTGGTCTCGCTGTTCCTTGGGAGGGTCTCCTCTGAGTGATTGACTACCCGTCAGCGGGGGTCTTTCATTTGGGGGCTCGTCCGGGATCGCGGCCGCCACCGCGGTGGAGCTCCAATTCGCCCTATAGTGAGTCGTATTACGCGCGCTCACTGGCCGTCGTTTTACAACGTCGTGACTGGGAAAACCCTGGCGTTACCCAACTTAATCGCCTTGCAGCACATCCCCCTTTCGCCAGCTGGCGTAATAGCGAAGAGGCCCGCACCGATCGCCCTTCCCAACAGTTGCGCAGCCTGAATGGCGAATGGGACGCGCCCTGTAGCGGCGCATTAAGCGCGGCGGGTGTGGTGGTTACGCGCAGCGTGACCGCTACACTTGCCAGCGCCCTAGCGCCCGCTCCTTTCGCTTTCTTCCCTTCCTTTCTCGCCACGTTCGCCGGCTTTCCCCGTCAAGCTCTAAATCGGGGGCTCCCTTTAGGGTTCCGATTTAGTGCTTTACGGCACCTCGACCCCAAAAAACTTGATTAGGGTGATGGTTCACGTAGTGGGCCATCGCCCTGATAGACGGTTTTTCGCCCTTTGACGTTGGAGTCCACGTTCTTTAATAGTGGACTCTTGTTCCAAACTGGAACAACACTCAACCCTATCTCGGTCTATTCTTTTGATTTATAAGGGATTTTGCCGATTTCGGCCTATTGGTTAAAAAATGAGCTGATTTAACAAAAATTTAACGCGAATTTTAACAAAATATTAACGCTTACAATTTAGGTGGCACTTTTCGGGGAAATGTGCGCGGAACCCCTATTTGTTTATTTTTCTAAATACATTCAAATATGTATCCGCTCATGAGACAATAACCCTGATAAATGCTTCAATAATATTGAAAAAGGAAGAGTATGAGTATTCAACATTTCCGTGTCGCCCTTATTCCCTTTTTTGCGGCATTTTGCCTTCCTGTTTTTGCTCACCCAGAAACGCTGGTGAAAGTAAAAGATGCTGAAGATCAGTTGGGTGCACGAGTGGGTTACATCGAACTGGATCTCAACAGCGGTAAGATCCTTGAGAGTTTTCGCCCCGAAGAACGTTTTCCAATGATGAGCACTTTTAAAGTTCTGCTATGTGGCGCGGTATTATCCCGTATTGACGCCGGGCAAGAGCAACTCGGTCGCCGCATACACTATTCTCAGAATGACTTGGTTGAGTACTCACCAGTCACAGAAAAGCATCTTACGGATGGCATGACAGTAAGAGAATTATGCAGTGCTGCCATAACCATGAGTGATAACACTGCGGCCAACTTACTTCTGACAACGATCGGAGGACCGAAGGAGCTAACCGCTTTTTTGCACAACATGGGGGATCATGTAACTCGCCTTGATCGTTGGGAACCGGAGCTGAATGAAGCCATACCAAACGACGAGCGTGACACCACGATGCCTGTAGCAATGGCAACAACGTTGCGCAAACTATTAACTGGCGAACTACTTACTCTAGCTTCCCGGCAACAATTAATAGACTGGATGGAGGCGGATAAAGTTGCAGGACCACTTCTGCGCTCGGCCCTTCCGGCTGGCTGGTTTATTGCTGATAAATCTGGAGCCGGTGAGCGTGGGTCTCGCGGTATCATTGCAGCACTGGGGCCAGATGGTAAGCCCTCCCGTATCGTAGTTATCTACACGACGGGGAGTCAGGCAACTATGGATGAACGAAATAGACAGATCGCTGAGATAGGTGCCTCACTGATTAAGCATTGGTAACTGTCAGACCAAGTTTACTCATATATACTTTAGATTGATTTAAAACTTCATTTTTAATTTAAAAGGATCTAGGTGAAGATCCTTTTTGATAATCTCATGACCAAAATCCCTTAACGTGAGTTTTCGTTCCACTGAGCGTCAGACCCCGTAGAAAAGATCAAAGGATCTTCTTGAGATCCTTTTTTTCTGCGCGTAATCTGCTGCTTGCAAACAAAAAAACCACCGCTACCAGCGGTGGTTTGTTTGCCGGATCAAGAGCTACCAACTCTTTTTCCGAAGGTAACTGGCTTCAGCAGAGCGCAGATACCAAATACTGTCCTTCTAGTGTAGCCGTAGTTAGGCCACCACTTCAAGAACTCTGTAGCACCGCCTACATACCTCGCTCTGCTAATCCTGTTACCAGTGGCTGCTGCCAGTGGCGATAAGTCGTGTCTTACCGGGTTGGACTCAAGACGATAGTTACCGGATAAGGCGCAGCGGTCGGGCTGAACGGGGGGTTCGTGCACACAGCCCAGCTTGGAGCGAACGACCTACACCGAACTGAGATACCTACAGCGTGAGCTATGAGAAAGCGCCACGCTTCCCGAAGGGAGAAAGGCGGACAGGTATCCGGTAAGCGGCAGGGTCGGAACAGGAGAGCGCACGAGGGAGCTTCCAGGGGGAAACGCCTGGTATCTTTATAGTCCTGTCGGGTTTCGCCACCTCTGACTTGAGCGTCGATTTTTGTGATGCTCGTCAGGGGGGCGGAGCCTATGGAAAAACGCCAGCAACGCGGCCTTTTTACGGTTCCTGGCCTTTTGCTGGCCTTTTGCTCACATGTTCTTTCCTGCGTTATCCCCTGATTCTGTGGATAACCGTATTACCGCCTTTGAGTGAGCTGATACCGCTCGCCGCAGCCGAACGACCGAGCGCAGCGAGTCAGTGAGCGAGGAAGCGGAAGAGCGCCCAATACGCAAACCGCCTCTCCCCGCGCGTTGGCCGATTCATTAATGCAGCTGGCACGACAGGTTTCCCGACTGGAAAGCGGGCAGTGAGCGCAACGCAATTAATGTGAGTTAGCTCACTCATTAGGCACCCCAGGCTTTACACTTTATGCTTCCGGCTCGTATGTTGTGTGGAATTGTGAGCGGATAACAATTTCACACAGGAAACAGCTATGACCATGATTACGCCAAGCGCGCAATTAACCCTCACTAAAGGGAACAAAAGCTGGGTACCGGGCCCCCCCTCGAGAGTC
